# Electrochemical oxidative amidation of alcohols towards *N*-acyl azoles as precursors for amides, esters and thioesters

**DOI:** 10.1039/d6ob00847j

**Published:** 2026-09-16

**Authors:** Imke M. A. Bartels, Martin D. Witte, Adriaan J. Minnaard

**Affiliations:** a Stratingh Institute for Chemistry, University of Groningen Nijenborgh 7 9747 AG Groningen The Netherlands M.D.Witte@rug.nl A.J.Minnaard@rug.nl

## Abstract

In the presence of azoles, the selective electrochemical oxidation of alcohols using 4-acetamido-TEMPO (ACT) as a mediator produces *N*-acyl azoles in good yields. These *N*-acyl azoles can undergo further transformation to amides, esters, and thioesters. The electrochemistry minimizes mediator use, and as the supporting electrolyte can be largely recovered and reused, it offers a clear sustainability advantage.

## Abstract 2

In the presence of azoles, the selective electrochemical oxidation of alcohols using 4-acetamido-TEMPO (ACT) as a mediator produces *N*-acyl azoles in good yields.

## Graphical abstract

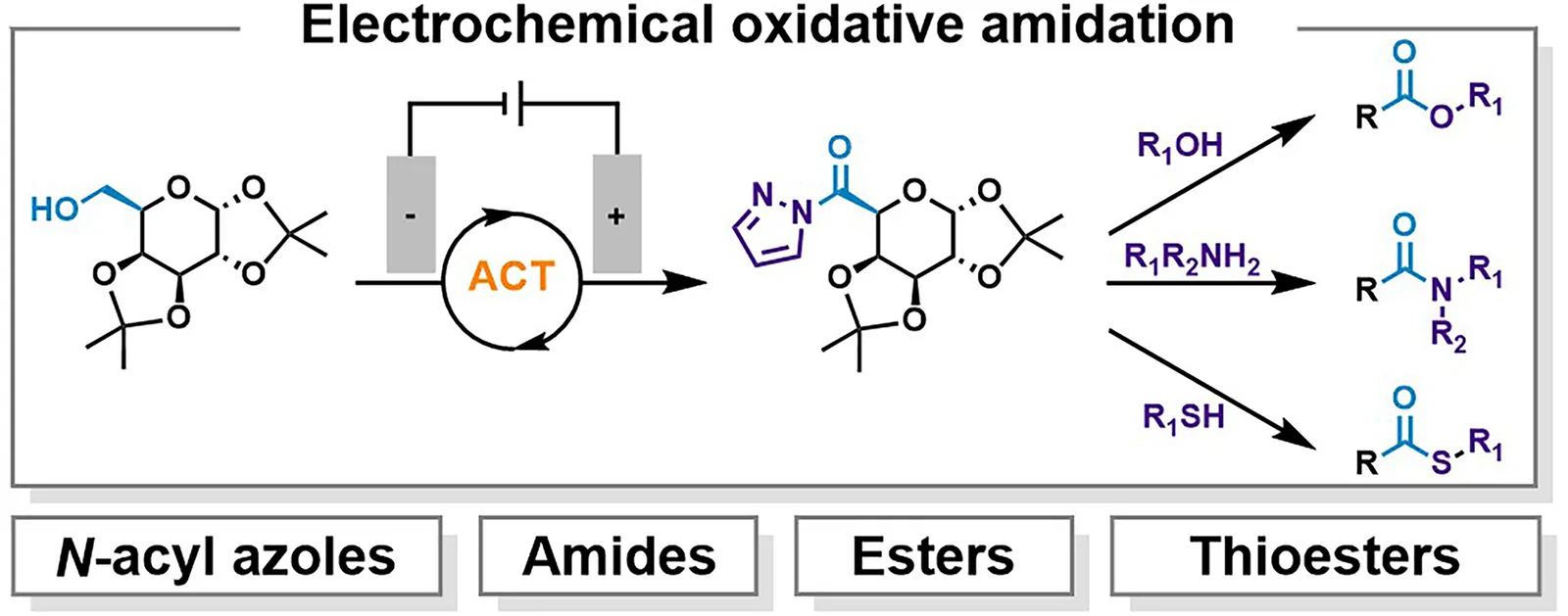

## Introduction

Amides, esters, and thioesters are among the most important functional groups in biology and chemistry.^1^ Many of the clinically approved synthetic and naturally derived pharmaceuticals, as well as numerous chemical probes, contain at least one amide group.^2,3^ Esters and thioesters are frequently employed in industry as fragrances, medicines, and for polymer construction,^4^ as well as being omnipresent in biochemical processes.^5–7^ Therefore, the synthesis of these functional groups remains one of the most frequently performed chemical transformations. For example, amide bond formation accounts for approximately 16% of all reactions used in the synthesis of new drugs.^8,9^

**Fig. 1:**
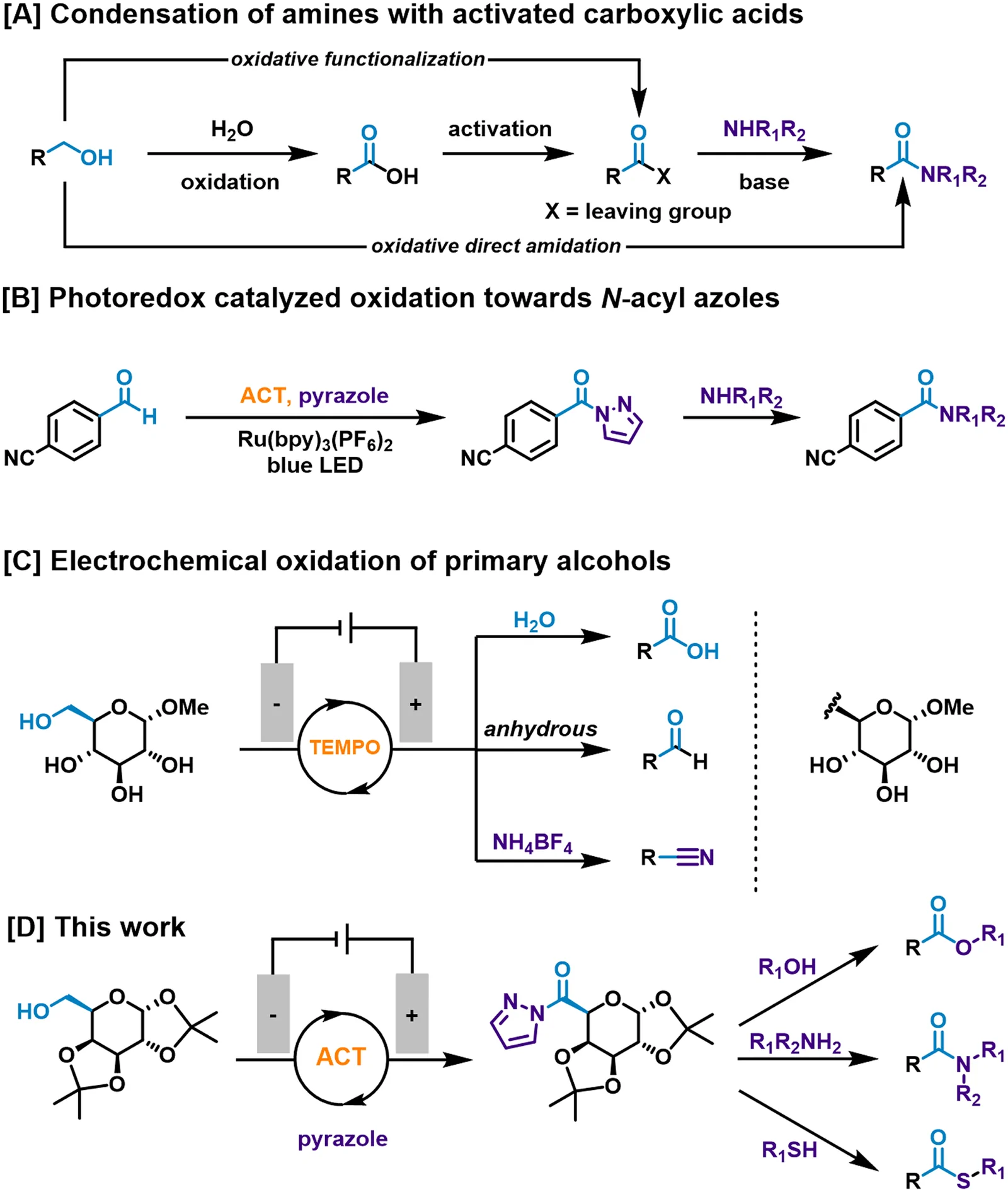
Context and strategy for the oxidative amidation towards *N*-acyl azoles and derivatization.

Conventionally, amides, esters, and thioesters (Fig. 1A) are prepared by condensation of carboxylic acids with thiols, amines, and alcohols. Although direct condensation by heating is sometimes feasible, the more commonly used approach is to activate the carboxylic acid either *in situ* or *via* pre-functionalization.^10,11^ The carboxylic acids employed in these condensation reactions are in turn obtained by oxidizing the corresponding primary alcohols and aldehydes, a transformation for which a wide variety of reported stoichiometric and catalytic methods is available nowadays.^12–19^ Oxoammonium salts (*e.g.* Bobbitt's salt), or their nitroxyl radical precursors, such as 2,2,6,6-tetramethylpiperidine 1-oxyl (TEMPO),^20^ are among the most widely utilized oxidants due to their favorable electron-transfer kinetics, chemical robustness, and high selectivity in oxidative transformations.

A major downside of this reaction sequence, depicted in Fig. 1A, is the step count and limited atom efficiency. Oxidative functionalization reactions, on the other hand, allow the direct conversion of alcohols into the desired carboxylic acid derivatives by combining the oxidation of the alcohol with the condensation reaction. Consequently, these reactions have the potential to increase the synthetic efficiency. Esters,^21^ amides,^22^ but also nitriles,^23,24^ have been prepared directly from aldehydes and alcohols by means of oxidative functionalization reactions. However, these approaches face significant challenges, as competing side reactions must be avoided. In oxidative amidation reactions,^25–27^ for example, the low oxidation potential of amines can lead to preferential oxidation of the amine over the alcohol substrate. Moreover, the intermediate aldehydes may form imines, iminium ions, or enamines, which are potential reaction dead ends or result in decomposition. A similar limitation is observed in ester formation, where the mediator TEMPO can also oxidize the alcohol coupling partner,^28^ particularly in the case of primary alcohols, resulting in reduced selectivity and reactivity. Other limitations of these oxidative functionalization reactions are that the reaction conditions to prepare amides, esters or thioesters are not universal and that large amounts of sacrificial oxidants are used.

In the past decade, various research groups started to investigate oxidative functionalization reactions that yield activated carboxylic acid derivatives, rather than amides or (thio)esters directly.^29–36^ The major advantage of this approach is that the route is more general. A single set of conditions can be used to prepare building blocks that can be converted into amides, esters or thioesters at a later stage. Essential to the success of the approach is that the activated carboxylic acid derivative does not react further during the oxidative functionalization reaction. *N*-acyl azoles, conventionally synthesized by acylating azoles^37,38^ or by condensing hydrazides with acetylacetone,^39,40^ have been shown to have the right balance between reactivity and stability. Pioneering work by Leadbeater and co-workers demonstrated the feasibility of preparing *N*-acyl azoles through the direct oxidative coupling of alcohols with azoles and established that these activated carboxylic acid derivatives are sufficiently stable to be isolated and applied in various transformations, including native chemical ligation, ester formation, and amide formation.^41^ Alcohols and aldehydes could be converted into *N*-acyl azoles using superstoichiometric amounts of Bobbitt's salt.^32,42^ The ACT loading could be lowered by photochemically generating the oxoammonium salt (Fig. 1B), when sodium persulfate was added as a the terminal oxidant.

The reported photocatalytic ACT-mediated synthesis of *N*-acyl azoles from alcohols, raised the idea to develop an electrochemical variant of this reaction. The discussion about the pros and cons of electrochemistry *versus* photochemistry is both complicated and complex,^43^ but electrochemical methods offer distinct practical benefits, particularly if the supporting electrolyte can be efficiently recycled through precipitation and filtration. A major advantage of electrochemical oxidation methods is that these can be performed oxidant-free, as is nicely illustrated by the electrophotocatalytic iron(iii) chloride-mediated coupling of pyrazoles with aldehydes by Noël and co-workers.^44^

The electrochemical oxidation of alcohols with TEMPO is well established. It has long been known that primary alcohols can be oxidized to carboxylic acids under aqueous conditions,^45–47^ or selectively to aldehydes^48^ in the absence of water. In addition, TEMPO-mediated electrochemical systems enable the oxidation of secondary alcohols to ketones.^49^ More recently, these methodologies have been extended to achieve the direct conversion of primary alcohols to nitriles (Fig. 1C).^24^ Building on these precedents, we questioned whether electrochemical TEMPO-mediated oxidation could be further advanced to access activated carboxylic acid derivatives directly from alcohols. Achieving such a transformation would significantly expand the synthetic utility of electrochemical oxidation, providing a practical and broadly applicable route to valuable intermediates. Herein, we report a method for the selective electrochemical conversion of alcohols to *N*-acyl azoles, and demonstrate their versatility as activated carboxylate derivatives (Fig. 1D). The procedure was successfully applied to aliphatic and benzylic alcohols. The reaction is oxidant-free, and the supporting electrolyte can be efficiently recovered and reused.

## Results and discussion

**Fig. 2:**
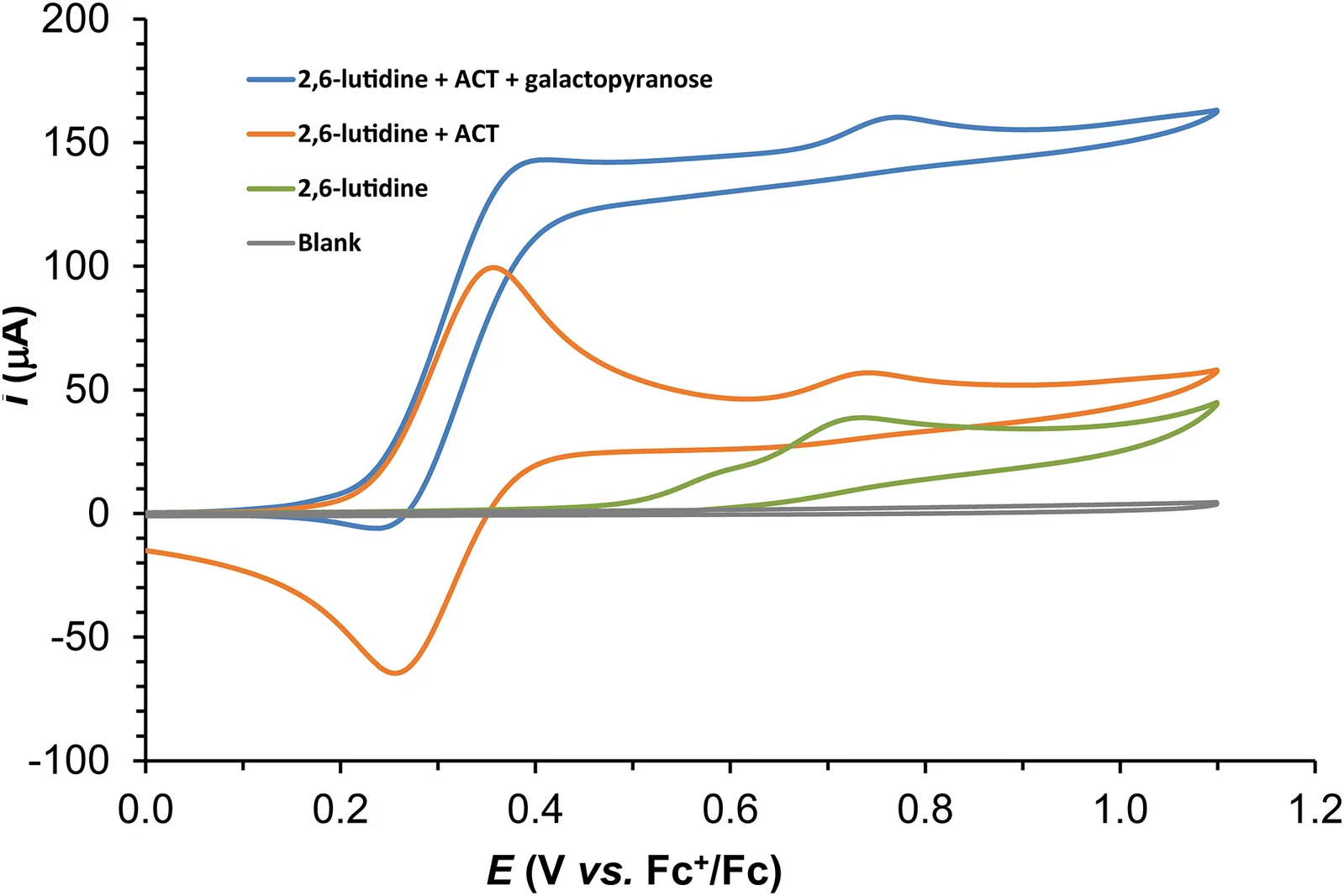
CVs (100 mV s^−1^) in MeCN and TBAPF_6_ (0.1 M) of 2,6-lutidine (450 mM) (green trace); 2,6-lutidine (450 mM) & ACT (2 mM) (orange trace); 2,6-lutidine (450 mM) & ACT (2 mM) & 1,2:3,4-di-*O*-isopropylidene-α-d-galactopyranose 1g (100 mM) (blue trace).

The feasibility of the electrochemical oxidative amidation was initially evaluated by cyclic voltammetry (CV) studies. Fig. 2 shows the cyclic voltammogram of ACT in the presence of pyrazole and 2,6-lutidine. In the absence of ACT (Fig. 2, green trace), the voltammogram exhibits a single anodic peak corresponding to the irreversible oxidation of 2,6-lutidine at *E*_1/2_ = 750 mV. Upon addition of ACT (Fig. 2, orange trace), a reversible redox feature appears at *E*_1/2_ = 450 mV, corresponding to ACT oxidation. The irreversible oxidation of 2,6-lutidine, therefore, occurs at a significantly higher potential than that of ACT and does not interfere with the reversible ACT redox process. Consequently, by limiting the applied potential below this threshold of 750 mV, selective oxidation of the mediator can be achieved without oxidation of 2,6-lutidine. In the presence of substrate, 1,2:3,4-di-*O*-isopropylidene-α-d-galactopyranose 1g (Fig. 2, blue trace), a catalytic current of the ACT anodic peak is observed, indicating ACT-mediated catalytic oxidation of 1g.

**Fig. 3:**
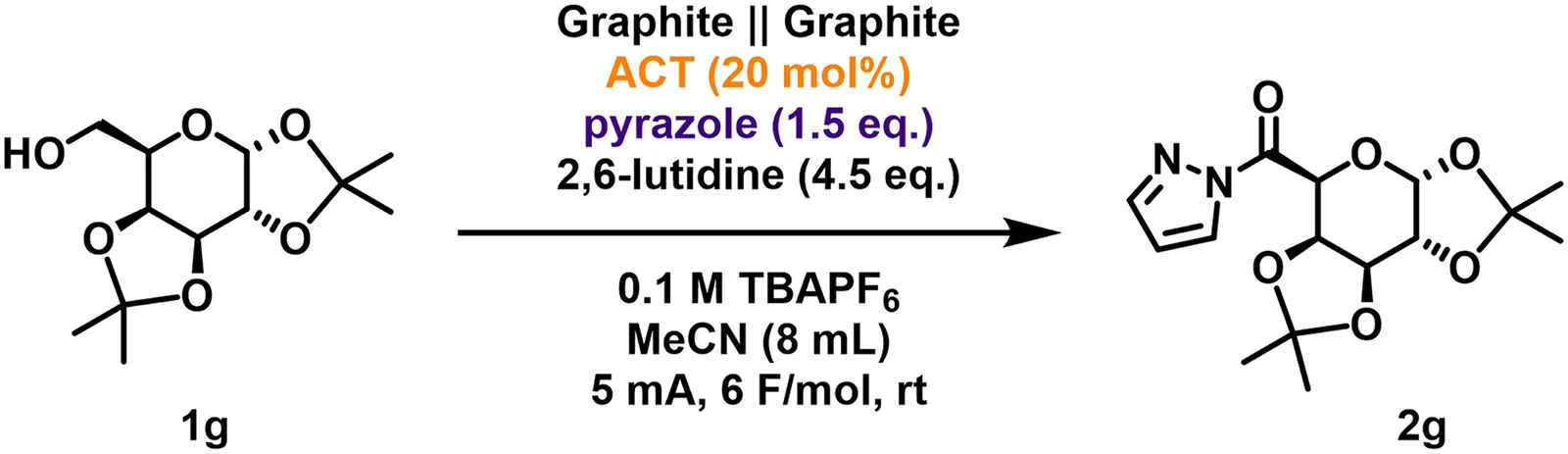
Electrochemical oxidation of 1,2:3,4-di-*O*-isopropylidene-α-d-galactopyranose 1g.

Efforts then shifted to bulk electrolysis to explore preparative conditions for substrate amidation. Initial reactions (Fig. 3) were conducted under conditions closely resembling those reported by Leadbeater *et al.*^32^ for the oxidation using superstoichiometric 4-NHAc-TEMPO^+^BF_4_^−^ (Bobbitt's Salt). In short, ACT as the mediator, pyrazole, and 2,6-lutidine as the base.^28^ Tetrabutylammonium hexafluorophosphate (TBAPF_6_) was added as the supporting electrolyte, and the solvent was switched from dichloromethane to acetonitrile. Electrolysis was conducted using constant-current electrolysis (CCE) with a graphite plate as the anode and cathode. Evaluation of the reaction of 1g showed that complete conversion required passing 6 F mol^−1^ of charge. We hypothesize that ACT first mediates the oxidation of the alcohol to the corresponding aldehyde, which subsequently undergoes condensation with pyrazole to form a hemiaminal intermediate. This intermediate is then further oxidized by ACT to afford the corresponding *N*-acyl azole product.

The reaction conversion and yield obtained from this electrochemical amidation varied significantly between reactions. This variability appeared to result from the incorporation of pyrazole into the graphite electrodes, with the extent of incorporation varying from run to run. Furthermore, the graphite electrodes showed diminished performance after use which could not be fully restored, even after polishing with a 220-grit sanding sponge. Switching to platinum electrodes resolved this issue, and we were pleased to see this resulted in a reproducible 88% quantitative NMR (qNMR) yield of 2g.

For the optimization studies, we switched from the original substrate 1g to 3-phenyl-1-propanol 1a. Electrolysis of 1a under the initial conditions afforded the corresponding *N*-acyl pyrazole in 70% qNMR yield (Table 1, entry 1). Substituting ACT with TEMPO as mediator had no observable difference in reaction outcome (Table 1, entry 2). Varying the pyrazole loading had a small effect on the reaction (Table 1, entries 3 & 4), with 3 eq. proving optimal and affording a 78% qNMR yield. Adjusting the applied constant current to 10 mA had a deleterious effect, leading to only 30% qNMR yield of 2a (Table 1, entry 5). Altering the amount or type of base resulted in a slight decrease in reaction yield (Table 1, entries 6 & 7). At lower ACT loadings, reactions were instead run under CPE conditions, as the observed potentials became too high when using CCE at 5 mA. While lowering the ACT loading still afforded similar qNMR yield (Table 1, entry 8), the reaction time increased substantially under CPE due to the correspondingly lower current. Since increasing the ACT loading likewise led to only a negligible improvement in qNMR yield (Table 1, entry 9), we chose to maintain a loading of 20 mol%, which allowed reactions to be run under CCE.

**Table 1:** Optimization of the bulk electrolysis of 1a

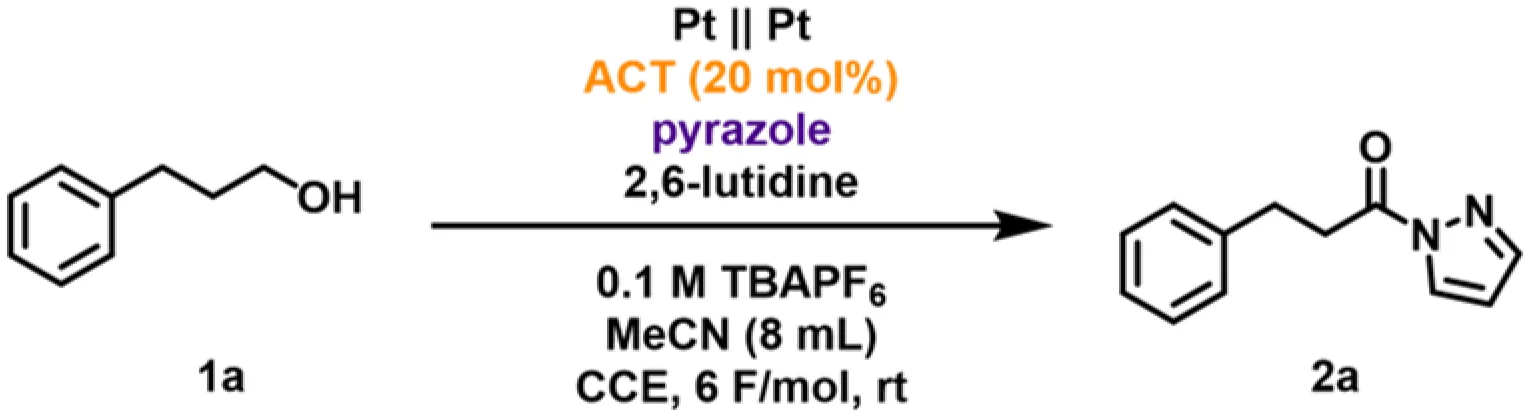					
Entry	Mediator	Pyrazole	Additive	Current	qNMR yield (%)^a^
1	ACT	1.5 eq.	4.5 eq. lutidine	5 mA	70
2	TEMPO	1.5 eq.	4.5 eq. lutidine	5 mA	70
**3**	**ACT**	**3 eq.**	**4.5 eq. lutidine**	**5 mA**	**78**
4	ACT	5 eq.	4.5 eq. lutidine	5 mA	74
5	ACT	1.5 eq.	4.5 eq. lutidine	10 mA	30
6	ACT	1.5 eq.	4.5 eq. pyridine	5 mA	70
7	ACT	1.5 eq.	10 eq. lutidine	5 mA	72
8^b^	ACT (10 mol%)	1.5 eq.	4.5 eq. lutidine	-	73
9	ACT (30 mol%)	1.5 eq.	4.5 eq. lutidine	5 mA	84

a qNMR yields with 3,5-dinitrobenzoate as internal standard.

b Performed *via* CPE instead of CCE, at 0.6 V *vs.* Ag/Ag^+^.

Having established optimal conditions, the scope of the electrochemical oxidation was evaluated across a broad range of alcohol substrates (Table 2). We were very pleased to observe that the developed methodology exhibited a good tolerance across various sterically and electronically different aliphatic and benzylic alcohols, which were converted into their *N*-acyl pyrazoles with good to high yields ranging from 55% to 85% (2a–f). These results are comparable to those reported by Leadbeater *et al.* in the photochemical- and stoichiometric reactions.^32,33^

**Table 2:** Substrate scope for the electrochemical amidation of alcohols

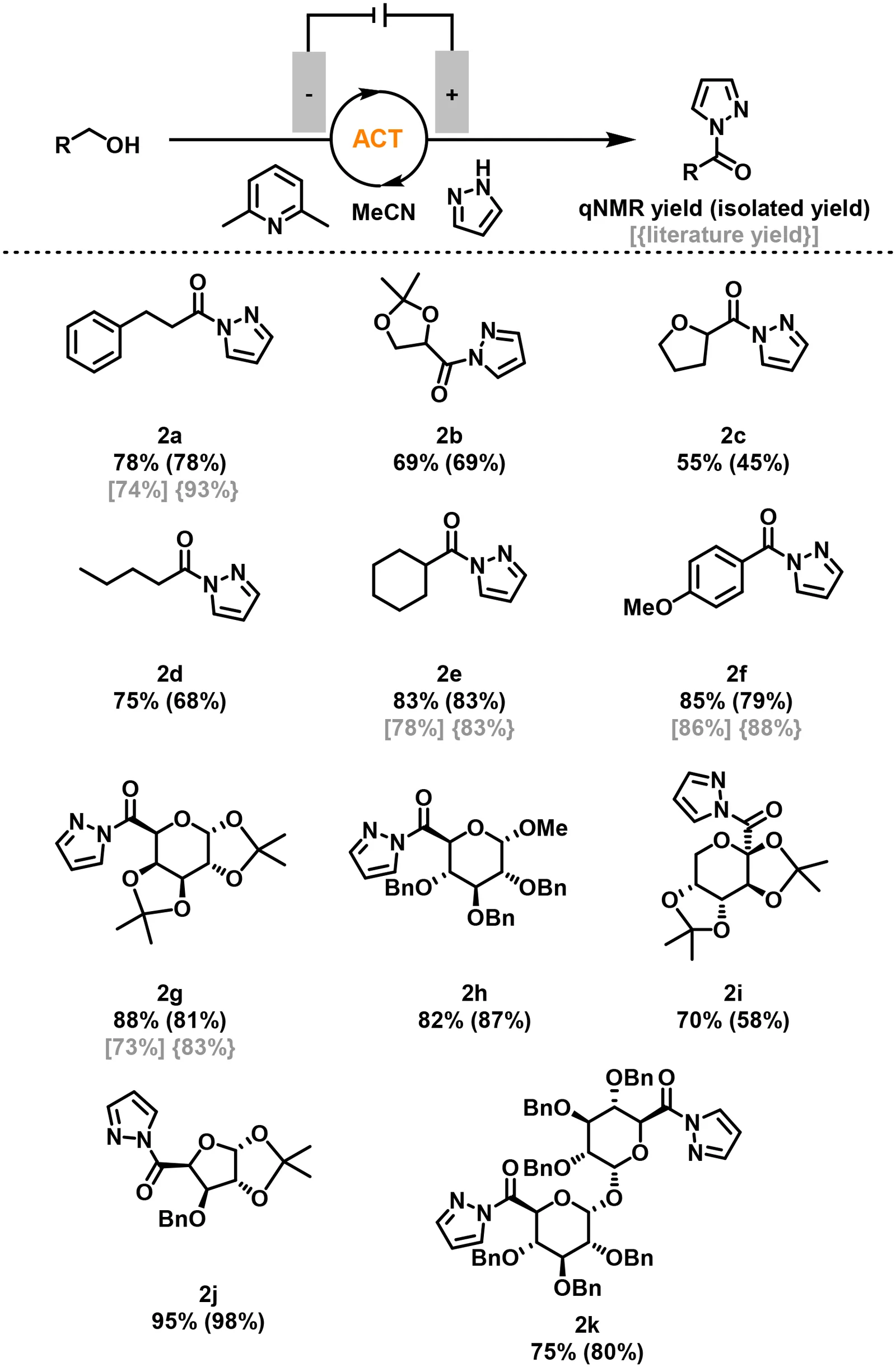

Substrates bearing an α-chiral center, such as solketal 2b, are typically susceptible to racemization, in particular in the aldehyde stage, *via* enolization in the presence of base.^50,51^ Notably, as confirmed by chiral HPLC analysis, no racemization occurred under the applied electrochemical conditions and full retention of enantiomeric purity was preserved (Fig. S38 & S39).

The scope was further expanded to include a wide variety of protected carbohydrates, which serve as important structural building blocks in many biologically relevant molecules. The results show that the stereochemistry of the C4 position, which is close to the reactive site, has little effect on the reaction outcome. Galactoside 2g and glucoside 2h yielded the corresponding *N*-acyl azole in 88% and 82% yield, respectively. Both pyranoses and furanoses were compatible, as fructopyranose 2i and xylofuranose 2j afforded the desired *N*-acyl azole products in good yield. Even disaccharides proved to be effective substrates. Benzyl-protected trehalose 2k underwent oxidation to the corresponding bis(*N*-acyl pyrazole) product in high yield (80%). In this case, the reagent loading and total charge passed were adapted to account for the presence of two primary hydroxyl groups.

It was shortly explored whether our method would be suitable for thioglycoside 2n as well. In carbohydrate chemistry, glycosylation reactions with thioglycosides play an important role, as well as modification reactions of the primary hydroxy group. Oxidation of such substrates to the corresponding carboxylic acids using TEMPO is known,^52,53^ and typically requires a biphasic system (*e.g.*, water/DCM) to suppress undesired oxidation of the thio functionality.^52,54^ We observed that with stoichiometric Bobbitt's salt,^32^2n reached full conversion to the corresponding *N*-acyl pyrazole within 1 h, which expands the scope of this method to an important class of substrates. Under electrochemical conditions, however, the reaction led to just a moderate qNMR yield, and the product lacked purity. This outcome is likely due to competitive oxidation of the thiophenyl moiety, potentially generating a sulfoxide intermediate that acts as a leaving group and leads to substrate decomposition. Nevertheless, the *N*-acyl pyrazole formed was converted to the corresponding piperidyl amide and isolated by column chromatography, in 47% yield and 95% purity (see SI, section 9.3).

It was also briefly explored whether the method would be suitable for substrates bearing an unprotected secondary hydroxyl group. Under the conditions examined, however, no formation of the desired *N*-acyl azole product was observed, and this substrate class was therefore not pursued further at this stage.

To demonstrate the scalability of the reaction, substrate 1g was oxidized on a 1.0 g scale using the same protocol, requiring just a larger reaction vial and a more concentrated reaction mixture (see SI, section 7). Under these conditions, product 2g was obtained in 95% isolated yield, demonstrating the robustness and scalability of the protocol.

Notably, chromatographic purification was generally unnecessary, as the acyl pyrazoles were obtained in high purity after workup. In addition, the workup procedure enabled recovery and subsequent reuse of 95% of the supporting electrolyte (see SI, section 6), minimizing waste generation and reducing the need for fresh electrolyte in repeated runs. Taken together, electrochemical oxidation enables the use of a substoichiometric ACT loading, thereby avoiding the need for superstoichiometric amounts of oxoammonium salts. The requirement to use a supporting electrolyte is mitigated by its efficient recovery and reuse.

In addition, the electrochemical *N*-acylation of other azoles was assessed (Table 3). Prior to preparative experiments, CV studies were conducted to determine the oxidation potentials of the azoles with regard to compatibility with electrochemical ACT oxidation. While most azoles exhibit oxidation potentials higher than that of the mediator, imidazole and indole oxidize at a lower potential and are therefore unsuitable. Based on CV analysis, benzimidazole and 3,5-dimethylpyrazole were found to be compatible with ACT-mediated electrochemical oxidation (Fig. S2 & S3). Both azoles afforded the corresponding amide products, 2l & 2m, in high yields (Table 3).

**Table 3:** Electrochemical oxidative amidation of 1g with azoles

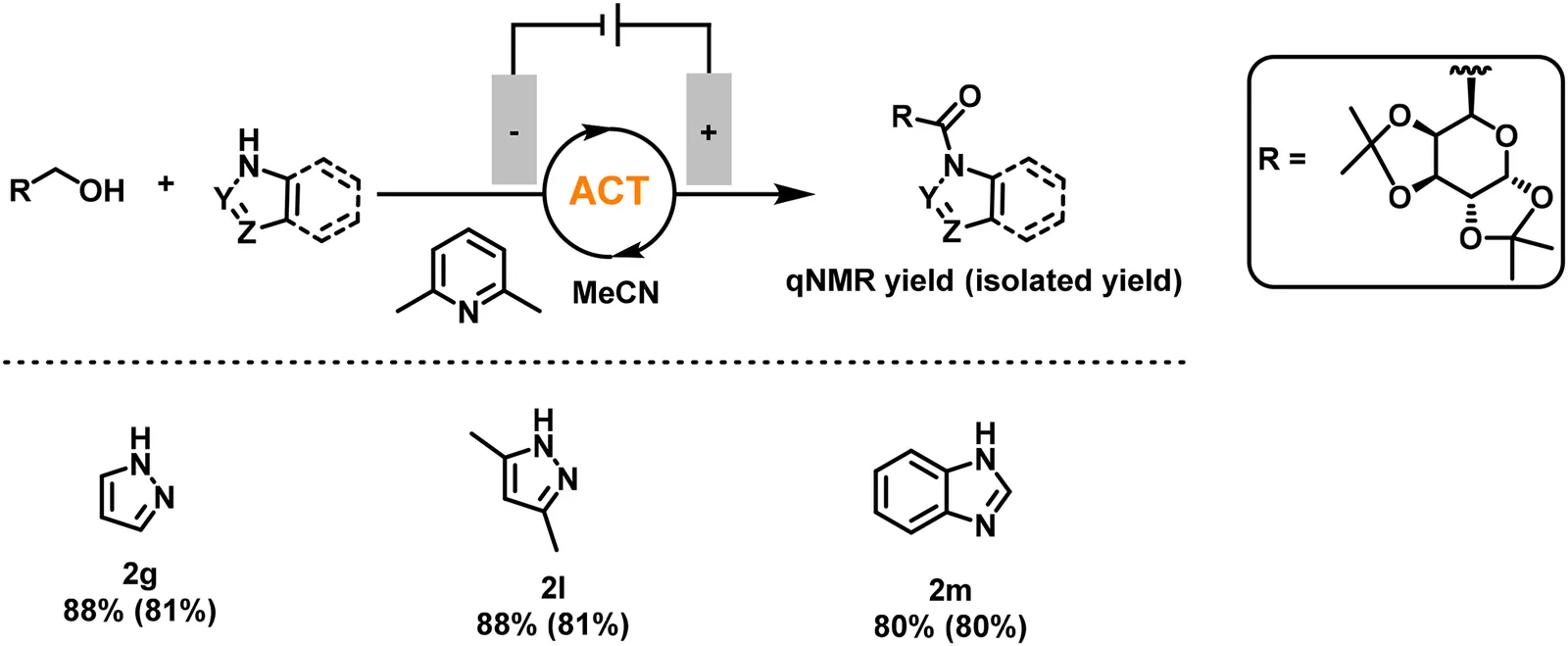

Finally, to demonstrate the chemical space now available through the *N*-acyl azoles, we conducted further experiments using 2g as a representative *N*-acyl azole (Table 4). While literature reports have shown that secondary and tertiary amides can be prepared from *N*-acyl azoles, those procedures require the use of a base such as Et_3_N and often involve additional purification steps. In contrast, our approach proceeds without any additional base, is significantly faster, and allows direct use of the crude *N*-acyl azole without prior work-up, highlighting the efficiency and practicality of this methodology. The crude *N*-acyl azole 2g can be easily converted *via* transamidation into another amide by adding the desired amine to the crude *N*-acyl pyrazole, forming the corresponding amide in excellent yield. This works very well for both primary, 4g, and secondary amines, 3g. *N*-acyl azole 2g could also be converted to primary amide 5g using ammonia with excellent yield. Direct oxidation with ammonia is considered to be inefficient and impractical, due to the need for high pressure, elevated temperature, long reaction time, or formation of complex mixtures.^55,56^ By substituting the amine nucleophile with an alcohol, we were able to access the corresponding carbohydrate ester 6g. As alcohols are less nucleophilic than amines, this required a catalytic amount of acid and 40 °C, but yielded the desired ester in high yield. Following a similar logic, we successfully extended the scope to the synthesis of thioester 7g. Similar to esterification, the formation of the thioester required a catalytic amount of acid.

**Table 4:** Functionalization reactions of 2g

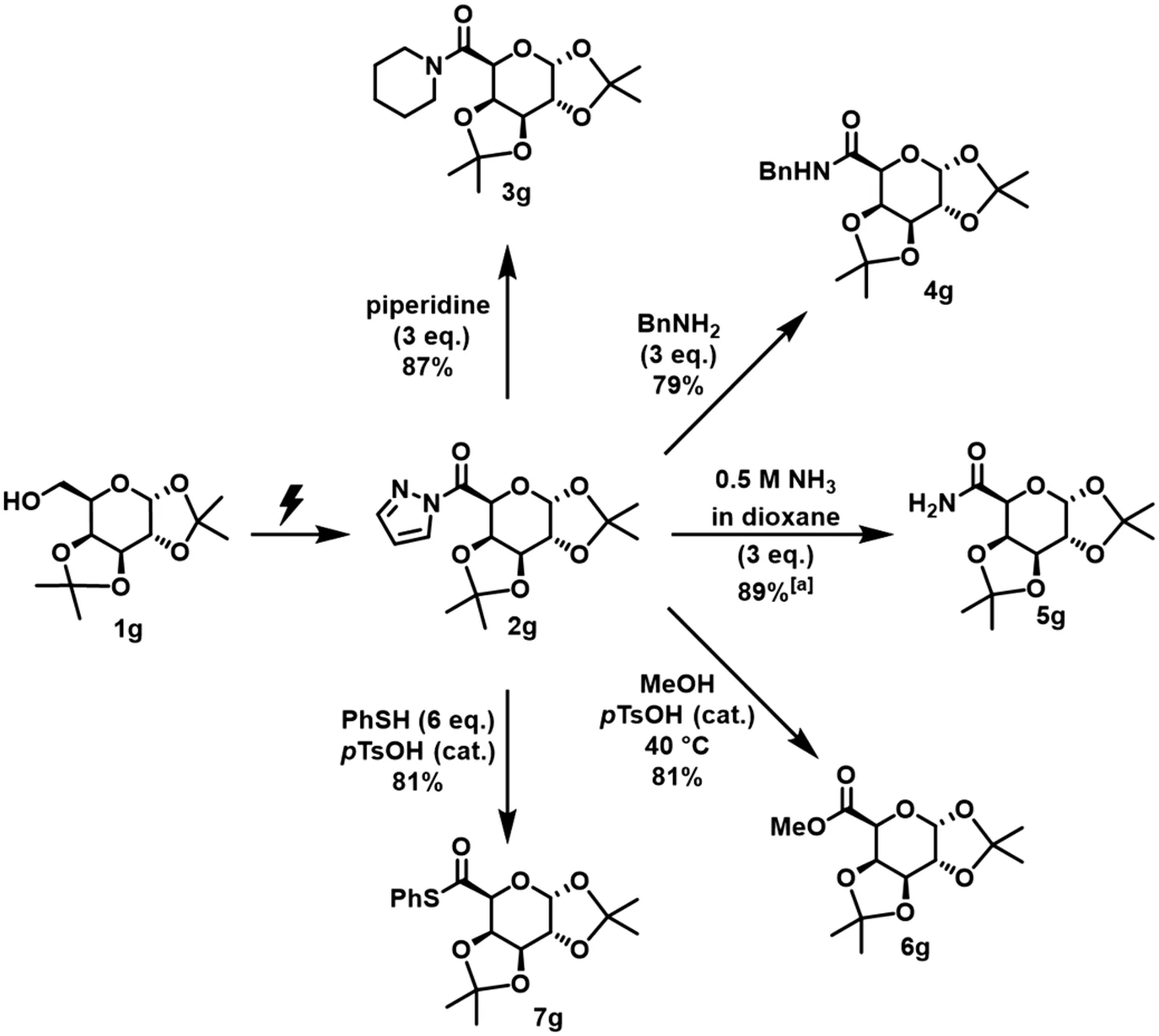

a ∼5% impurity present.

## Conclusions

The results described herein have led to an ACT-mediated electrochemical method for the selective oxidative amidation of alcohols to *N*-acyl azoles, which serve as activated acid intermediates. The use of platinum electrodes allows for high reproducibility and yields across a broad range of substrates, including aliphatic and benzylic alcohols, as well as a range of different protected mono- and disaccharides. The *N*-acyl azoles could be isolated in high purity by simple extraction, thereby avoiding column chromatography. Furthermore, this workup procedure allows the recovery and reuse of 95% of the supporting electrolyte, further enhancing the sustainability of the methodology. The obtained *N*-acyl azoles could be functionalized to a range of amides, esters and thioesters with excellent yields.

## Author contributions

I. M. A. Bartels conceived the idea, designed the study, conducted the synthesis, electrochemical experiments and characterization. M. D. Witte and A. J. Minnaard guided and supervised the research. I. M. A. Bartels prepared the manuscript, and all authors discussed the results and edited the manuscript.

## Conflicts of interest

There are no conflicts to declare.

## Supplementary Material

OB-024-D6OB00847J-s001

## Data Availability

The data supporting this article have been included as part of the supplementary information (SI). Supplementary information: experimental procedures, cyclic voltammetry (CV) studies, HPLC analysis, and ^1^H and ^13^C-NMR spectra, optical rotation, melting point and HRMS data of all new products (PDF). See DOI: https://doi.org/10.1039/d6ob00847j.
