# Supplementary material for: Electrochemical oxidative amidation of alcohols towards N-acyl azoles as precursors for amides, esters and thioesters
Source: Org Biomol Chem. 2026 Sep 16;24(37):7828–34. doi: 10.1039/d6ob00847j (PMC13588081; doi:10.1039/d6ob00847j)
Supplement: Supplementary file 1 [file OB-024-D6OB00847J-s001.pdf]

Supplementary Information (SI) for Organic & Biomolecular Chemistry.
This journal is © The Royal Society of Chemistry 2026
Supplementary Information (SI) for Organic & Biomolecular Chemistry.
This journal is © The Royal Society of Chemistry

Supporting Information

Electrochemical oxidative amination of alcohols towards *N*-acyl azoles as precursors for amides, esters and thioesters

Imke M. A. Bartels,[a] Martin D. Witte,*,[a] Adriaan J. Minnaard*,[a]

[a] Stratingh Institute for Chemistry, University of Groningen, Nijenborgh 7, 9747 AG Groningen, The Netherlands. E-mail M.D.Witte@rug.nl , A.J.Minnaard@rug.nl

Table of contents

1. General information 2
2. General procedure for the electrochemical oxidative amination of alcohols 2
2.1 Electrochemical oxidation 2
2.2 Isolation of the product by extraction 3
2.3 Isolation of the product by column chromatography 3
3. Synthesis of amides via *N*-acyl azole intermediates 3
4. Synthesis of esters via *N*-acyl azole intermediates 3
5. Synthesis of thioesters via *N*-acyl azole intermediates 4
6. Screening mediator loading 4
7. Electrolyte recovery and recyclability 4
8. Large scale reaction 5
9. Cyclic voltammetry (CV) studies 5
9.1 CV study using pyrazole 6
9.2 CV study using 3,5-dimethylpyrazole 6
9.3 CV study using benzimidazole 7
9.4 CV study using imidazole 7
9.5 CV study using indole 8
10. Characterization of the products 8
10.1 *N*-acyl pyrazoles 8
10.2 Functionalization reactions 14
10.3 Electrochemical oxidation of a thioglycoside 16
10.4 Evaluation of racemization at the α-chiral center 17
11. Analysis data 19
11.1 ^{1}H and ^{13}C-NMR spectra of the isolated compounds 19
11.2 NMR spectra recycled electrolyte 52

11.3 HRMS and TLC-MS spectra 54
11.4 HPLC chromatogram 58
References 58

1. General information

All solvents were purchased from commercially available sources and used without further purification. All ammonium salts, pyrazoles, bases, carbohydrates and mediators were purchased from Sigma-Aldrich, TCI, Biosynth and Combi-Blocks. All chemicals were used without further purification. Electrochemical reactions were carried out using a Squidstat Prime Potentiostat from Admiral Instruments. The graphite (Graphite SK-50) and platinum (Platinum Foil) electrodes were purchased from IKA. Graphite electrodes were cleaned by sonication in deionized water for 5 min, then polished with a 220-sanding sponge and rinsed with acetone or deionized water and dried in air. Platinum electrodes were sonicated in deionized water for 2 min, then rinsed with acetone and dried in air. The reference electrodes were only rinsed with acetone and dried in air.

NMR spectra (^{1}H and ^{13}C) were obtained with either a Bruker Avance Neo 600 MHz spectrometer, a Varian AMX400 spectrometer or a JEOL 400 MHz spectrometer with reference to residual solvent signals: $CDCl_3$ signal at 7.26 ppm (^{1}H) and 77.16 ppm (^{13}C). All data is reported as follows: Chemical shifts (δ), multiplicity (s = singlet, d = doublet, dd = doublet of doublets, t = triplet, q = quartet, m = multiplet) coupling constant *J* (Hz), and integration. Voltametric experiments were performed using a Squidstat Prime Potentiostat. High Resolution Mass Spectra (HMRS) were obtained using an LTQ Orbitrap XL. TLC-MS spectra were obtained using Advion expression® compact mass spectrometer (CMS) in combination with an Advion plate express® with methanol as the mobile phase. Normal phase chiral HPLC analysis was performed on a Shimadzu LC-20 series equipped with a photodiode array detector. Chromatography was performed using an automated Büchi Pure C-850 FlashPrep. Specific rotations were determined with a Schmidt + Haensch Polartronic MH8 polarimeter in a 100 mm path-length cell.

2. General procedure for the electrochemical oxidative amination of alcohols

2.1 Electrochemical oxidation

Reactions were carried out using an undivided cell equipped with a platinum plate anode and cathode (sheet, 5.25 cm x 0.8 cm x 0.1 mm ~ 3 cm was immersed in the solution) and a $Ag/AgNO_3$ reference electrode.

In a 10 mL vial, the starting material (0.5 mmol, 1 equiv), pyrazole (1.5 mmol, 3 equiv), 2,6-lutidine (2.25 mmol, 4.5 equiv), and ACT (20 mol%) were added in an 8 mL solution of 0.1 M tetrabutylammonium hexafluorophosphate ($TBAPF_6$) in MeCN. The mixture was stirred for 2 min before the vial was capped and equipped with the electrodes. Constant current electrolysis (5 mA, j = 1.1 mA/cm^2) was conducted at room temperature, with stirring at 1000 rpm and 6 F/mol of charge was passed. The reaction was stopped after 6 F/mol of charge had passed or the limit of 1.5 V (vs Ag/Ag^+) was exceeded. At the end of the reaction, methyl 3,5-dinitrobenzoate (0.5 mmol, 1 equiv) was added as an internal standard to the reaction mixture and a sample was taken for qNMR analysis in $CDCl_3$. Alternatively, the crude reaction mixture was purified as described in sections 2.2 or 2.3. *

** Note: qNMR analysis and product isolation were performed on different reaction batches. Therefore, a deviation of up to 5% may be observed between qNMR yields and isolated yields. This occasionally resulted in isolated yields that were higher than the corresponding qNMR yields.*

2.2 Isolation of the product by extraction

The crude reaction mixture was concentrated *in vacuo*. Et_2O (~ 5 mL) was added, causing precipitation of the electrolyte, $TBAPF_6$. The mixture was filtered through a coarse-porosity fritted glass funnel. The solids were washed thoroughly with Et_2O (~ 25 mL). The solution was then transferred to a separatory funnel and diluted with aqueous 0.5 M HCl (40 mL). The layers were separated and the aqueous layer was extracted with Et_2O (1 × 25 mL). The organic layers were combined and washed with 0.5 M HCl (1 × 40 mL), saturated aqueous $NaHCO_3$ (40 mL), and brine (40 mL). The organic layer was dried over Na_2SO_4, and the solvent was removed in vacuo to afford the pure N-acyl pyrazole.

2.3 Isolation of the product by column chromatography

The crude reaction mixture was concentrated *in vacuo*. The residue was purified by column chromatography (SiO_2, 0 – 10% EtOAc in pentane) to afford the pure *N*-acyl pyrazole.

3. Synthesis of amides via *N*-acyl azole intermediates

In a 10 mL vial, the starting material (0.5 mmol, 1 equiv), pyrazole (1.5 mmol, 3 equiv), 2,6-lutidine (2.25 mmol, 4.5 equiv), and ACT (20 mol%) were added in an 8 mL solution of 0.1 M tetrabutylammonium hexafluorophosphate ($TBAPF_6$) in MeCN. The mixture was stirred for 2 min before the vial was capped and equipped with the electrodes. Constant current electrolysis (5 mA, j = 1.1 mA/cm^2) was conducted at room temperature, with stirring at 1000 rpm and 6 F/mol of charge was passed. The reaction was stopped after 6 F/mol of charge had passed, or the limit of 1.5 V (vs Ag/Ag^+) was exceeded. At the end of the reaction, the electrodes were removed and rinsed with MeCN.

To the crude reaction mixture, the amine (1.5 mmol, 3 equiv) was added and the reaction was stirred at room temperature until TLC showed complete conversion (10-20% EtOAc in pentane). The crude reaction mixture was concentrated *in vacuo*. Et_2O (~ 5 mL) was added, causing precipitation of the electrolyte, $TBAPF_6$. The mixture was filtered through a coarse-porosity fritted glass funnel. The solids were washed thoroughly with Et_2O (~ 25 mL). The solution was then transferred to a separatory funnel and diluted with aqueous 0.5 M HCl (40 mL). The layers were separated, and the aqueous layer was extracted with Et_2O (1 × 25 mL). The organic layers were combined and washed with 0.5 M HCl (1 × 40 mL), saturated aqueous $NaHCO_3$ (40 mL), and brine (40 mL). The organic layer was dried over Na_2SO_4, and the solvent was removed in vacuo to afford the pure amide.

4. Synthesis of esters via *N*-acyl azole intermediates

In a 10 mL vial, the starting material (0.5 mmol, 1 equiv), pyrazole (1.5 mmol, 3 equiv), 2.6-lutidine (2.25 mmol, 4.5 equiv), and ACT (20 mol%) were added in an 8 mL solution of 0.1 M tetrabutylammonium hexafluorophosphate ($TBAPF_6$) in MeCN. The mixture was stirred for 2 min before the vial was capped and equipped with the electrodes. Constant current electrolysis (5 mA, j = 1.1 mA/cm^2) was conducted at room temperature, with stirring at 1000 rpm and 6 F/mol of charge was passed. The reaction was stopped after 6 F/mol of charge had passed, or the limit of 1.5 V (vs Ag/Ag^+) was exceeded. At the end of the reaction, the electrodes were removed and rinsed with MeCN.

The crude reaction mixture was concentrated *in vacuo* and re-dissolved in 5 mL MeOH. *p*-toluenesulfonic acid (cat.) was added and the mixture was stirred at 40 °C for 16 h. The crude reaction mixture was concentrated *in vacuo*. Et_2O (~ 5 mL) was added, causing precipitation of the electrolyte, $TBAPF_6$. The mixture was filtered through a coarse-porosity fritted glass funnel. The solids were washed thoroughly with Et_2O (~ 25 mL). The solution was then transferred to a separatory funnel and diluted with aqueous 0.5 M HCl (40 mL). The layers were separated and the aqueous layer was extracted with Et_2O (1 × 25 mL). The organic layers were combined and washed with 0.5 M HCl (1 × 40 mL), saturated

aqueous $NaHCO_3$ (40 mL), and brine (40 mL). The organic layer was dried over Na_2SO_4, and the solvent was removed in vacuo to afford the pure ester.

5. Synthesis of thioesters via *N*-acyl azole intermediates

In a 10 mL vial, the starting material (0.5 mmol, 1 equiv), pyrazole (1.5 mmol, 3 equiv), 2.6-lutidine (2.25 mmol, 4.5 equiv), and ACT (20 mol%) were added in an 8 mL solution of 0.1 M tetrabutylammonium hexafluorophosphate ($TBAPF_6$) in MeCN. The mixture was stirred for 2 min before the vial was capped and equipped with the electrodes. Constant current electrolysis (5 mA, j = 1.1 mA/cm^2) was conducted at room temperature, with stirring at 1000 rpm and 6 F/mol of charge was passed. The reaction was stopped after 6 F/mol of charge had passed, or the limit of 1.5 V (vs Ag/Ag$^+$) was exceeded. At the end of the reaction, the electrodes were removed and rinsed with MeCN

To the crude reaction mixture, the thiol (3 mmol, 6 equiv) and *p*-toluenesulfonic acid (cat.) was added, and the reaction was stirred at room temperature for 4 d, until TLC showed complete conversion (20 % EtOAc in pentane). The crude reaction mixture was concentrated *in vacuo*. The residue was purified by column chromatography (SiO_2, 0 – 20% EtOAc in pentane) to afford the pure thioester.

6. Screening mediator loading

Reactions were carried out using an undivided cell equipped with a platinum plate anode and cathode (sheet, 5.25 cm x 0.8 cm x 0.1 mm ~ 3 cm was immersed in the solution) and a $Ag/AgNO_3$ reference electrode.

In a 5 mL vial, the 3-phenyl-1-propanol **1a** (20 mg, 0.15 mmol), pyrazole (31 mg, 0.45 mmol, 3 equiv), 2,6-lutidine (78 µL, 0.675 mmol, 4.5 equiv), and ACT (5 or 10 mol%) were added in an 5 mL solution of 0.1 M tetrabutylammonium hexafluorophosphate ($TBAPF_6$) in MeCN. The mixture was stirred for 2 min before the vial was capped and equipped with the electrodes. Constant potential electrolysis (0.6 V vs Ag/Ag$^+$) was conducted at room temperature, with stirring at 1000 rpm and 6 F/mol of charge was passed. At the end of the reaction, methyl 3,5-dinitrobenzoate (0.15 mmol, 1 equiv) was added as an internal standard to the reaction mixture and a sample was taken for qNMR analysis in $CDCl_3$.

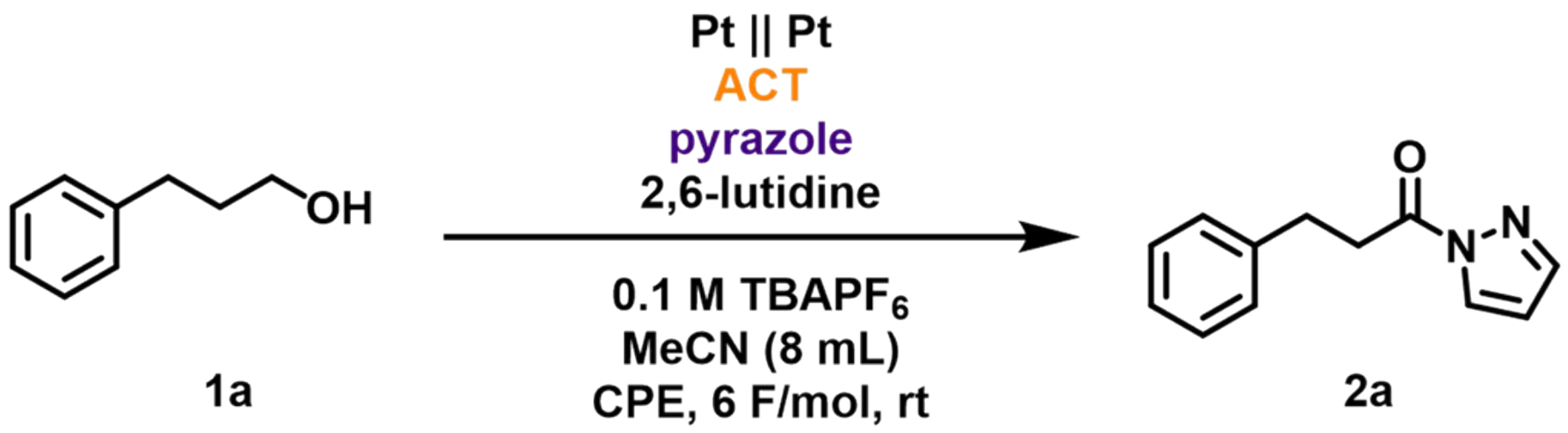

entry	Mediator	Pyrazole	Additive	Potential	qNMR yield (%)
1	ACT (10 mol%)	1.5 eq.	4.5 eq. Lutidine	0.6 V	73
2	ACT (5 mol%)	1.5 eq.	4.5 eq. Lutidine	0.6 V	71

7. Electrolyte recovery and recyclability

The supporting electrolyte, tetrabutylammonium hexafluorophosphate ($TBAPF_6$) was recovered during the workup via precipitation. Following the general procedure detailed in Section 2.2, the electrolyte was isolated as a white crystalline solid with a recovery yield of 95%. The purity of the recovered electrolyte was confirmed by ^{1}H- and ^{19}F-NMR analysis (**Figure S39** & **Figure S40**).To evaluate the performance of the recycled material, a subsequent oxidation of the substrate **1g** was performed

according to the protocol in Section 2.1. Quantitative NMR analysis of the crude reaction mixture revealed a product yield of 85% (**Figure S38**), which is identical to the performance observed using fresh electrolyte.

8. Large scale reaction

The reaction was carried out using an undivided cell equipped with a platinum plate anode and cathode (sheet, 5.25 cm x 0.8 cm x 0.1 mm ~ 3 cm was immersed in the solution) and a Ag/$AgNO_3$ reference electrode.

In a 20 mL vial, 1,2:3,4-di-*O*-isopropylidene-α-D -galactopyranose **1g** (1.0 g, 3.84 mmol), pyrazole (785 mg, 1.5 mmol, 3 equiv), 2,6-lutidine (2 mL, 2.25 mmol, 4.5 equiv), and ACT (20 mol%) were added in an 18 mL solution of 0.1 M tetrabutylammonium hexafluorophosphate ($TBAPF_6$) in MeCN. The mixture was stirred for 2 min before the vial was capped and equipped with the electrodes. Constant current electrolysis (5 mA, j = 1.1 mA/cm^2) was conducted at room temperature, with stirring at 800 rpm and 6 F/mol of charge was passed. The reaction was stopped after 6 F/mol of charge had passed. The crude reaction mixture was concentrated *in vacuo*. Et_2O (~ 50 mL) was added, causing precipitation of the electrolyte, $TBAPF_6$. The mixture was filtered through a coarse-porosity fritted glass funnel. The solids were washed thoroughly with Et_2O (~ 100 mL). The solution was then transferred to a separatory funnel and diluted with aqueous 0.5 M HCl (100 mL). The layers were separated and the aqueous layer was extracted with Et_2O (1 × 50 mL). The organic layers were combined and washed with 0.5 M HCl (1 × 100 mL), saturated aqueous $NaHCO_3$ (100 mL), and brine (100 mL). The organic layer was dried over Na_2SO_4, and the solvent was removed in vacuo to afford the pure *N*-acyl pyrazole as a white solid (1.18 g, 95%). The product was characterized by ^{1}H-NMR and the spectral data match the literature[1] values.

^{1}H-NMR (400 MHz, $CDCl_3$) δ 8.29 (dd, *J* = 2.9 Hz, 1H), 7.71 (dd, *J* = 1.5 Hz, 1H), 6.44 (dd, *J* = 2.9, 1.5 Hz, 1H), 5.76 (d, *J* = 5.0 Hz, 1H), 5.58 (d, *J* = 2.5 Hz, 1H), 4.95 (dd, *J* = 7.5, 2.5 Hz, 1H), 4.71 (dd, *J* = 7.5, 2.9 Hz, 1H), 4.46 (dd, *J* = 5.1, 2.8 Hz, 1H), 1.59 (s, 3H), 1.48 (s, 3H), 1.36 (s, 3H), 1.27 (s, 3H).

9. Cyclic voltammetry (CV) studies

All cyclic voltametric (CV) experiments were carried out using a Squidstat Prime potentiostat. The experiments were carried out with a glassy carbon (GC) working electrode (3 mm diameter) and a platinum wire counter electrode (1.0 cm, spiral wire). The working potentials were measured against a Ag/$AgNO_3$ reference electrode (internal solution, 0.1 M Bu_4NPF_6 and 0.01 M $AgNO_3$ in MeCN). The GC working electrode was polished with alumina powder (5 µ) and water before each experiment. The redox potential values were adjusted relative to Fc^+/Fc and electrochemical studies in organic solvent mixtures were recorded accordingly.

9.1 CV study using pyrazole

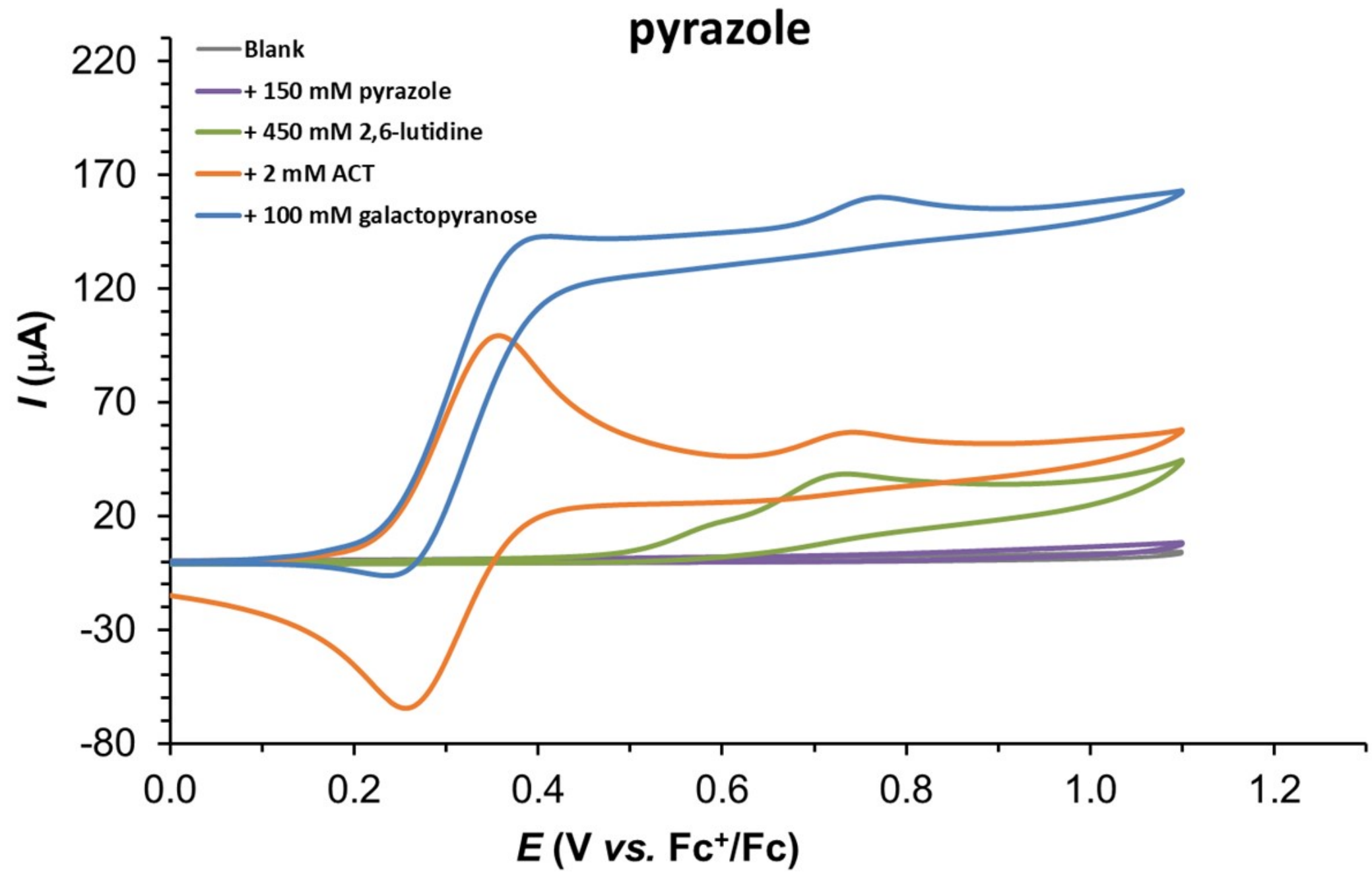

Figure S1: CVs (100 mV/s) in MeCN and $TBAPF_6$ (0.1 M) of pyrazole (150 mM) (purple trace); pyrazole (150 mM) + 2,6-lutidine (450 mM) (green trace); pyrazole (150 mM) + 2,6-lutidine (450 mM) + ACT (2 mM) (orange trace); pyrazole (150 mM) + 2,6-lutidine (450 mM) + ACT (2 mM) & 1,2:3,4-di-*O*-isopropylidene-α-D-galactopyranose **1g** (100 mM) (blue trace). All potentials are reported relative to Fc^+/Fc.

9.2 CV study using 3,5-dimethylpyrazole

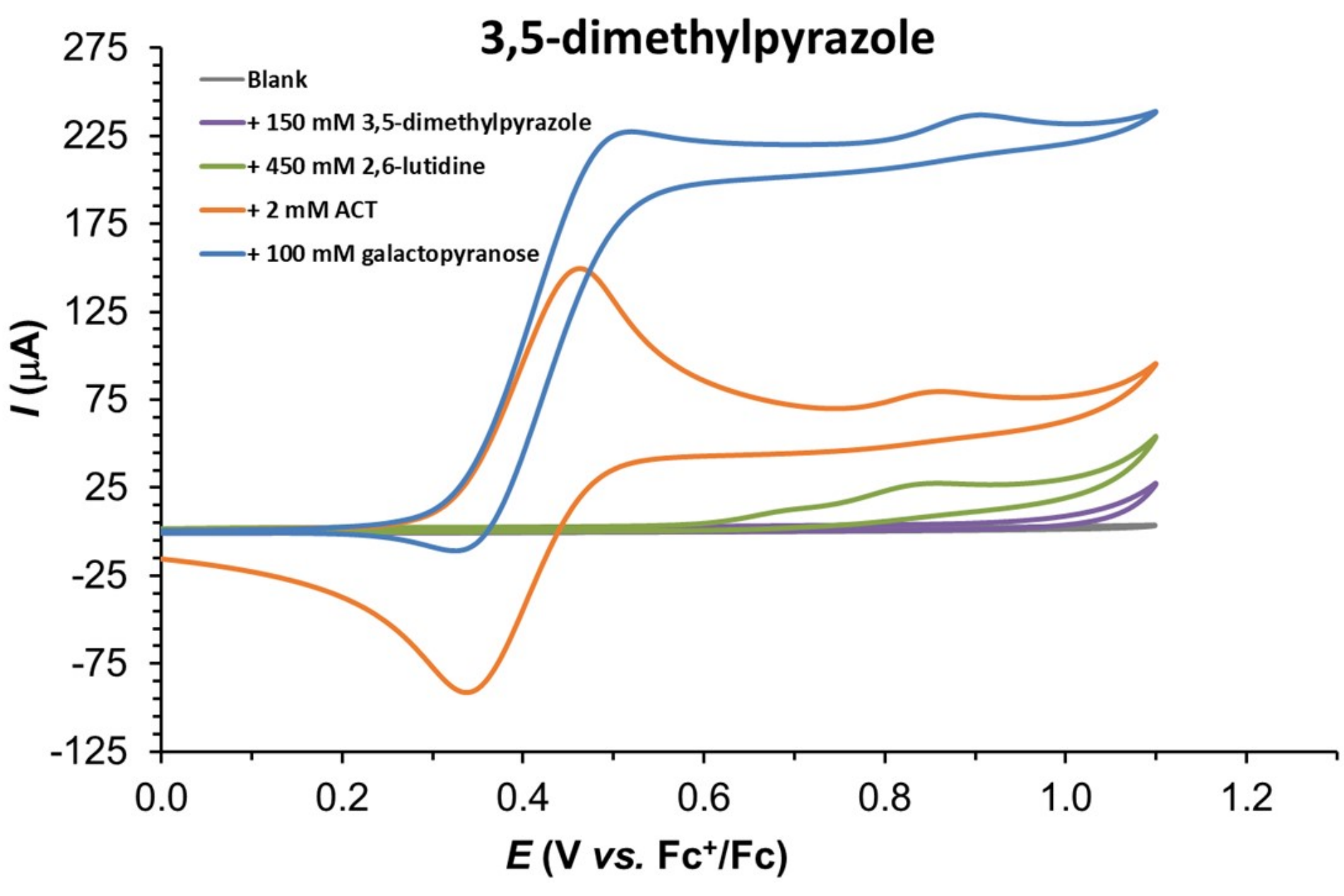

Figure S2: CVs (100 mV/s) in MeCN and $TBAPF_6$ (0.1 M) of 3,5-dimethylpyrazole (150 mM) (purple trace); 3,5-dimethylpyrazole (150 mM) + 2,6-lutidine (450 mM) (green trace); 3,5-dimethylpyrazole (150 mM) + 2,6-lutidine (450 mM) + ACT (2 mM) (orange trace); 3,5-dimethylpyrazole (150 mM) + 2,6-lutidine (450 mM) + ACT (2 mM) & 1,2:3,4-di-*O*-isopropylidene-α-D-galactopyranose **1g** (100 mM) (blue trace). All potentials are reported relative to Fc^+/Fc.

9.3 CV study using benzimidazole

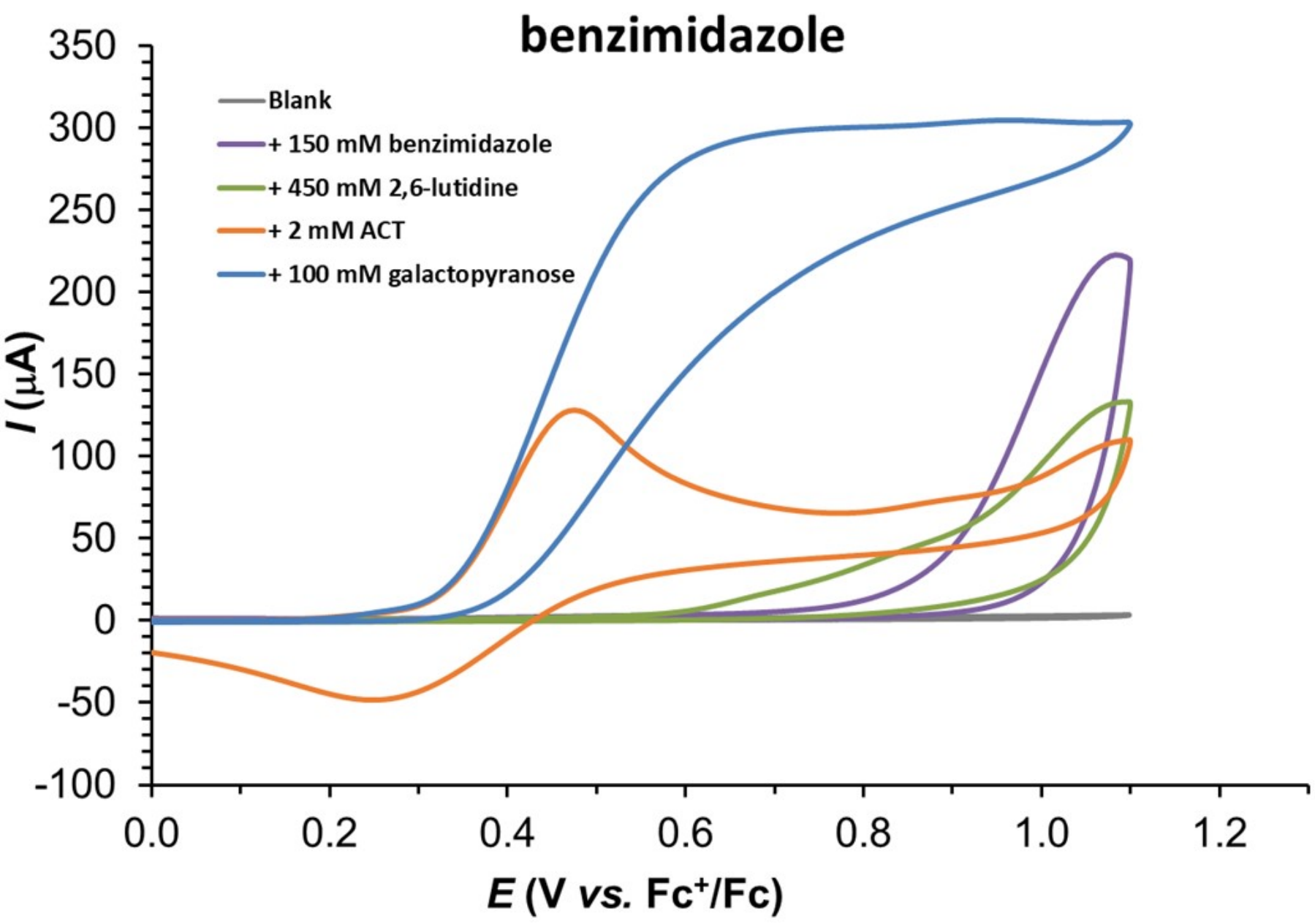

Figure S3: CVs (100 mV/s) in MeCN and $TBAPF_6$ (0.1 M) of benzimidazole (150 mM) (purple trace); benzimidazole (150 mM) + 2,6-lutidine (450 mM) (green trace); benzimidazole (150 mM) + 2,6-lutidine (450 mM) + ACT (2 mM) (orange trace); benzimidazole (150 mM) + 2,6-lutidine (450 mM) + ACT (2 mM) & 1,2:3,4-di-*O*-isopropylidene-α-D-galactopyranose **1g** (100 mM) (blue trace). All potentials are reported relative to Fc^+/Fc.

9.4 CV study using imidazole

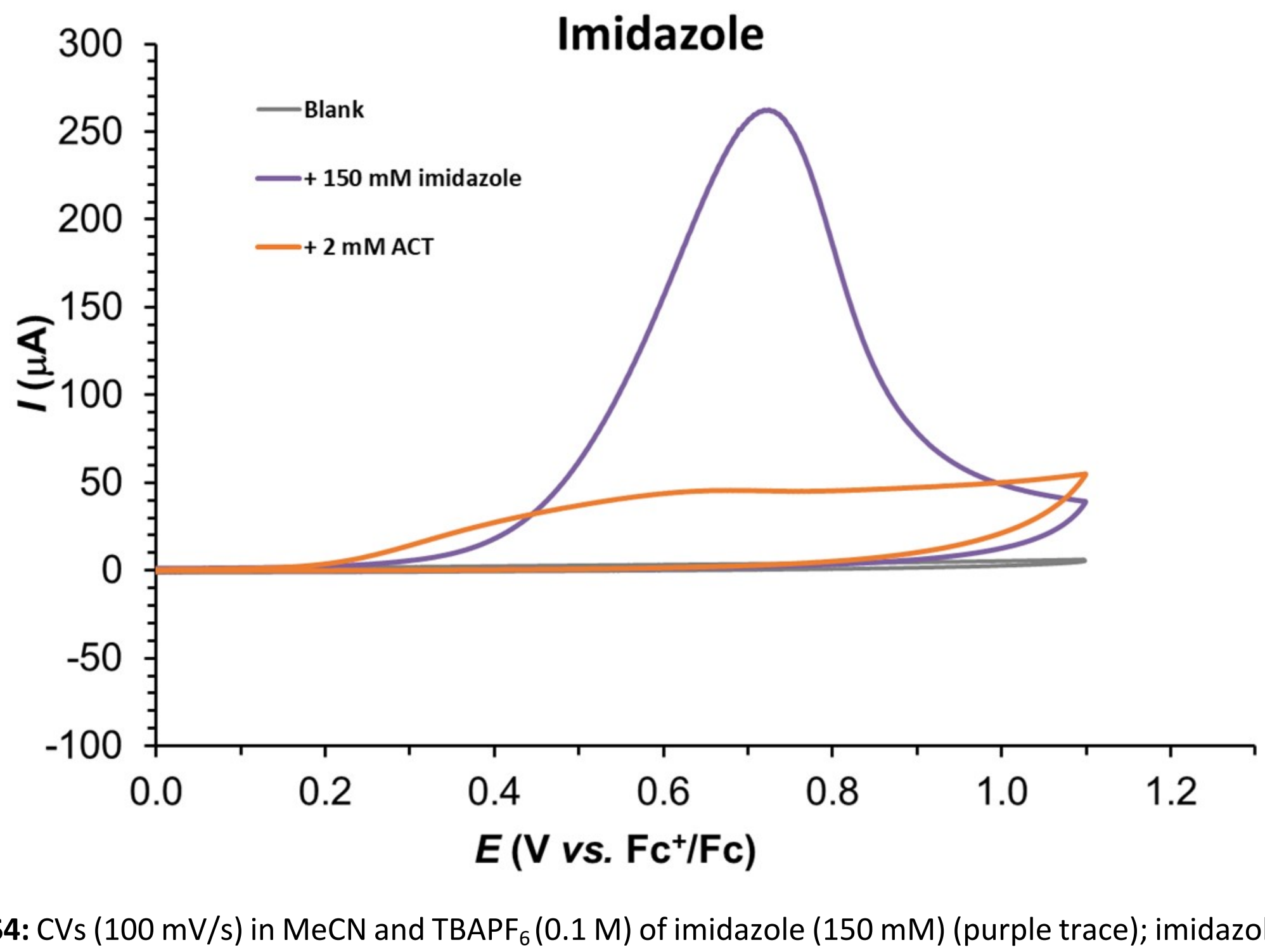

Figure S4: CVs (100 mV/s) in MeCN and $TBAPF_6$ (0.1 M) of imidazole (150 mM) (purple trace); imidazole (150 mM) + ACT (2 mM) (orange trace). All potentials are reported relative to Fc^+/Fc.

9.5 CV study using indole

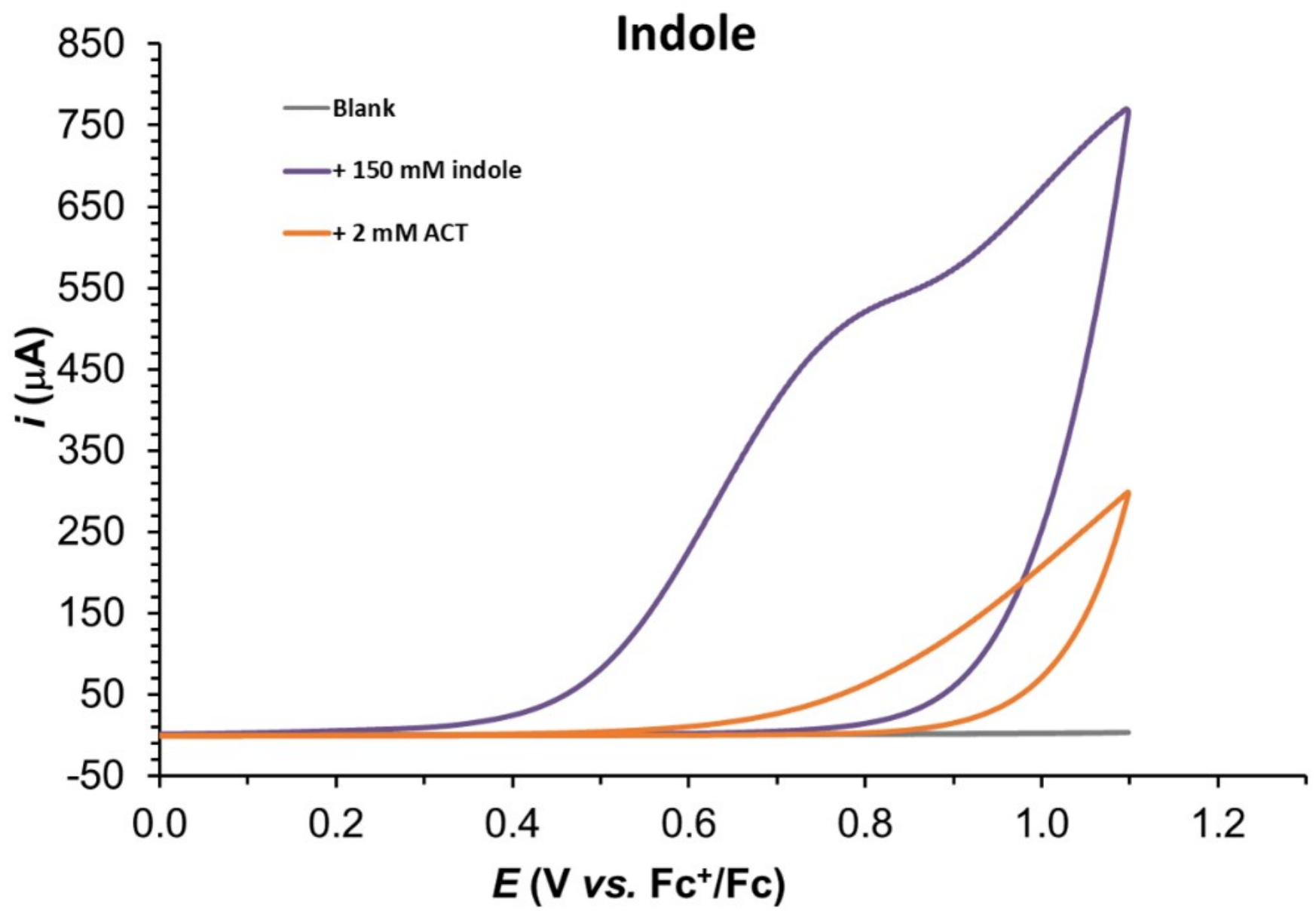

Figure S5: CVs (100 mV/s) in MeCN and $TBAPF_6$ (0.1 M) of indole (150 mM) (purple trace); indole (150 mM) + ACT (2 mM) (orange trace). All potentials are reported relative to Fc^+/Fc.

10. Characterization of the products

10.1 *N*-acyl pyrazoles

3-phenyl-1-(1*H*-pyrazol-1-yl)propan-1-one **2a**

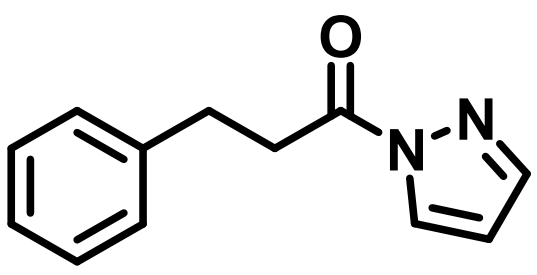

Prepared via the general procedure outlined in section 2.1, from 3-phenyl-1-propanol **1a** (68 mg, 0.5 mmol) and pyrazole (102 mg, 1.5 mmol, 3 equiv). The crude mixture was analyzed by qNMR (78%). The product was isolated via extraction, as described in section 2.2. This afforded the product as a colorless oil (78 mg, 78%). The product was characterized by ^{1}H-NMR and the spectral data match the literature[1] values.

^{1}H-NMR δ 8.23 (d, *J* = 2.9 Hz, 1H), 7.68 (d, *J* = 1.5 Hz, 1H), 7.31 – 7.22 (m, 5H), 6.41 (dd, *J* = 2.9, 1.5 Hz, 1H), 3.46 (t, *J* = 8.1 Hz, 2H), 3.09 (t, *J* = 8.1 Hz, 2H).

(2,2-dimethyl-1,3-dioxolan-4-yl)(1*H*-pyrazol-1-yl)methanone **2b**

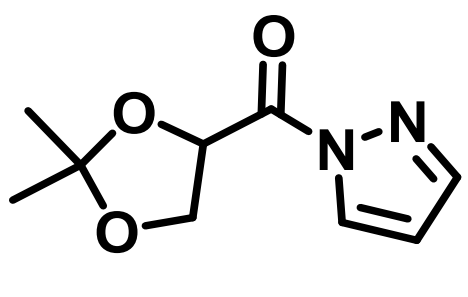

Prepared via the general procedure outlined in section 2.1, from (±)-2,2-dimethyl-1,3-dioxolane-4-methanol **1b** (66 mg, 0.5 mmol) and pyrazole (102 mg, 1.5 mmol, 3 equiv). The crude mixture was analyzed by qNMR (69%). The product was isolated via extraction, as described in section 2.2. This

afforded the product as a colorless oil (67 mg, 69%). The product was characterized by ^{1}H-NMR and the spectral data match the literature[1] values.

^{1}H-NMR δ 8.26 (d, *J* = 2.9 Hz, 1H), 7.71 (s, 1H), 6.47 (dd, *J* = 3.0, 1.5 Hz, 1H), 5.61 (dd, *J* = 7.8, 5.3 Hz, 1H), 4.55 (t, *J* = 7.8 Hz, 1H), 4.14 (dd, *J* = 9.1, 5.4 Hz, 1H), 1.57 (s, 3H), 1.48 (s, 3H).

(1*H*-pyrazol-1-yl)(tetrahydrofuran-2-yl)methanone **2c**

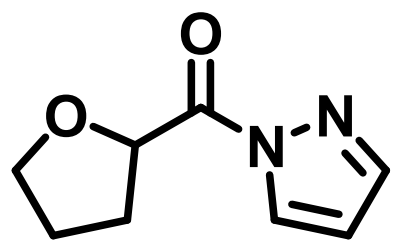

Prepared via the general procedure outlined in section 2.1, from tetrahydrofurfuryl alcohol **1c** (51 mg, 0.5 mmol) and pyrazole (102 mg, 1.5 mmol, 3 equiv). The crude mixture was analyzed by qNMR (55%). The product was isolated via extraction, as described in section 2.2. This afforded the product as a colorless oil (37 mg, 45%). The product was characterized by ^{1}H-NMR and the spectral data match the literature[1] values.

^{1}H-NMR (400 MHz, $CDCl_3$) δ 8.25 (dd, *J* = 2.9, 0.7 Hz, 1H), 7.71 (s, 1H), 6.44 (dd, *J* = 2.9, 1.5 Hz, 1H), 5.55 (dd, *J* = 8.7, 5.5 Hz, 1H), 4.12 (d, *J* = 7.0 Hz, 1H), 4.02 (q, *J* = 6.4 Hz, 1H), 2.55 – 2.45 (m, 1H), 2.12 – 1.94 (m, 3H).

1-(1*H*-pyrazol-1-yl)pentan-1-one **2d**

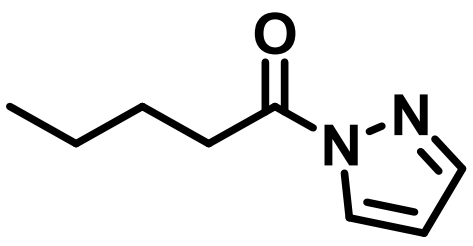

Prepared via the general procedure outlined in section 2.1, from 1-pentanol **1d** (44 mg, 0.5 mmol) and pyrazole (102 mg, 1.5 mmol, 3 equiv). The crude mixture was analyzed by qNMR (75%). The product was isolated via extraction, as described in section 2.2. This afforded the product as a colorless oil (51 mg, 68%). The product was characterized by ^{1}H-NMR and ^{13}C-NMR and the spectral data match the literature[2] values.

^{1}H-NMR (400 MHz, $CDCl_3$) δ 8.26 (dd, *J* = 2.9, 0.7 Hz, 1H), 7.70 (d, *J* = 1.0 Hz, 1H), 6.43 (dd, *J* = 2.9, 1.5 Hz, 1H), 3.14 (t, *J* = 7.5 Hz, 2H), 1.77 (p, *J* = 7.6 Hz, 2H), 1.49 – 1.41 (m, 2H), 0.96 (t, *J* = 7.4 Hz, 3H).

^{13}C-NMR (101 MHz, $CDCl_3$) δ 172.52, 143.96 (d, *J* = 10.1 Hz)*, 128.35, 109.56 (d, *J* = 12.0 Hz)*, 33.82, 26.59, 22.42 13.93.

**Note: The two doublets observed in the spectrum likely result from incomplete decoupling during the NMR measurement.*

Cyclohexyl(1*H*-pyrazol-1-yl)methanone **2e**

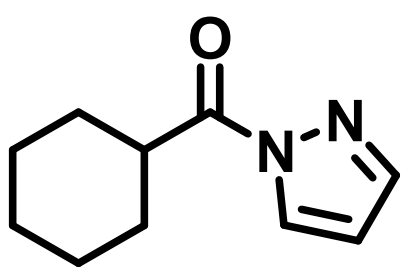

Prepared via the general procedure outlined in section 2.1, from cyclohexanemethanol **1e** (57 mg, 0.5 mmol) and pyrazole (102 mg, 1.5 mmol, 3 equiv). The crude mixture was analyzed by qNMR (83%). The

product was isolated via extraction, as described in section 2.2. This afforded the product as a colorless oil (74 mg, 83%). The product was characterized by ^{1}H-NMR and the spectral data match the literature[1] values.

^{1}H-NMR (400 MHz, $CDCl_3$) δ 8.24 (d, *J* = 2.8 Hz, 1H), 7.71 (d, *J* = 1.5 Hz, 1H), 6.42 (dd, *J* = 2.8, 1.5 Hz, 1H), 3.63 (tt, *J* = 11.6, 3.5 Hz, 1H), 1.99 (dddd, *J* = 13.6, 5.1, 3.6, 1.5 Hz, 2H), 1.83 (dt, *J* = 10.2, 3.3 Hz, 2H), 1.74 (dddt, *J* = 12.8, 5.1, 3.3, 1.5 Hz, 1H), 1.56 (qd, *J* = 12.3, 3.1 Hz, 2H), 1.41 (qt, *J* = 12.6, 3.2 Hz, 2H), 1.29 (tt, *J* = 12.5, 3.4 Hz, 1H).

(4-methoxyphenyl)(1*H*-pyrazol-1-yl)methanone **2f**

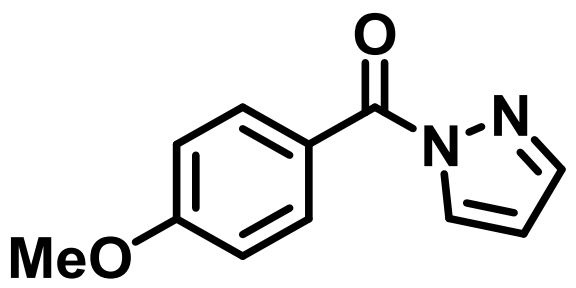

Prepared via the general procedure outlined in section 2.1, from 4-methoxybenzyl alcohol **1f** (69 mg, 0.5 mmol) and pyrazole (102 mg, 1.5 mmol, 3 equiv). The crude mixture was analyzed by qNMR (85%). The product was isolated via column chromatography, as described in section 2.3. This afforded the product as a colorless oil (80 mg, 79%). The product was characterized by ^{1}H-NMR and the spectral data match the literature[1] values.

^{1}H-NMR (400 MHz, $CDCl_3$) δ 8.43 (d, *J* = 2.7 Hz, 1H), 8.25 – 8.17 (m, 2H), 7.79 (t, *J* = 1.0 Hz, 1H), 7.02 – 6.96 (m, 2H), 6.50 (dd, *J* = 2.9, 1.5 Hz, 1H), 3.89 (s, 3H)

(1*H*-pyrazol-1-yl)-1,2:3,4-Di-*O*-isopropylidene-α-D-galactopyranose **2g**

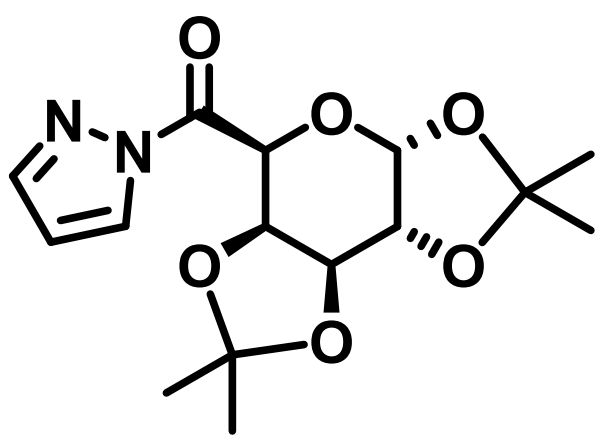

Prepared via the general procedure outlined in section 2.1, from 1,2:3,4-di-*O*-isopropylidene-α-D -galactopyranose **1g** (130 mg, 0.5 mmol) and pyrazole (102 mg, 1.5 mmol, 3 equiv). The crude mixture was analyzed by qNMR (88%). The product was isolated via extraction, as described in section 2.2. This afforded the product as a colorless oil (131 mg, 81%). The product was characterized by ^{1}H-NMR and the spectral data match the literature[1] values.

^{1}H-NMR (400 MHz, $CDCl_3$) δ 8.26 (d, *J* = 2.9 Hz, 1H), 7.69 (d, *J* = 1.5 Hz, 1H), 6.42 (dd, *J* = 2.9, 1.5 Hz, 1H), 5.73 (d, *J* = 5.1 Hz, 1H), 5.55 (d, *J* = 2.5 Hz, 1H), 4.92 (dd, *J* = 7.5, 2.5 Hz, 1H), 4.68 (dd, *J* = 7.5, 2.8 Hz, 1H), 4.44 (dd, *J* = 5.1, 2.8 Hz, 1H), 1.56 (s, 3H), 1.45 (s, 3H), 1.34 (s, 3H), 1.25 (s, 3H).

(1*H*-pyrazol-1-yl)-3,4,5-tris(benzyloxy)-6-methoxy-α-D-glucopyranoside **2h**

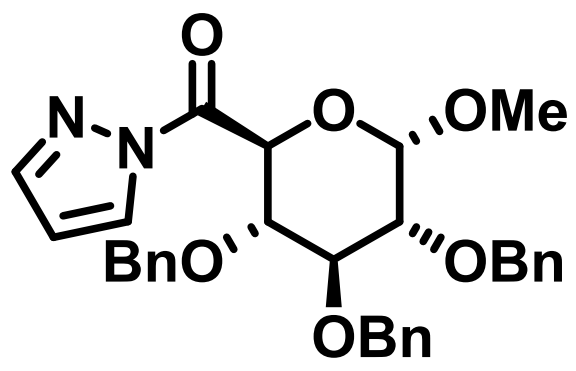

Prepared via the general procedure outlined in section 2.1, from 3,4,5-tris(benzyloxy)-6-methoxy-α-D-glucopyranoside **1h** (264 mg, 0.57 mmol), which was synthesized according to a known literature procedure[3], and pyrazole (116 mg, 1.7 mmol, 3 equiv). The crude mixture was analyzed by qNMR (82%). The product was isolated via extraction, as described in section 2.2. This afforded the product as a colorless oil (262 mg, 87%).

^{1}H-NMR (400 MHz, $CDCl_3$) δ 8.24 (d, *J* = 2.9 Hz, 1H), 7.75 (d, *J* = 1.4 Hz, 1H), 7.39 – 7.27 (m, 10H), 7.21 – 7.17 (m, 3H), 7.01 – 6.97 (m, 2H), 6.48 (dd, *J* = 2.9, 1.5 Hz, 1H), 5.58 (d, *J* = 9.9 Hz, 1H), 4.87 – 4.78 (m, 3H), 4.71 – 4.61 (m, 2H), 4.55 (d, *J* = 10.9 Hz, 1H), 4.15 (t, *J* = 9.3 Hz, 1H), 4.01 (dd, *J* = 9.9, 9.0 Hz, 1H), 3.63 (dd, *J* = 9.6, 3.5 Hz, 1H), 3.52 (s, 3H).

^{13}C-NMR (151 MHz, $CDCl_3$) δ 168.34, 144.87, 138.74, 138.11, 137.97, 128.96, 128.66 (C2), 128.56 (C2), 128.35 (C2), 128.34 (C2), 128.17, 128.11 (C2), 127.84 (C2), 127.81, 127.73, 110.71, 99.31, 81.69, 79.61, 79.40, 76.05, 75.34, 73.81, 67.47, 56.21.

HRMS (ESI^+) calculated for $C_{31}H_{32}N_2O_6Na^+$ ($[M+Na]^+$): 551.2153, found: 551.2145.

$[\alpha]^{20}$ = − 8 (c = 0.1 in chloroform)

(1*H*-pyrazol-1-yl)-2,3:4,5-Di-O-isopropylidene-β-D-fructopyranose **2i**

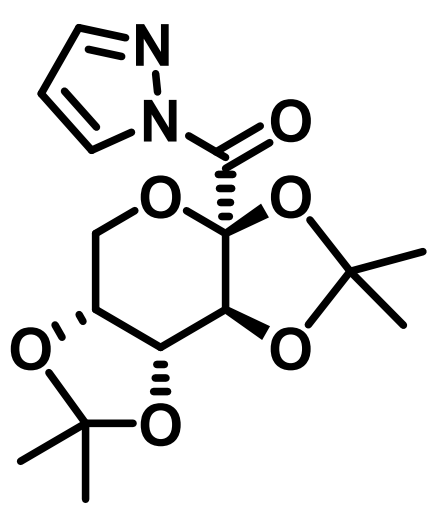

Prepared via the general procedure outlined in section 2.1, from 2,3:4,5-di-*O*-isopropylidene-β-D-fructopyranose **1i** (130 mg, 0.5 mmol) and pyrazole (102 mg, 1.5 mmol, 3 equiv). The crude mixture was analyzed by qNMR (70%). The product was isolated via extraction, as described in section 2.2. This afforded the product as a colorless oil (93 mg, 58%)

^{1}H-NMR (400 MHz, $CDCl_3$) δ 8.40 (d, *J* = 2.9 Hz, 1H), 7.85 (d, *J* = 1.4 Hz, 1H), 6.42 (dd, *J* = 2.9, 1.4 Hz, 1H), 5.29 (d, *J* = 2.4 Hz, 1H), 4.71 (dd, *J* = 8.0, 2.4 Hz, 1H), 4.30 (dd, *J* = 8.1, 1.7 Hz, 1H), 4.04 (dd, *J* = 12.9, 1.9 Hz, 1H), 3.91 (dd, *J* = 12.9, 0.9 Hz, 1H), 1.59 (s, 3H), 1.36 (s, 3H), 1.34 (s, 6H).

^{13}C-NMR (151 MHz, $CDCl_3$) δ 163.10, 145.36, 132.90, 110.45, 109.50, 109.32, 101.12, 71.17, 70.04, 70.00, 61.59, 26.20, 26.00, 24.72, 24.25.

HRMS (ESI^+) calculated for $C_{15}H_{20}N_2O_6Na^+$ ($[M+Na]^+$): 347.1214, found: 347.1210.

$[\alpha]^{20}$ = − 28 (c = 0.1 in chloroform)

((3a*R*,5*S*,6*R*,6a*R*)-6-(benzyloxy)-2,2-dimethyltetrahydrofuro[2,3-*d*][1,3]dioxol-5-yl)(1*H*-pyrazol-1-yl)methanone **2j**

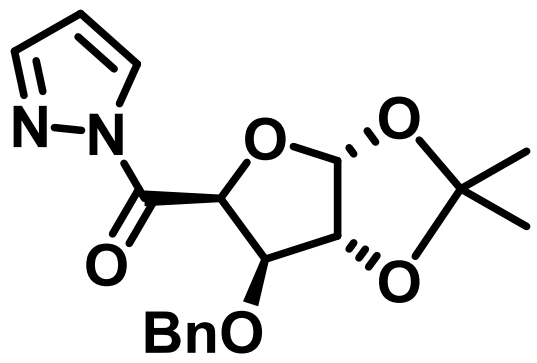

Prepared via the general procedure outlined in section 2.1, from ((3a*R*,5*R*,6*S*,6a*R*)-6-(benzyloxy)-2,2-dimethyltetrahydrofuro[2,3-d][1,3]dioxol-5-yl)methanol **1j** (140 mg, 0.5 mmol), which was synthesized according to the literature[4,5], and pyrazole (102 mg, 1.5 mmol, 3 equiv). The crude mixture was analyzed by qNMR (95%). The product was isolated via extraction, as described in section 2.2. This afforded the product as a colorless oil (169 mg, 98%)

^{1}H-NMR (400 MHz, $CDCl_3$) δ 8.29 (d, *J* = 2.9 Hz, 1H), 7.57 (s, 1H), 7.24 – 7.07 (m, 3H), 6.95 – 6.91 (m, 2H), 6.44 (dd, *J* = 3.0, 1.5 Hz, 1H), 6.20 (d, *J* = 3.7 Hz, 1H), 5.83 (d, *J* = 4.1 Hz, 1H), 4.73 (d, *J* = 4.1 Hz, 1H), 4.66 (d, *J* = 3.7 Hz, 1H), 4.50 (d, *J* = 12.0 Hz, 1H), 4.24 (d, *J* = 12.0 Hz, 1H), 1.54 (s, 3H), 1.37 (s, 3H).

^{13}C-NMR (151 MHz, $CDCl_3$) δ 165.88, 144.30, 136.76, 128.67, 128.46 (C2), 127.93, 127.74 (C2), 112.76, 109.78, 105.97, 83.75, 82.48, 80.94, 72.13, 27.35, 26.64.

HRMS (ESI+) calculated for $C_{18}H_{20}N_2O_5H^+$ ($[M+H]^+$): 345.1445, found: 345.1443.

[α]^{20} = − 100 (c = 0.1 in chloroform)

((2*S*,2'*S*,3*S*,3'*S*,4*S*,4'*S*,5*R*,5'*R*,6*R*,6'*R*)-oxybis(3,4,5-tris(benzyloxy)tetrahydro-2*H*-pyran-6,2-diyl))bis((1*H*-pyrazol-1-yl)methanone) **2k**

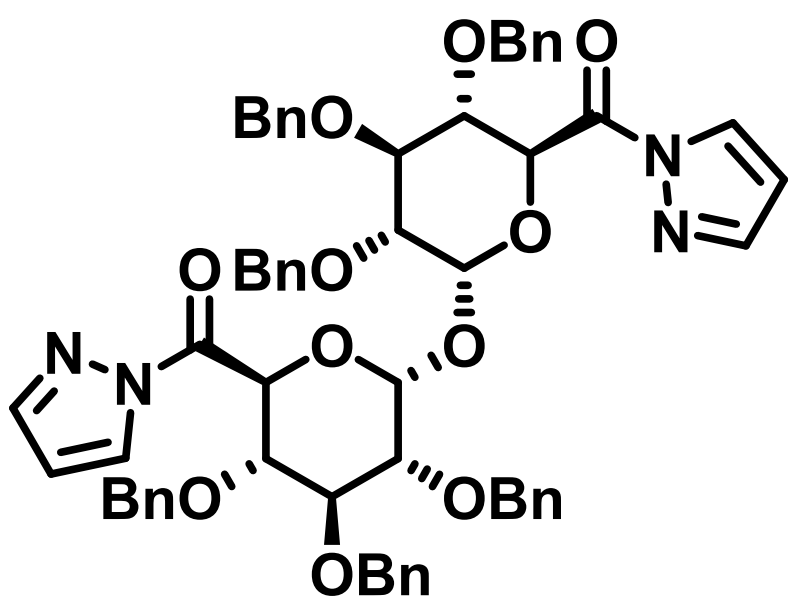

Prepared via the general procedure outlined in section 2.1 from ((2*R*,2'*R*,3*R*,3'*R*,4*S*,4'*S*,5*R*,5'*R*,6*R*,6'*R*)-oxybis(3,4,5-tris(benzyloxy)tetrahydro-2*H*-pyran-6,2-diyl))dimethanol **1k** (442 mg, 0.5 mmol), which was synthesized according to the literature[6], and pyrazole (204 mg, 3 mmol, 6 equiv) using twice the number of equivalents for all other added reagents and passing 12 F/mol of charge. The crude mixture was analyzed by qNMR (75%). The product was isolated via extraction, as described in section 2.2. This afforded the product as a white solid (405 mg, 80%), with 95% purity. An aliquot was taken and further purified by column chromatography (SiO_2, 10 – 40% EtOAc in pentane) to afford the pure N-acyl pyrazole, as a white solid.

^{1}H-NMR (400 MHz, $CDCl_3$) δ 8.21 (d, *J* = 2.9 Hz, 1H), 7.63 (s, 1H), 7.38 – 7.05 (m, 13H), 7.03 – 6.96 (m, 2H), 6.42 (dd, *J* = 3.0, 1.4 Hz, 1H), 5.84 (d, *J* = 9.8 Hz, 1H), 5.65 (d, *J* = 3.4 Hz, 1H), 5.04 (d, *J* = 10.6 Hz, 1H), 5.01 (d, *J* = 11.1 Hz, 1H), 4.90 (d, *J* = 10.9 Hz, 1H), 4.85 (d, *J* = 11.0 Hz, 1H), 4.73 (d, *J* = 11.7 Hz, 1H), 4.58 (d, *J* = 11.0 Hz, 1H), 4.33 (t, *J* = 9.3 Hz, 1H), 4.07 (t, *J* = 9.5 Hz, 1H), 3.76 (dd, *J* = 9.5, 3.4 Hz, 1H).

^{13}C-NMR (101 MHz, $CDCl_3$) δ 168.23, 144.79, 138.88, 138.27, 138.15, 129.11, 128.49 (C2), 128.41 (C2), 128.30 (C2), 128.08 (C2), 128.02 (C2), 127.89 (C2), 127.69, 127.65, 127.63, 110.63, 93.38, 81.27, 79.45, 78.86, 75.87, 75.33, 73.27, 68.69.

HRMS (ESI+) calculated for $C_{60}H_{58}N_4O_{11}NH_4^+$ ($[M+NH_4]^+$): 1028.4440, found: 1028.4417.

[α]^{20} = 8 (c = 0.1 in chloroform)

Mp 38 °C

(3,5-dimethyl-1*H*-pyrazol-1-yl)-1,2:3,4-Di-*O*-isopropylidene-α-D-galactopyranose **2l**

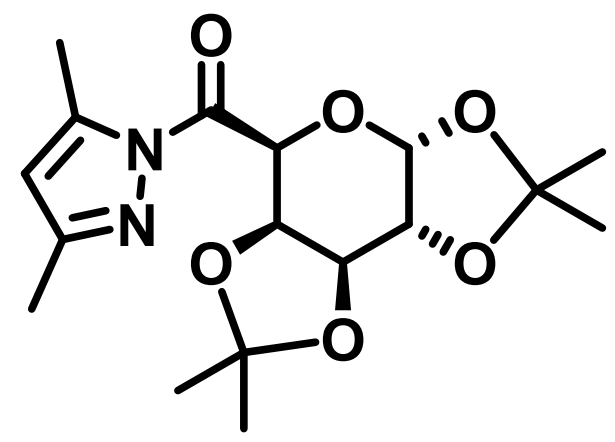

Prepared via the general procedure outlined in section 2.1, from 1,2:3,4-di-*O*-isopropylidene-α-D-galactopyranose **1g** (130 mg, 0.5 mmol) and 3,5-dimethylpyrazole (144 mg, 1.5 mmol, 3 equiv). The crude mixture was analyzed by qNMR (88%). The product was isolated via extraction, as described in section 2.2. This afforded the product as a colorless oil (142 mg, 81%).

^{1}H-NMR (400 MHz, $CDCl_3$) δ 5.94 (d, *J* = 1.2 Hz, 1H), 5.75 (d, *J* = 5.1 Hz, 1H), 5.54 (d, *J* = 2.5 Hz, 1H), 4.98 (dd, *J* = 7.5, 2.5 Hz, 1H), 4.69 (dd, *J* = 7.5, 2.8 Hz, 1H), 4.45 (dd, *J* = 5.1, 2.8 Hz, 1H), 2.55 (s, 3H), 2.21 (s, 3H), 1.58 (s, 3H), 1.49 (s, 3H), 1.36 (s, 3H), 1.30 (s, 3H).

^{13}C-NMR (151 MHz, $CDCl_3$) δ 167.02, 152.57, 144.62, 111.03, 110.51, 109.33, 96.88, 72.71, 71.48, 70.55, 69.39, 26.14 (2C), 25.24, 25.19, 14.32, 14.05.

HRMS (ESI^+) calculated for $C_{17}H_{24}N_2O_6H^+$ ($[M+H]^+$): 353.1707, found: 353.1703.

[α]^{20} = − 90 (c = 0.1 in chloroform)

(1*H*-benzo[d]imidazol-1-yl)-1,2:3,4-Di-*O*-isopropylidene-α-D-galactopyranose **2m**

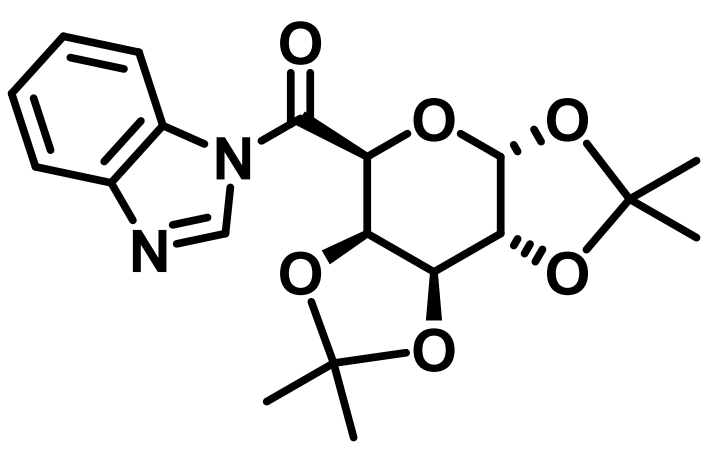

Prepared via the general procedure outlined in section 2.1, from 1,2:3,4-di-*O*-isopropylidene-α-D-galactopyranose **1g** (130 mg, 0.5 mmol) and benzimidazole (177 mg, 1.5 mmol, 3 equiv). The crude mixture was analyzed by qNMR (80%). The product was isolated via extraction, as described section 2.2. This afforded the product as a white foamy solid (149 mg, 80%).

¹H-NMR (400 MHz, $CDCl_3$) δ 9.00 (s, 1H), 8.32 – 8.27 (m, 1H), 7.80 – 7.74 (m, 1H), 7.44 – 7.36 (m, 2H), 5.76 (d, *J* = 4.9 Hz, 1H), 4.97 (d, *J* = 1.7 Hz, 1H), 4.75 (qd, *J* = 7.9, 2.0 Hz, 2H), 4.46 (dd, *J* = 4.9, 2.2 Hz, 2H), 1.60 (s, 3H), 1.48 (s, 3H), 1.39 (s, 3H), 1.34 (s, 3H).

¹³C-NMR (151 MHz, $CDCl_3$) δ 167.17, 143.54, 143.00, 132.12, 125.80, 125.43, 120.35, 116.14, 110.30, 109.70, 96.70, 71.92, 71.79, 70.75, 70.71, 26.19, 25.82, 24.87, 23.88.

HRMS (ESI^+) calculated for $C_{19}H_{22}N_2O_6H^+$ ($[M+H]^+$): 375.1551, found: 375.1544.

$[\alpha]^{20}$ = −120 (c = 0.1 in chloroform)

mp 58 °C

10.2 Functionalization reactions

Piperidin-1-yl-1,2:3,4-Di-*O*-isopropylidene-α-D-galactopyranose **3g**

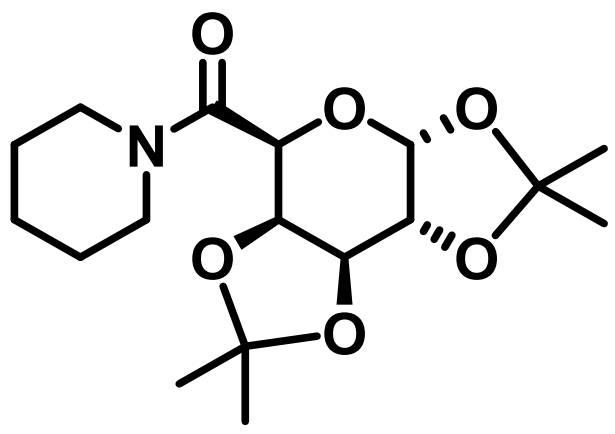

Product **3g** was prepared via the general procedure outlined in section 3 from 1,2:3,4-di-*O*-isopropylidene-α-D-galactopyranose **1g** (130 mg, 0.5 mmol) and piperidine (128, 1.5 mmol, 3 equiv). This afforded the product as a colorless oil (150 mg, 87%).

¹H-NMR (400 MHz, $CDCl_3$) δ 5.60 (d, *J* = 5.0 Hz, 1H), 4.63 – 4.50 (m, 3H), 4.29 (dd, *J* = 5.0, 1.9 Hz, 1H), 3.75 – 3.40 (m, 4H), 1.63 – 1.52 (m, 6H), 1.51 (s, 3H), 1.44 (s, 3H), 1.32 (s, 3H), 1.31 (s, 3H).

¹³C-NMR (151 MHz, $CDCl_3$) δ 166.25, 109.55, 108.88, 96.81, 71.94, 70.88, 70.86, 69.84, 46.60, 44.26, 26.88, 26.16, 25.90, 25.78, 24.95, 24.82, 24.10.

HRMS (ESI^+) calculated for $C_{17}H_{27}NO_6H^+$ ($[M+H]^+$): 342.1911, found: 342.1910.

$[\alpha]^{20}$ = − 48 (c = 0.1 in chloroform)

(3a*R*,5*S*,5a*R*,8a*S*,8b*R*)-N-benzyl-2,2,7,7-tetramethyltetrahydro-5*H*-bis([1,3]dioxolo)[4,5-*b*:4',5'-*d*]pyran-5-carboxamide **4g**

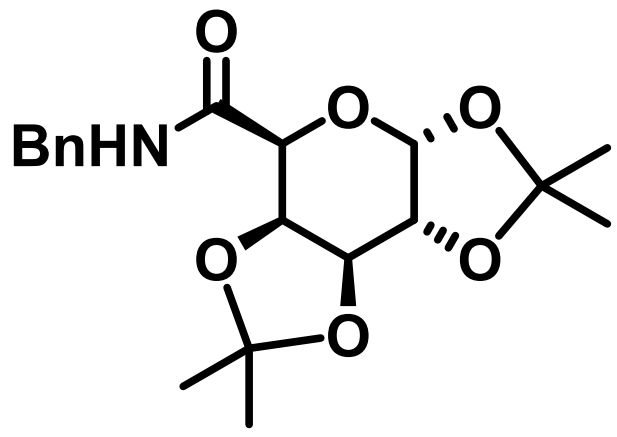

Product **4g** was prepared via the general procedure outlined in section 3 from 1,2:3,4-di-*O*-isopropylidene-α-D-galactopyranose **1g** (130 mg, 0.5 mmol) and benzylamine (161 mg, 1.5 mmol, 3 equiv). This afforded the product as a white solid (143 mg, 79%).

^{1}H-NMR (400 MHz, $CDCl_3$) δ 7.31 (m, 4H), 7.29 – 7.24 (m, 1H), 6.89 – 6.83 (m, 1H), 5.56 (d, *J* = 4.9 Hz, 1H), 4.78 (dd, *J* = 8.14, 7.33 Hz, 1H), 4.75 (dd, *J* = 5.77, 2.15 Hz, 1H), 4.67 (dd, *J* = 7.9, 2.5 Hz, 1H), 4.38 – 4.34 (m, 2H), 4.28 (dd, *J* = 15.0, 4.7 Hz, 1H), 1.52 (s, 3H), 1.41 (s, 3H), 1.36 (s, 3H), 1.33 (s, 3H).

^{13}C-NMR (151 MHz, $CDCl_3$) δ 168.47, 138.07, 128.62 (2C), 127.71 (2C), 127.44, 109.54, 109.37, 96.36, 71.78, 70.85, 70.53, 68.95, 42.85, 26.09, 26.02, 24.94, 24.32.

HRMS (ESI$^+$) calculated for $C_{19}H_{25}NO_6Na^+$ ([M+Na]$^+$): 386.1574, found: 386.1569.

[α]^{20} = − 88 (c = 0.1 in chloroform)

(3a*R*,5*S*,5a*R*,8a*S*,8b*R*)-2,2,7,7-tetramethyltetrahydro-5*H*-bis([1,3]dioxolo)[4,5-*b*:4',5'-*d*]pyran-5-carboxamide**5g**

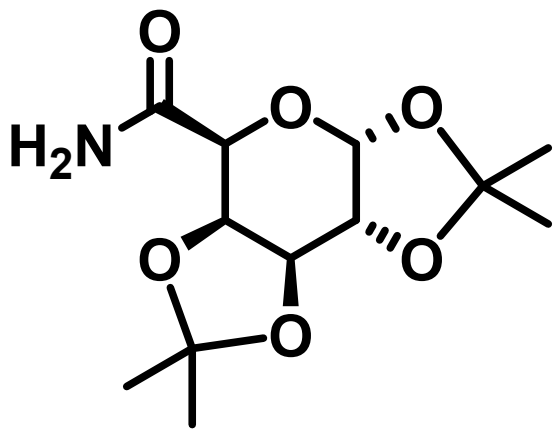

Product **5g** was prepared via the general procedure outlined in section 3 from 1,2:3,4-di-*O*-isopropylidene-α-D-galactopyranose **1g** (130 mg, 0.5 mmol) and 0.5 M NH_3 in dioxane (3 mL, 1.5 mmol, 3 equiv). This afforded the product as a white solid (122 mg, 89%), with 95% purity. An aliquot was taken and further purified by column chromatography (SiO_2, 0 – 10% MeOH in DCM) to afford the pure N-acyl pyrazole, as a white solid.

^{1}H-NMR (600 MHz, $CDCl_3$) δ 6.51 (s, 1H), 5.77 (s, 1H), 5.59 (d, *J* = 4.9 Hz, 1H), 4.71 – 4.64 (m, 2H), 4.36 (dd, *J* = 4.9, 2.2 Hz, 1H), 4.31 (d, *J* = 1.8 Hz, 1H), 1.52 (s, 3H), 1.44 (s, 3H), 1.34 (d, *J* = 2.6 Hz, 6H).

^{13}C-NMR (151 MHz, $CDCl_3$) δ 171.19, 109.61, 109.31, 96.39, 71.51, 70.82, 70.52, 68.88, 26.06, 25.98, 24.90, 24.28.

HRMS (ESI$^+$) calculated for $C_{12}H_{19}NO_6H^+$ ([M+H]$^+$): 274.1285, found: 274.1287.

[α]^{20} = − 92 (c = 0.1 in chloroform)

Mp 141 °C

methyl (3a*R*,5*S*,5a*R*,8a*S*,8b*R*)-2,2,7,7-tetramethyltetrahydro-5*H*-bis([1,3]dioxolo)[4,5-*b*:4',5'-*d*]pyran-5-carboxylate **6g**

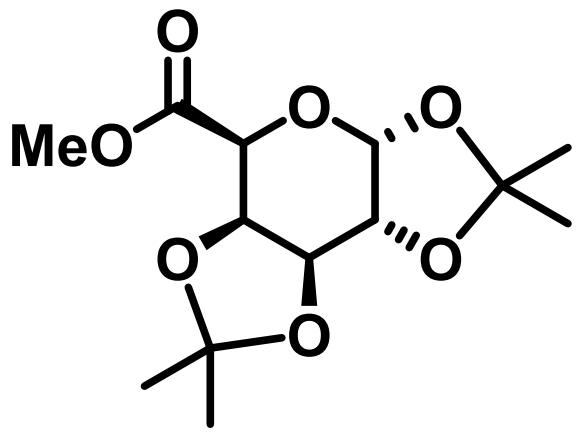

Product **6g** was prepared via the general procedure outlined in section 4 from 1,2:3,4-di-*O*-isopropylidene-α-D-galactopyranose **1g** (130 mg, 0.5 mmol) and methanol. This afforded the product as a colorless oil (117 mg, 81%).

^{1}H-NMR (600 MHz, $CDCl_3$) δ 5.66 (d, *J* = 5.0 Hz, 1H), 4.66 (dd, *J* = 5.5, 2.3 Hz, 1H), 4.58 (dd, *J* = 5.9, 2.1 Hz, 1H), 4.45 (d, *J* = 2.3 Hz, 1H), 4.38 (dd, *J* = 4.2, 2.1 Hz, 1H), 3.82 (s, 3H), 1.53 (s, 3H), 1.45 (s, 3H), 1.34 (s, 6H).

^{13}C-NMR (151 MHz, $CDCl_3$) δ 168.89, 110.21, 109.19, 96.71, 72.23, 70.83, 70.44, 68.67, 52.53, 26.12, 26.02, 24.94, 24.77.

HRMS (ESI^+) calculated for $C_{13}H_{20}O_7Na^+$ ($[M+Na]^+$): 311.1101, found: 311.1101.

[α]^{20} = − 88 (c = 0.1 in chloroform)

S-phenyl-(3a*R*,5*S*,5a*R*,8a*S*,8b*R*)-2,2,7,7-tetramethyltetrahydro-5*H*-bis([1,3]dioxolo)[4,5-*b*:4',5'-*d*]pyran-5-carbothioate **7g**

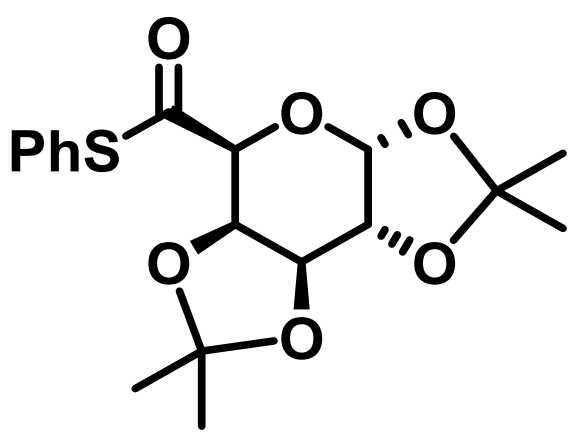

Product **7g** was prepared via the general procedure outlined in section 5 from 1,2:3,4-di-*O*-isopropylidene-α-D-galactopyranose **1g** (130 mg, 0.5 mmol) and thiophenol (306 µL, 3 mmol, 6 equiv). This afforded the product as a colorless oil (148 mg, 81%). The product was characterized by ^{1}H-NMR and the spectral data match the literature[7] values.

^{1}H-NMR (400 MHz, $CDCl_3$) δ 7.46 – 7.37 (m, 5H), 5.73 (d, *J* = 4.8 Hz, 1H), 4.68 (dd, *J* = 7.7, 2.6 Hz, 1H), 4.63 (dd, *J* = 7.7, 2.2 Hz, 1H), 4.48 (d, *J* = 2.1 Hz, 1H), 4.42 (dd, *J* = 4.9, 2.5 Hz, 1H), 1.55 (s, 3H), 1.53 (s, 3H), 1.37 (s, 3H), 1.34 (s, 3H).

10.3 Electrochemical oxidation of a thioglycoside

(1*H*-pyrazol-1-yl)((2*S*,3*R*,4*S*,5*R*,6*R*)-3,4,5-tris(benzyloxy)-6-(phenylthio)tetrahydro-2*H*-pyran-2-yl)methanone **2n**

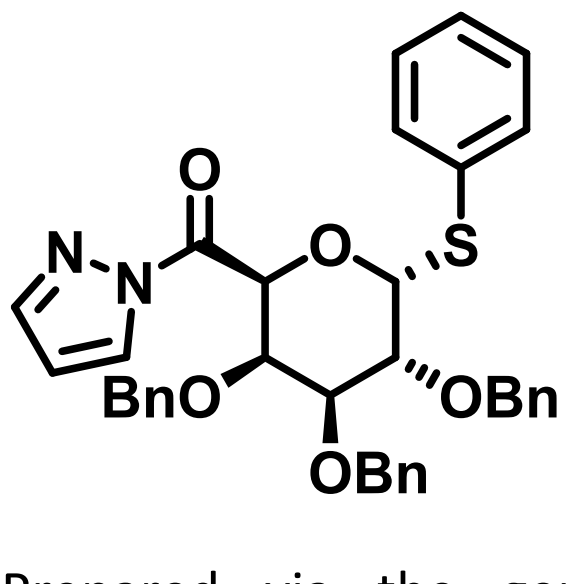

Prepared via the general procedure outlined in section 2.1, from ((2*R*,3*S*,4*S*,5*R*,6*R*)-3,4,5-tris(benzyloxy)-6-(phenylthio)tetrahydro-2*H*-pyran-2-yl)methanol **1n** (271 mg, 0.5 mmol), which was synthesized according to the literature[8,9], and pyrazole (102 mg, 1.5 mmol, 3 equiv). The crude mixture was analyzed by qNMR (47%) and TLC-MS.

TLC-MS (APCI) calculated for $C_{36}H_{34}N_2O_5SH^+$ ([M+H]$^+$): 607.2, found: 607.6.

Piperidin-1-yl((2*S*,3*R*,4*S*,5*R*,6*R*)-3,4,5-tris(benzyloxy)-6-(phenylthio)tetrahydro-2*H*-pyran-2-yl)methanone **3n**

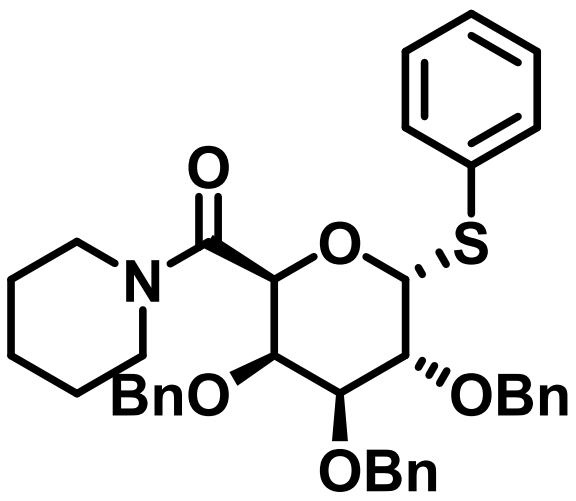

In a 10 mL vial, ((2*R*,3*S*,4*S*,5*R*,6*R*)-3,4,5-tris(benzyloxy)-6-(phenylthio)tetrahydro-2*H*-pyran-2-yl)methanol **1n** (271 mg, 0.5 mmol), pyrazole (102 mg, 1.5 mmol, 3 equiv), 2.6-lutidine (262 µL, 2.25 mmol, 4.5 equiv), and ACT (21 mg, 20 mol%) were added in an 8 mL solution of 0.1 M tetrabutylammonium hexafluorophosphate ($TBAPF_6$) in MeCN. The mixture was stirred for 10 min before the vial was capped and equipped with the electrodes. Constant current electrolysis (5 mA, j = 1.1 mA/cm^2) was conducted at room temperature, with stirring at 1000 rpm and 6 F/mol of charge was passed. The reaction was stopped after 6 F/mol of charge had passed, or the limit of 1.5 V (vs Ag/Ag$^+$) was exceeded. At the end of the reaction, the electrodes were removed and rinsed with MeCN.

To the crude reaction mixture, the piperidine (148 µL, 1.5 mmol, 3 equiv) was added and the reaction stirred at room temperature until TLC showed complete conversion (30% EtOAc in pentane). The crude reaction mixture was concentrated *in vacuo*. The residue was purified by column chromatography (SiO_2, 10 – 40% EtOAc in pentane) to afford the amide product as a white solid, with 95% purity (146.3 mg, 47%).

^{1}H-NMR (400 MHz, $CDCl_3$) δ 7.59 – 7.54 (m, 2H), 7.42 – 7.21 (m, 18H), 4.93 – 4.88 (m, 3H), 4.86 (d, J = 10.3 Hz, 1H), 4.80 (d, J = 10.4 Hz, 1H), 4.74 (d, J = 10.3 Hz, 1H), 4.70 (d, J = 9.6 Hz, 1H), 4.14 (t, J = 9.3 Hz, 1H), 4.02 (d, J = 9.4 Hz, 1H), 3.74 (d, J = 9.0 Hz, 1H), 3.71 – 3.60 (m, 1H), 3.53 (d, J = 9.4 Hz, 1H), 3.50 – 3.44 (m, 1H), 3.18 (dt, J = 7.0, 4.2 Hz, 2H), 1.57 – 1.49 (m, 4H), 1.39 – 1.31 (m, 2H).

^{13}C-NMR (101 MHz, $CDCl_3$) δ 164.99, 138.70, 138.51, 138.08, 133.24, 132.91 (C2), 129.34, 129.17 (C2), 128.61 (C2), 128.58 (C2), 128.42 (C2), 128.23 (C2), 128.06 (C2), 127.93 (C2), 127.86, 127.76, 127.73, 88.71, 86.63, 80.47, 78.85, 76.04, 75.65, 75.50, 75.21, 46.70, 43.50, 26.56, 25.58, 24.59.

HRMS (ESI$^+$) calculated for $C_{38}H_{41}NO_5SH^+$ ([M+H]$^+$): 624.2778, found: 624.2776.

10.4 Evaluation of racemization at the α-chiral center

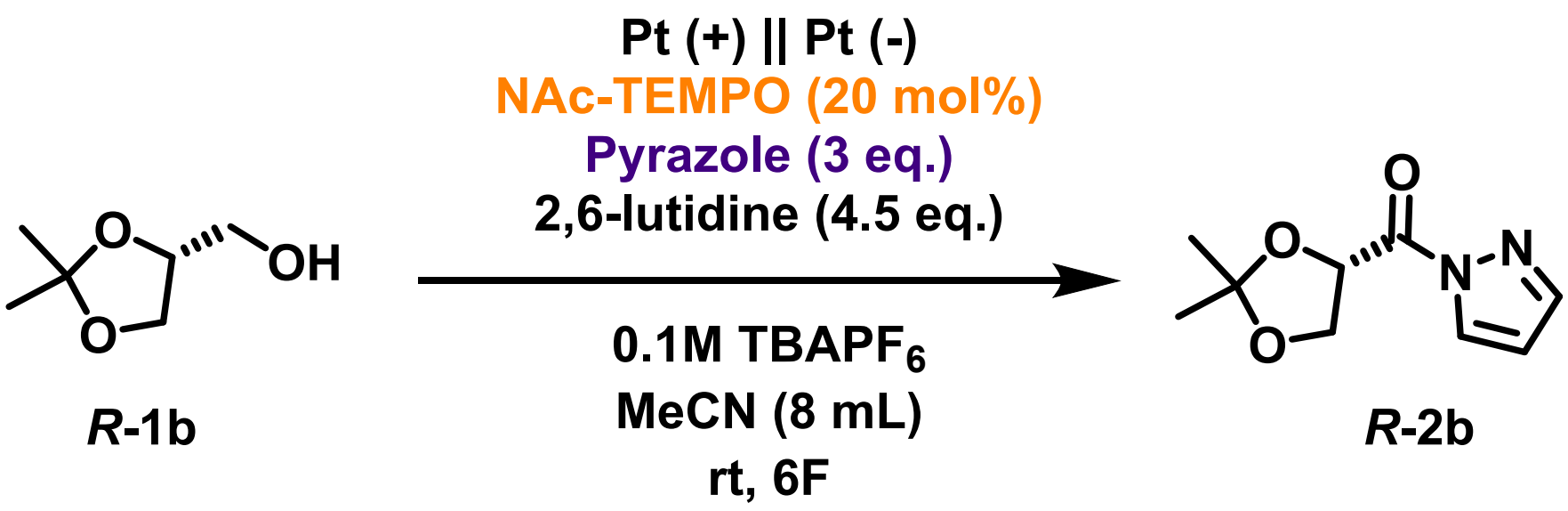

In a 10 mL vial, (±)-2,2-dimethyl-1,3-dioxolane-4-methanol ***rac*-1b** or (*R*)-2,2-dimethyl-1,3-dioxolane-4-methanol ***R*-1b** (66 mg, 0.5 mmol), pyrazole (102 mg, 1.5 mmol, 3 equiv), 2.6-lutidine (262 µL, 2.25 mmol, 4.5 equiv), and ACT (21 mg, 20 mol%) were added in an 8 mL solution of 0.1 M tetrabutylammonium hexafluorophosphate ($TBAPF_6$) in MeCN. The mixture was stirred for 10 min before the vial was capped and equipped with the electrodes. Constant current electrolysis (5 mA, j = 1.1 mA/cm^2) was conducted at room temperature, with stirring at 1000 rpm and 6 F/mol of charge was passed. The reaction was stopped after 6 F/mol of charge had passed, or the limit of 1.5 V (vs Ag/Ag^+) was exceeded. At the end of the reaction, the electrodes were removed and rinsed with MeCN.

The crude reaction mixture was concentrated *in vacuo*. Et_2O (~ 5 mL) was added, causing precipitation of the electrolyte, $TBAPF_6$. The mixture was filtered through a coarse-porosity fritted glass funnel. The solids were washed thoroughly with Et_2O (~ 25 mL). An aliquot containing 1mg substrate was taken and concentrated *in vacuo*. The residue was dissolved in 99 : 1 n-heptane : *i*-PrOH solution (~ 2 mL), the obtained solution was filtered through a 0.2 µm PTFE syringe filter and used for chiral HPLC without further purification.

The chiral HPLC analysis of ***rac*-2b** and ***R*-2b** was performed using a Shimadzu LC-20 series with a photodiode array detector, Chiralpak AD column, n-heptane : i-PrOH = 95 : 5 as mobile phase, a flow rate of 1 mL/min and UV detection at 240 nm. The results are presented in **Figure S41** & **Figure S42**.

11. Analysis data

11.1 ^1H and ^{13}C-NMR spectra of the isolated compounds

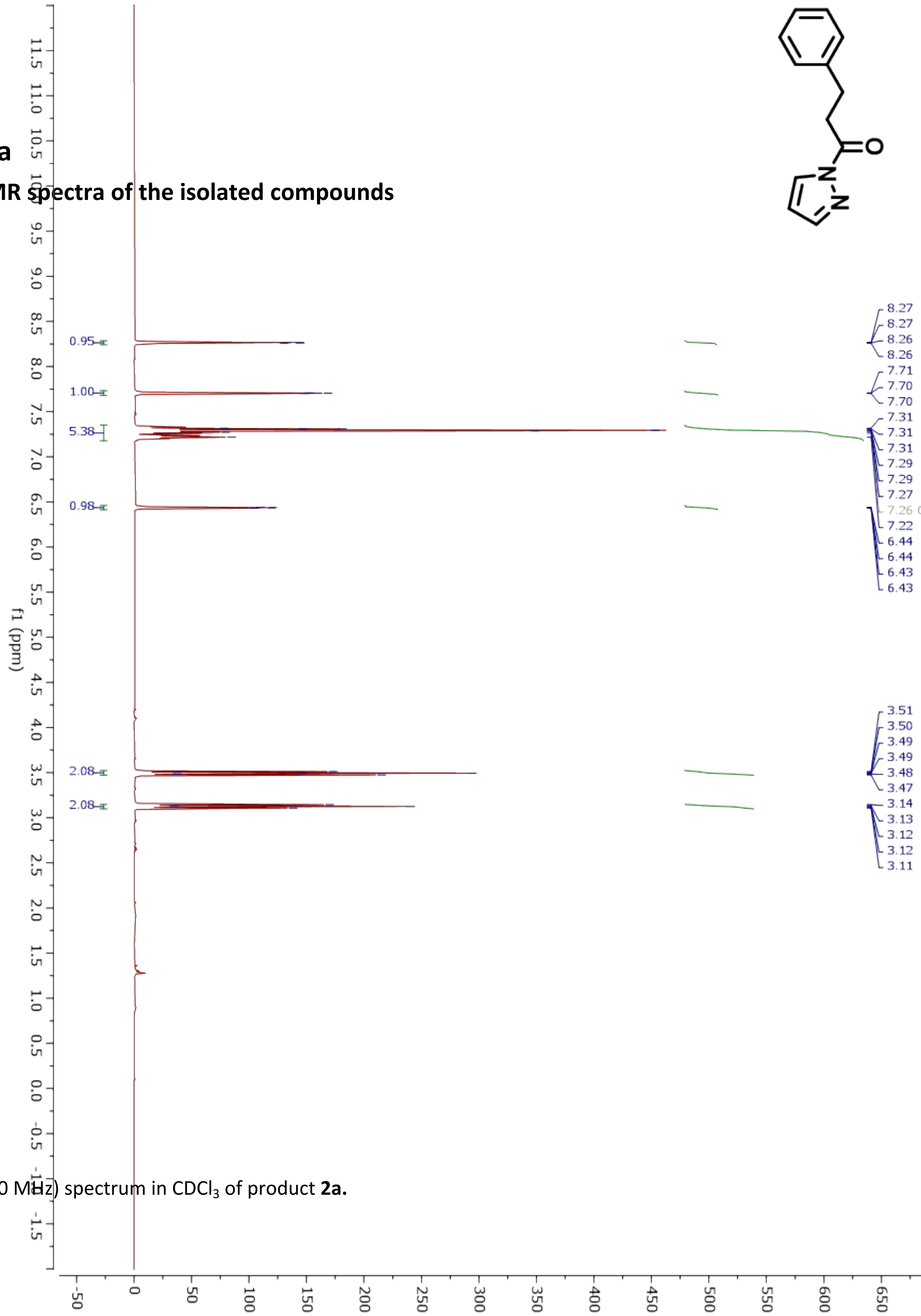

Figure S6: ^1H-NMR (400 MHz) spectrum in $CDCl_3$ of product **2a.**

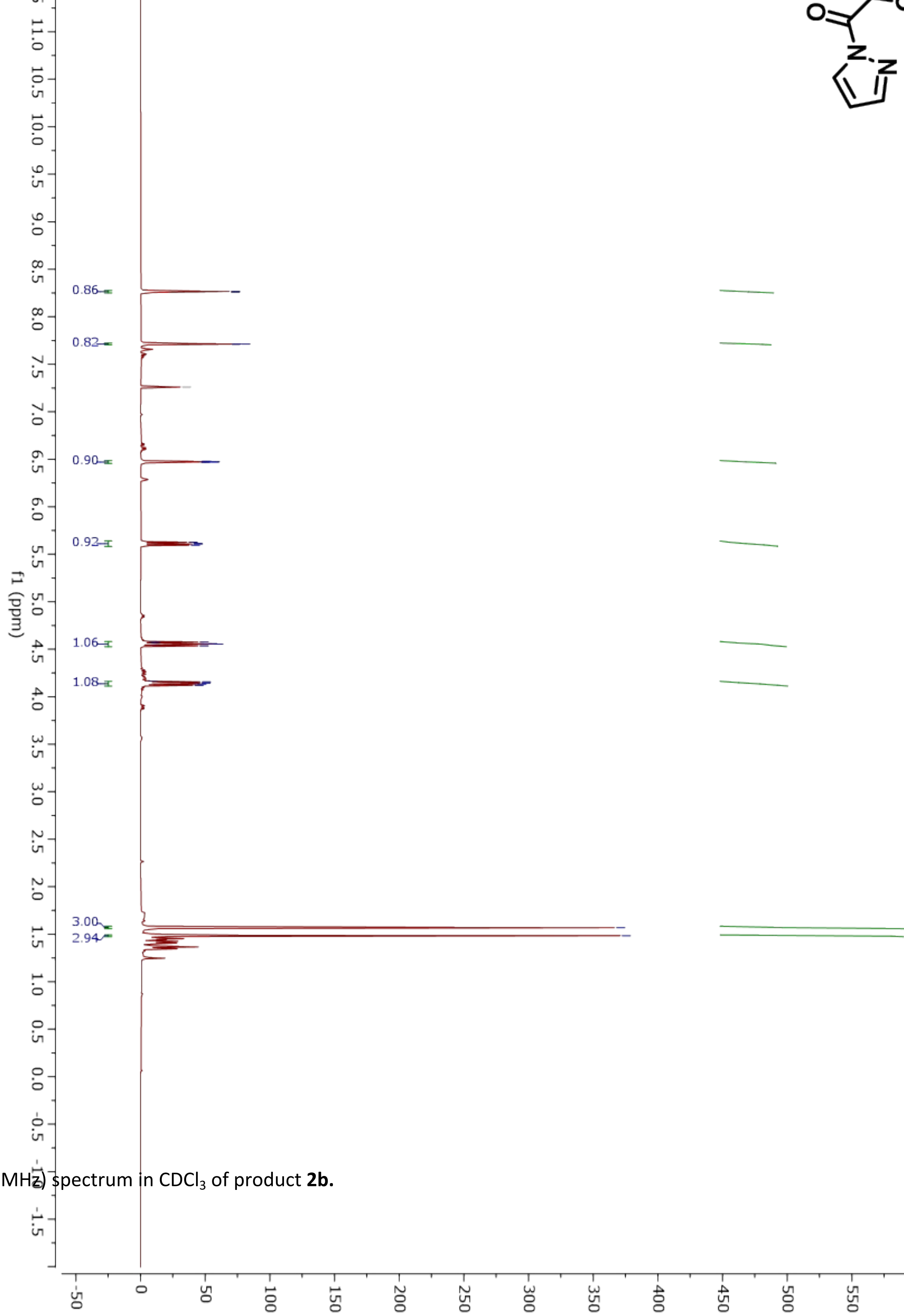

Figure S7: ^{1}H-NMR (400 MHz) spectrum in $CDCl_3$ of product **2b.**

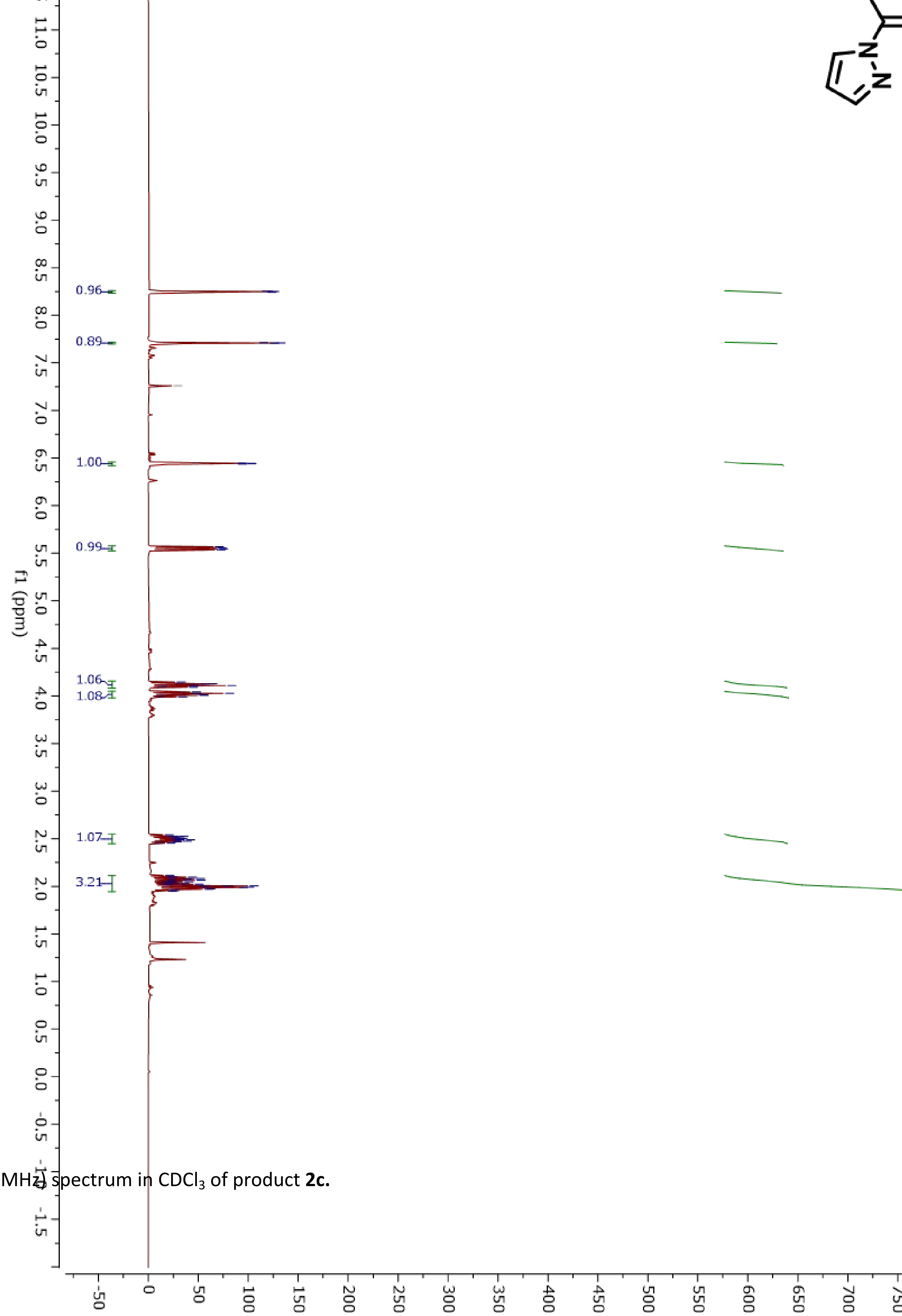

Figure S8: ^{1}H-NMR (400 MHz) spectrum in $CDCl_3$ of product **2c.**

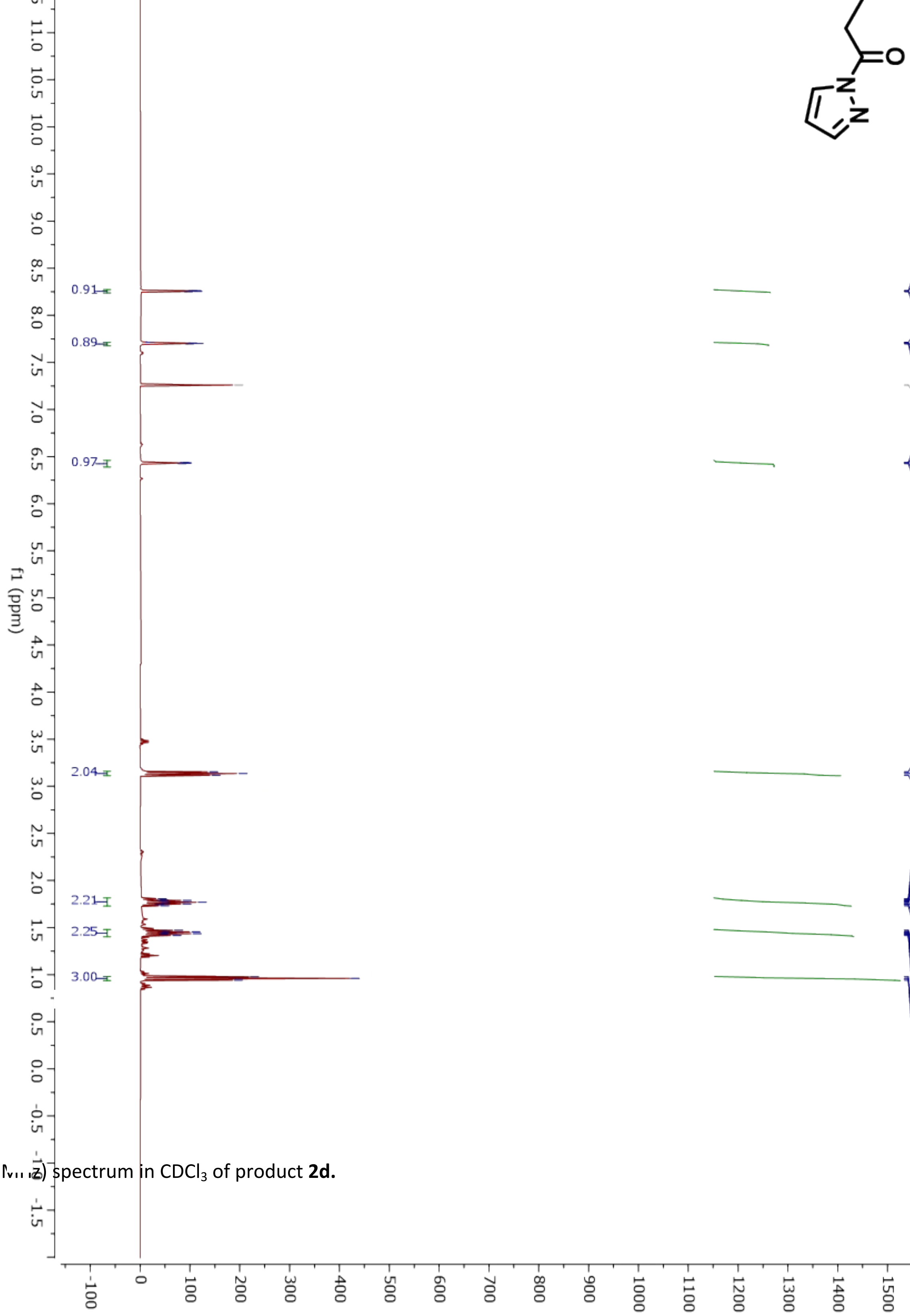

Figure S9: ^{1}H-NMR (400 MHz) spectrum in $CDCl_3$ of product **2d.**

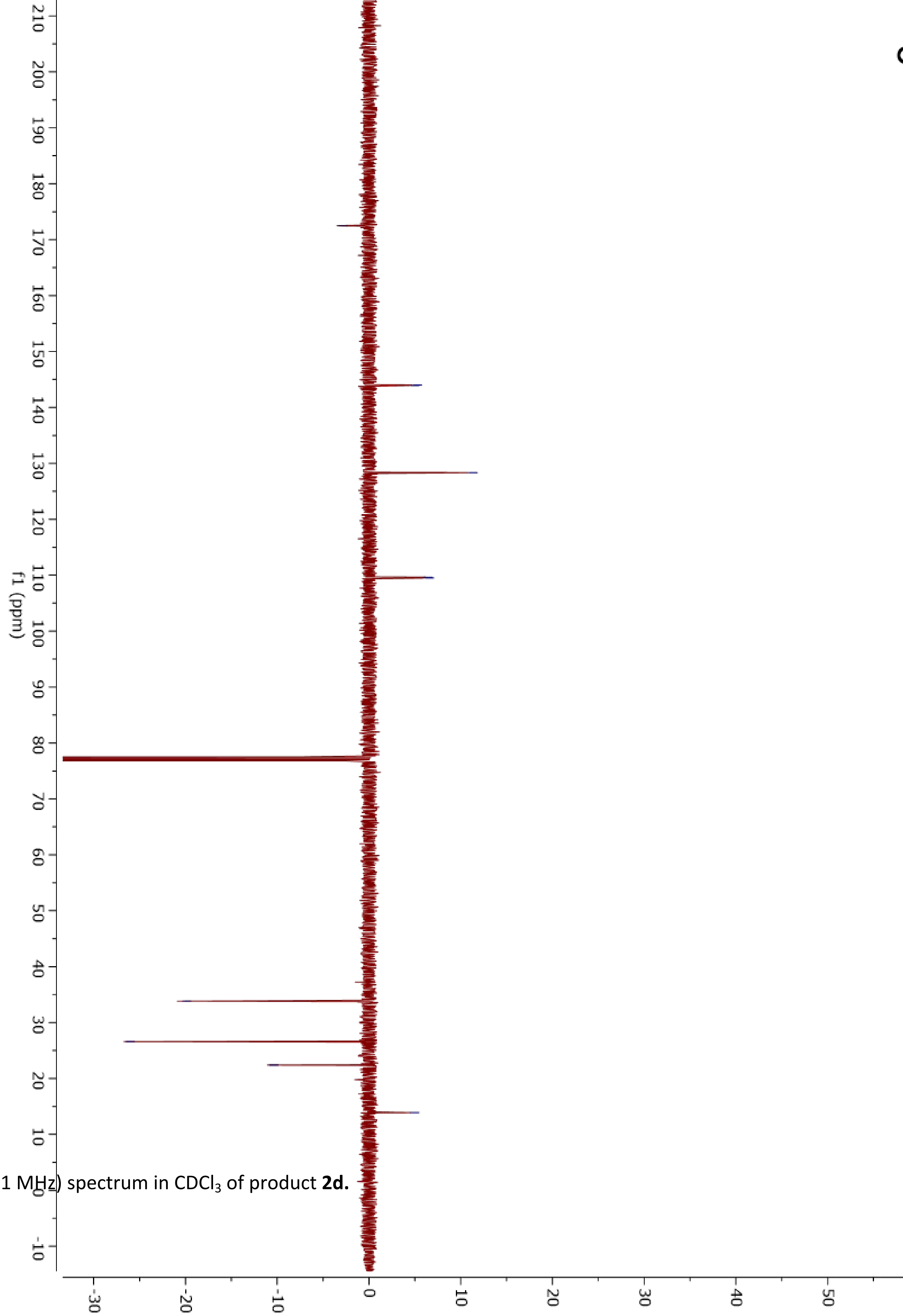

Figure S10: ^{13}C-NMR (101 MHz) spectrum in $CDCl_3$ of product **2d.**

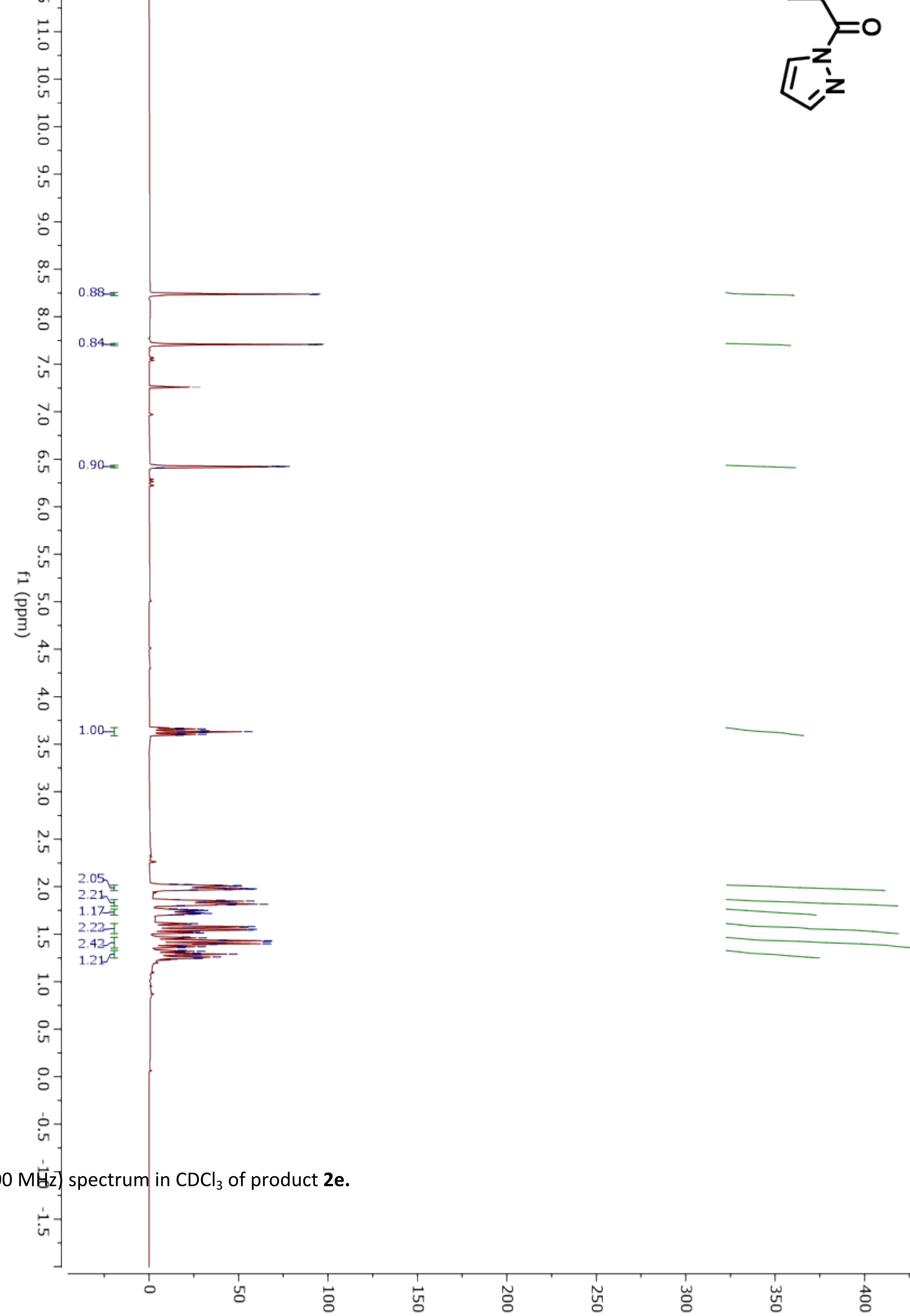

Figure S11: ^{1}H-NMR (400 MHz) spectrum in $CDCl_3$ of product **2e.**

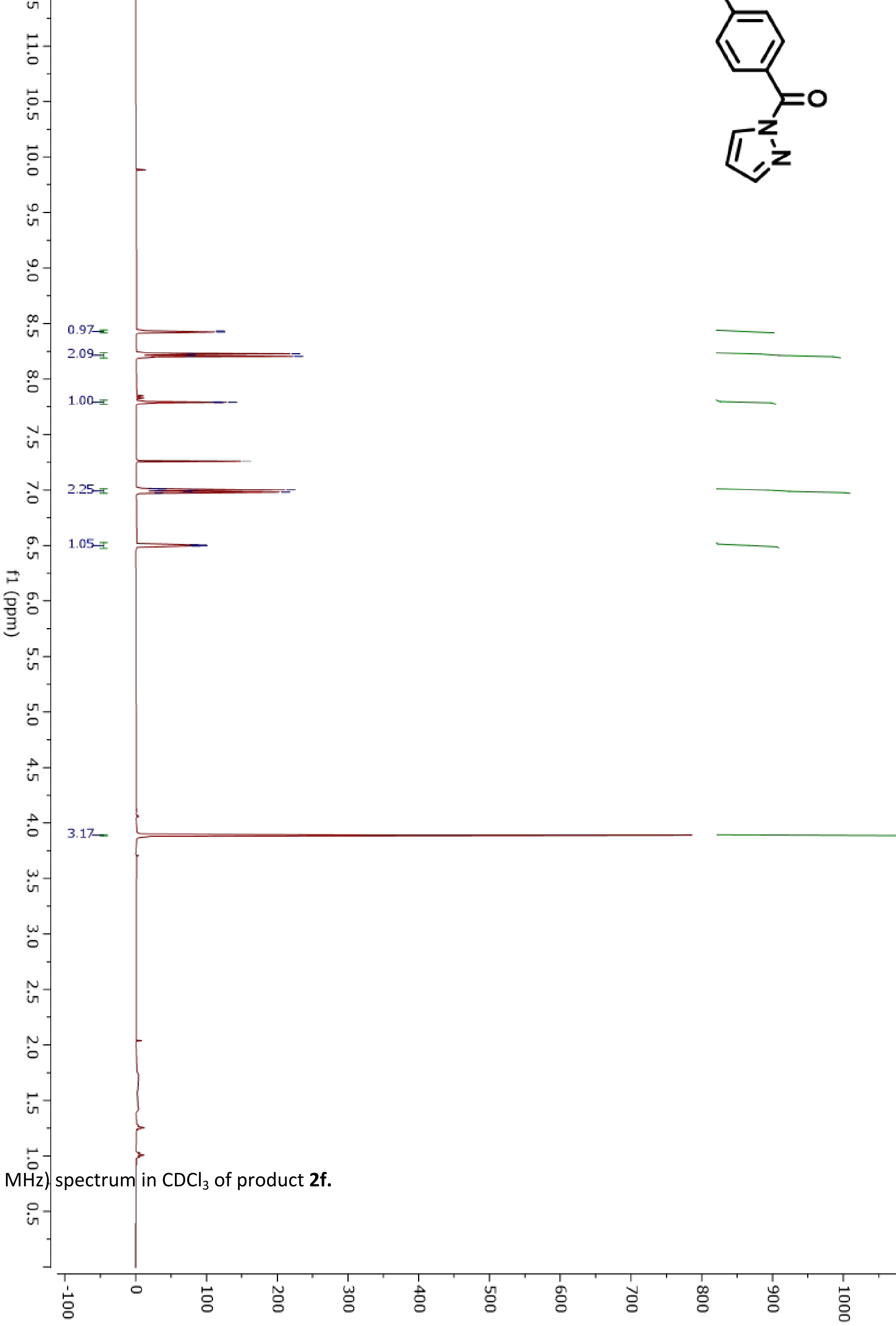

Figure S12: ^{1}H-NMR (400 MHz) spectrum in $CDCl_3$ of product **2f.**

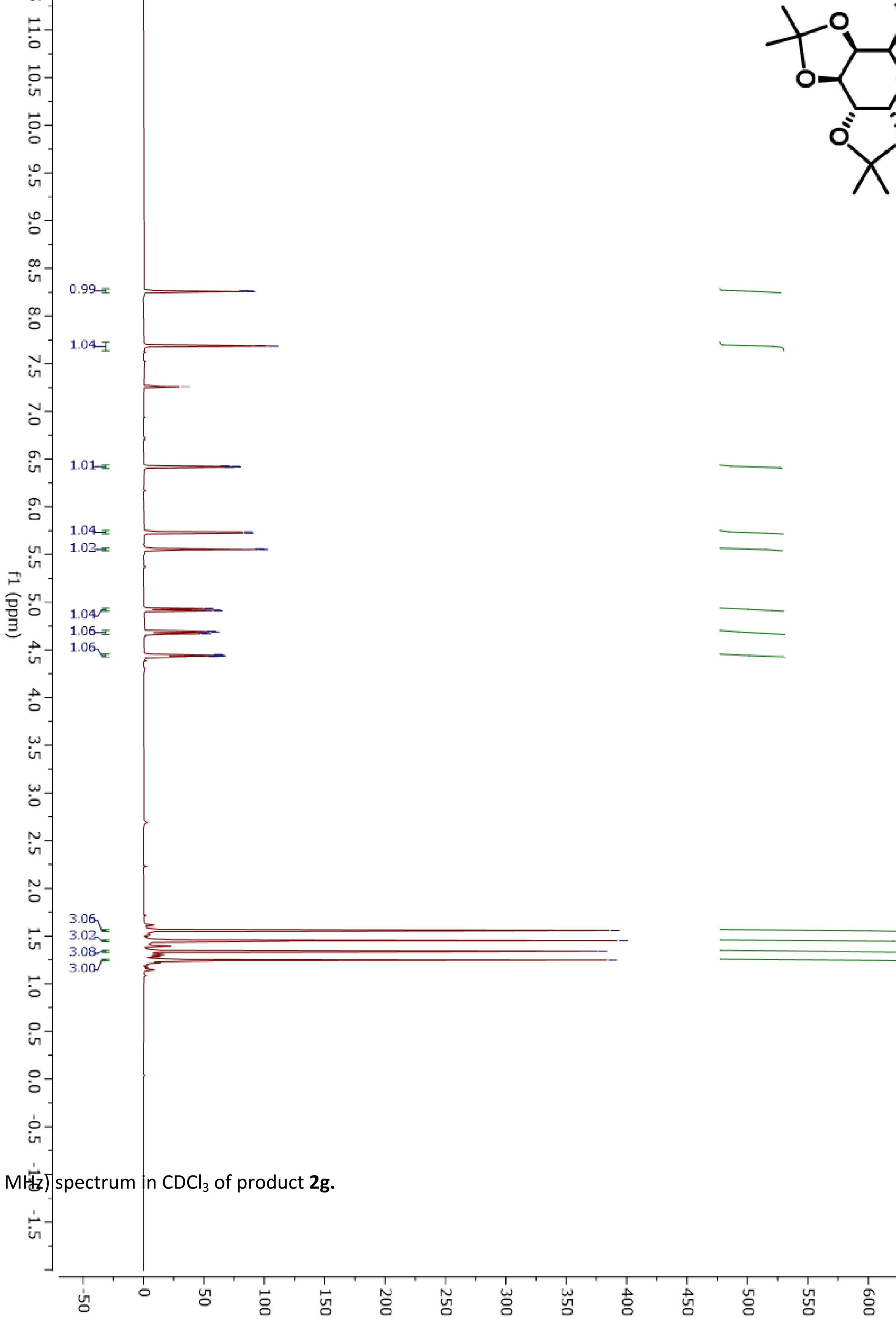

Figure S13: ^{1}H-NMR (400 MHz) spectrum in $CDCl_3$ of product **2g.**

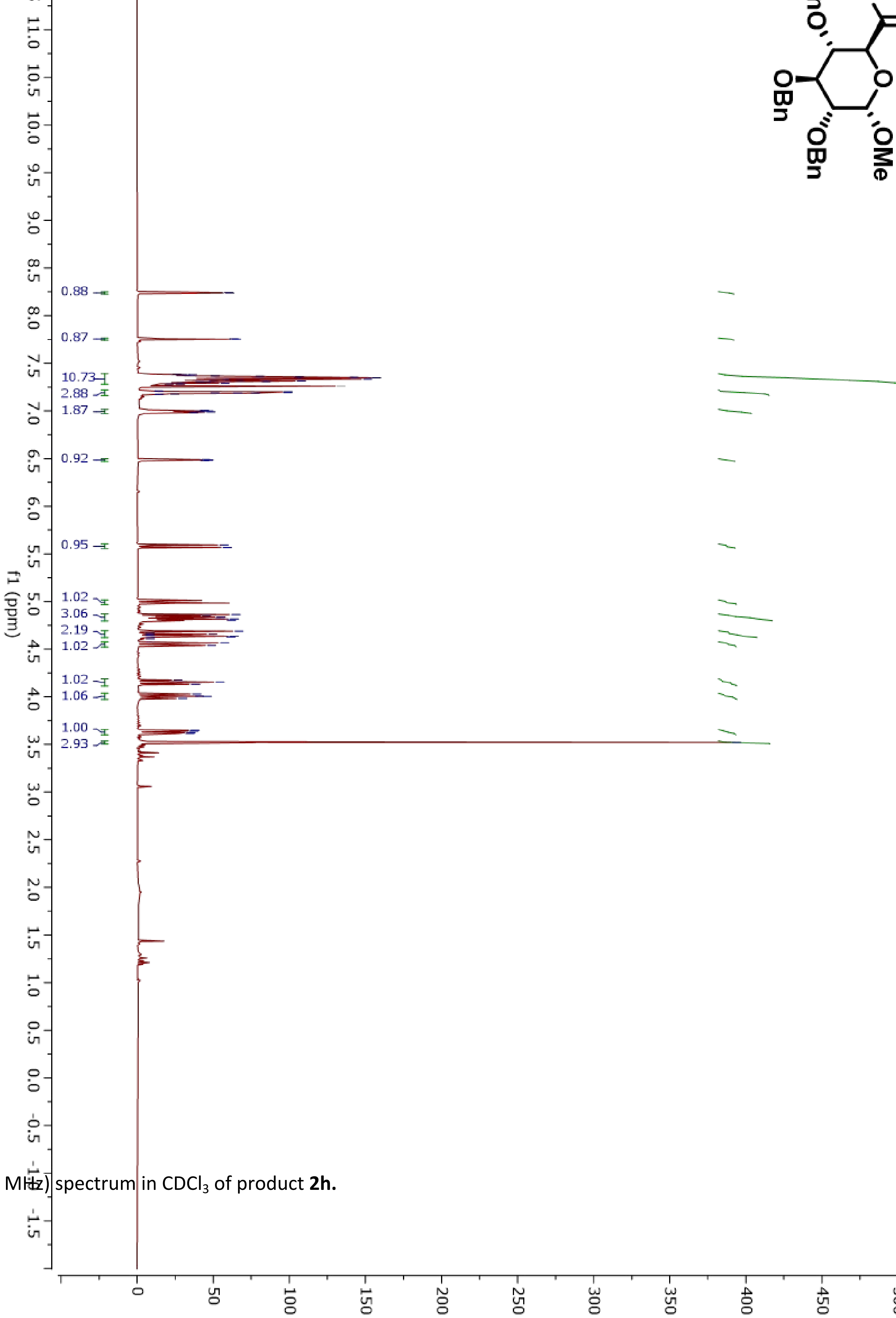

Figure S14: ^{1}H-NMR (400 MHz) spectrum in $CDCl_3$ of product **2h.**

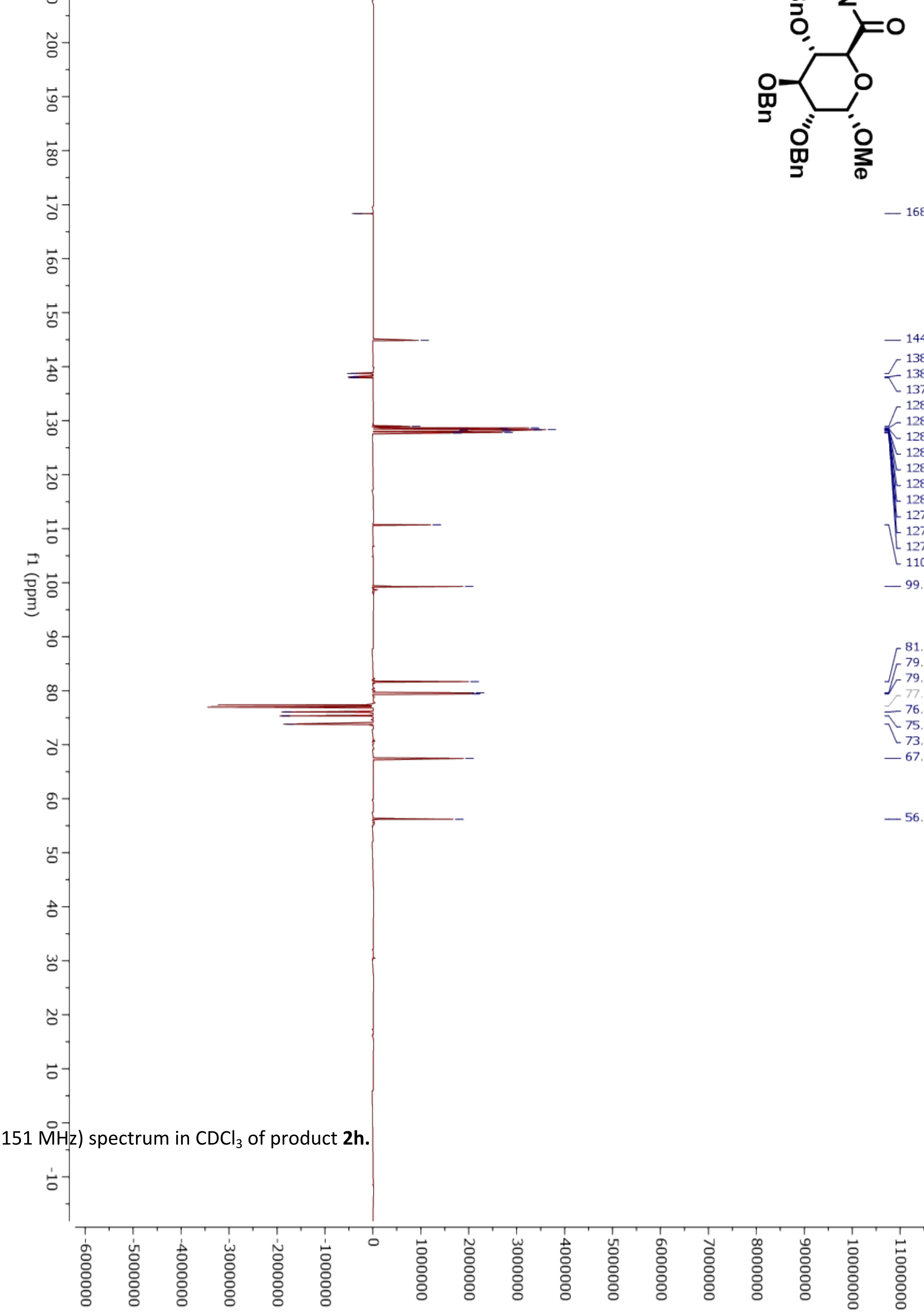

Figure S15: ^{13}C-NMR (151 MHz) spectrum in $CDCl_3$ of product **2h.**

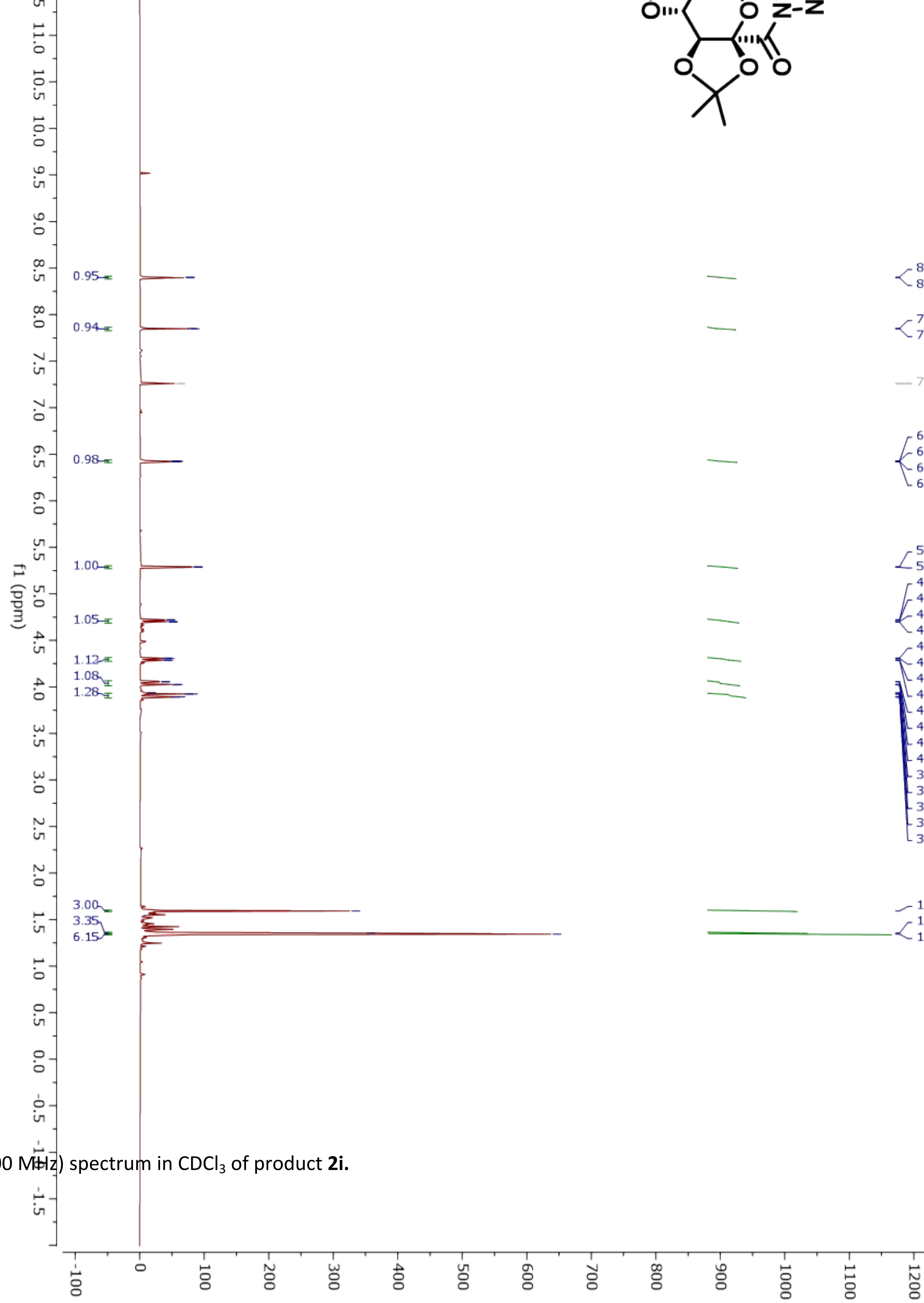

Figure S16: ^{1}H-NMR (400 MHz) spectrum in $CDCl_3$ of product **2i.**

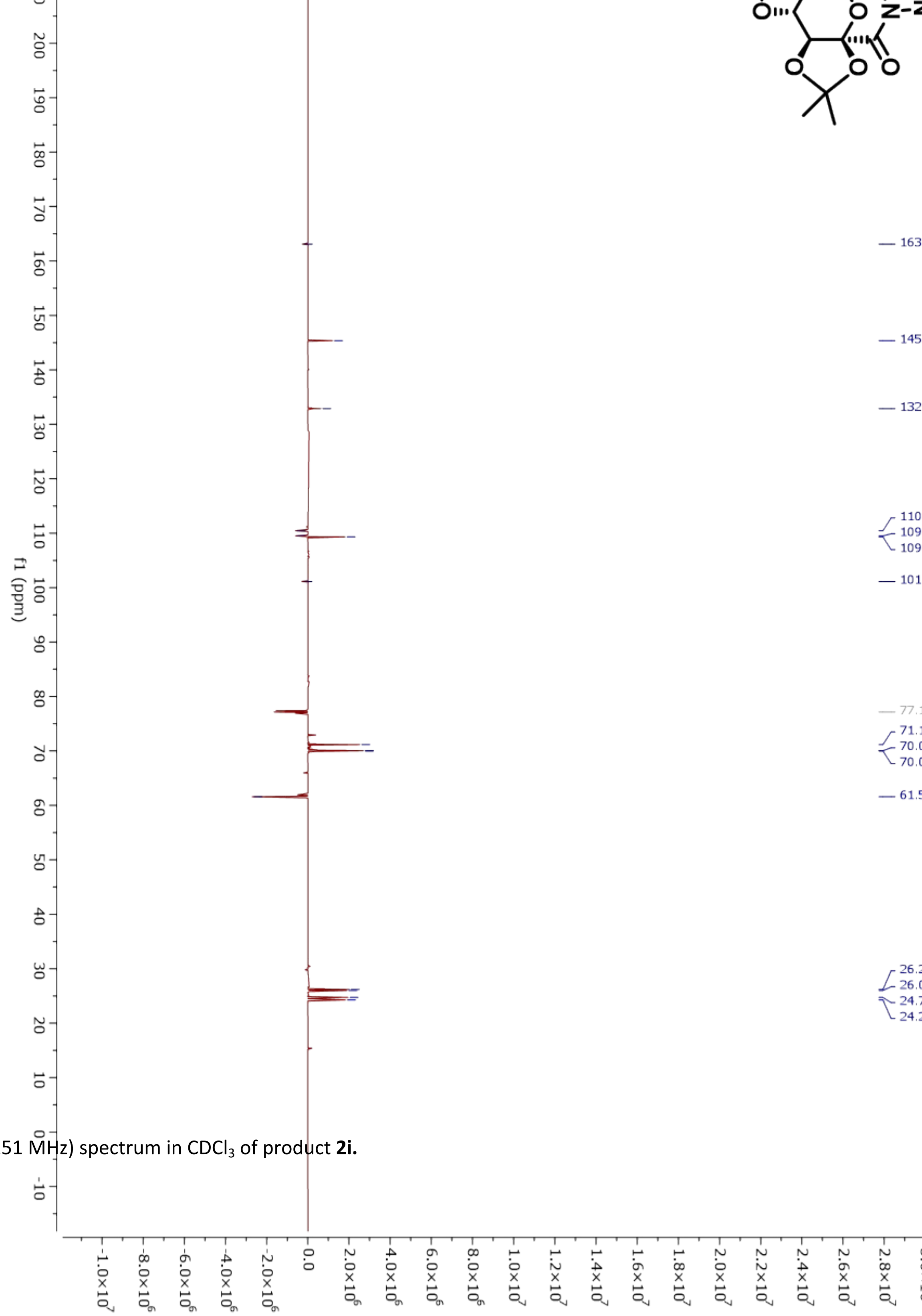

Figure S17: ^{13}C-NMR (151 MHz) spectrum in $CDCl_3$ of product **2i.**

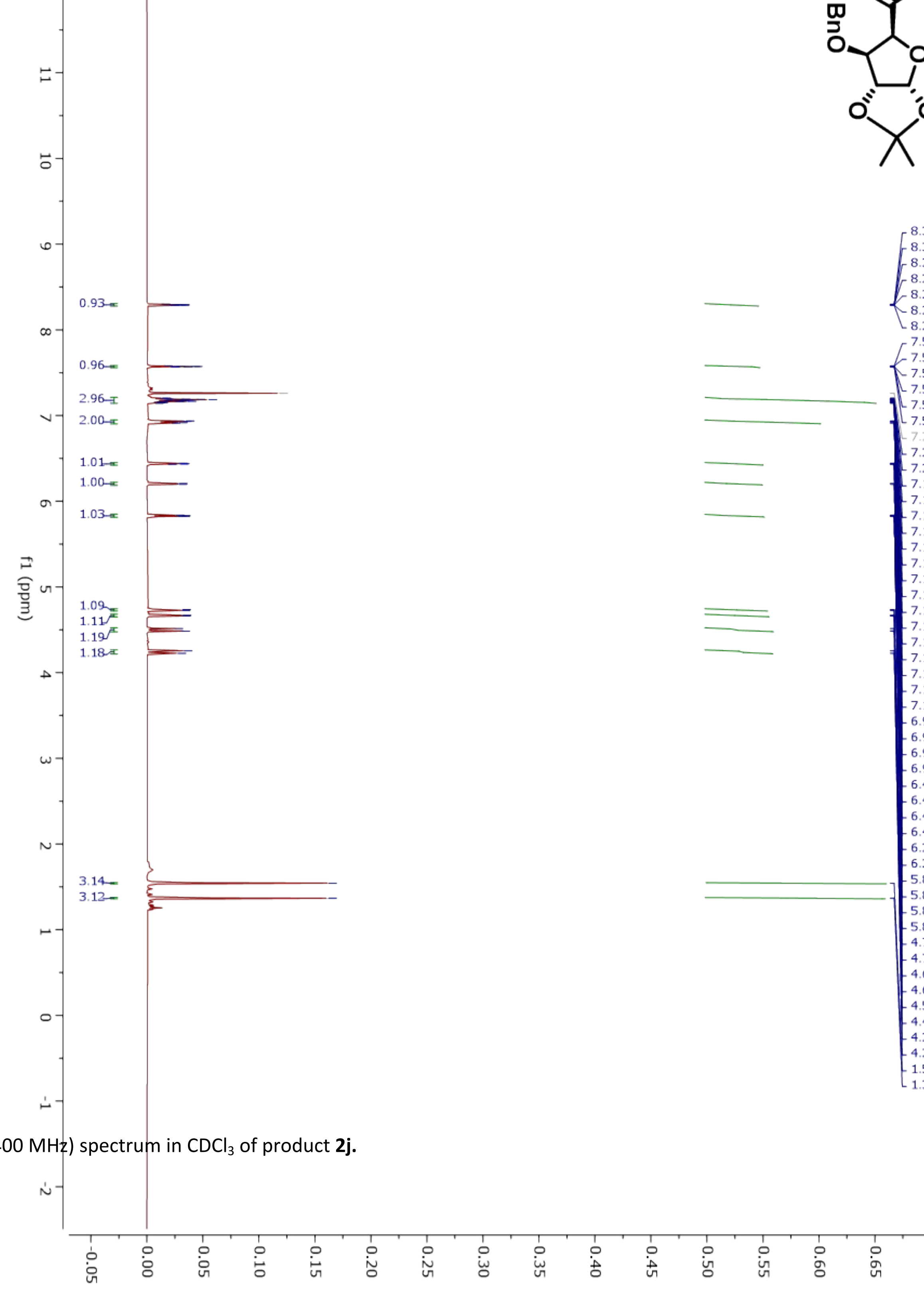

Figure S18: ^{1}H-NMR (400 MHz) spectrum in $CDCl_3$ of product **2j.**

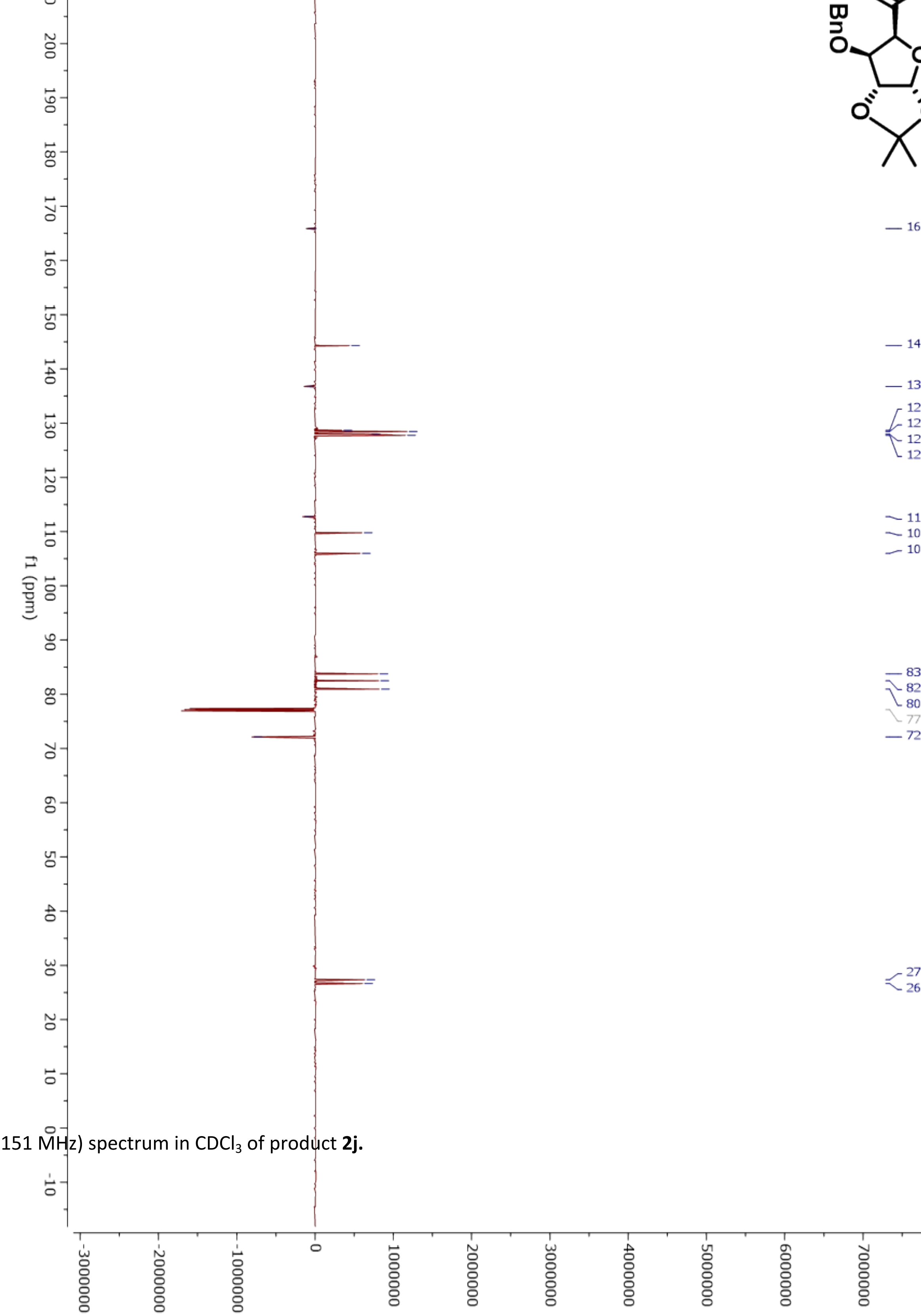

Figure S19: ^{13}C-NMR (151 MHz) spectrum in $CDCl_3$ of product **2j.**

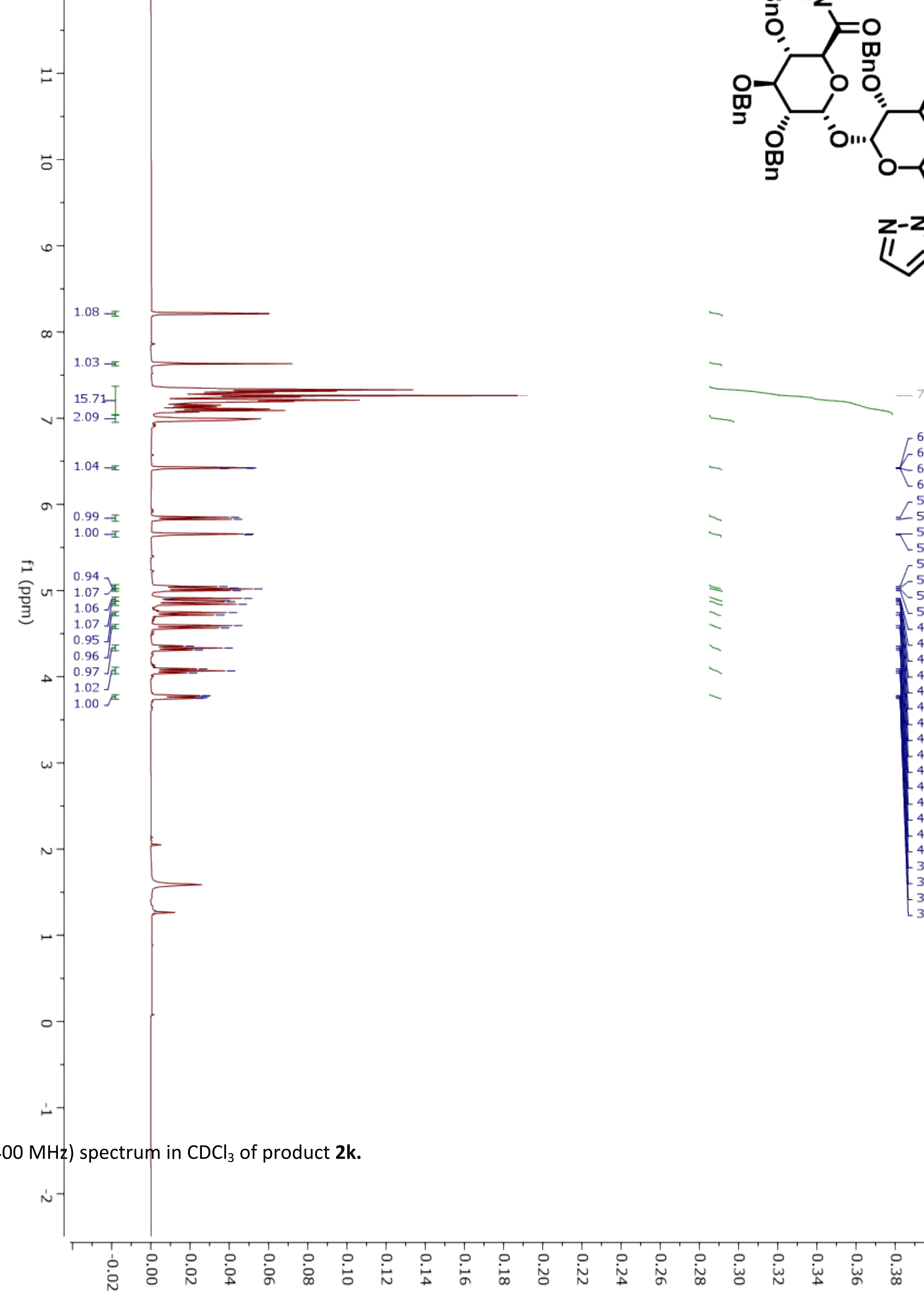

Figure S20: ^{1}H-NMR (400 MHz) spectrum in $CDCl_3$ of product **2k.**

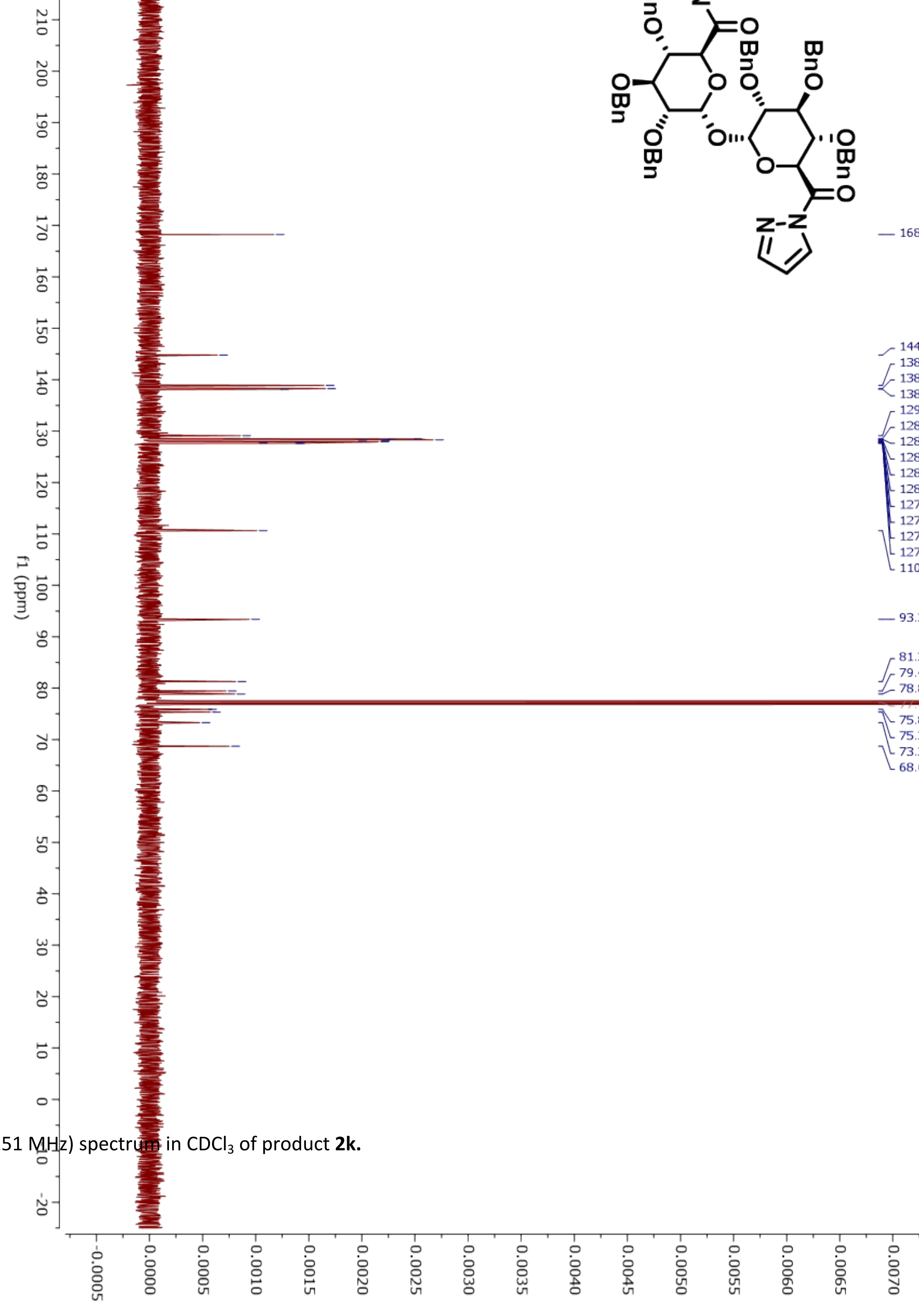

Figure S21: ^{13}C-NMR (151 MHz) spectrum in $CDCl_3$ of product **2k.**

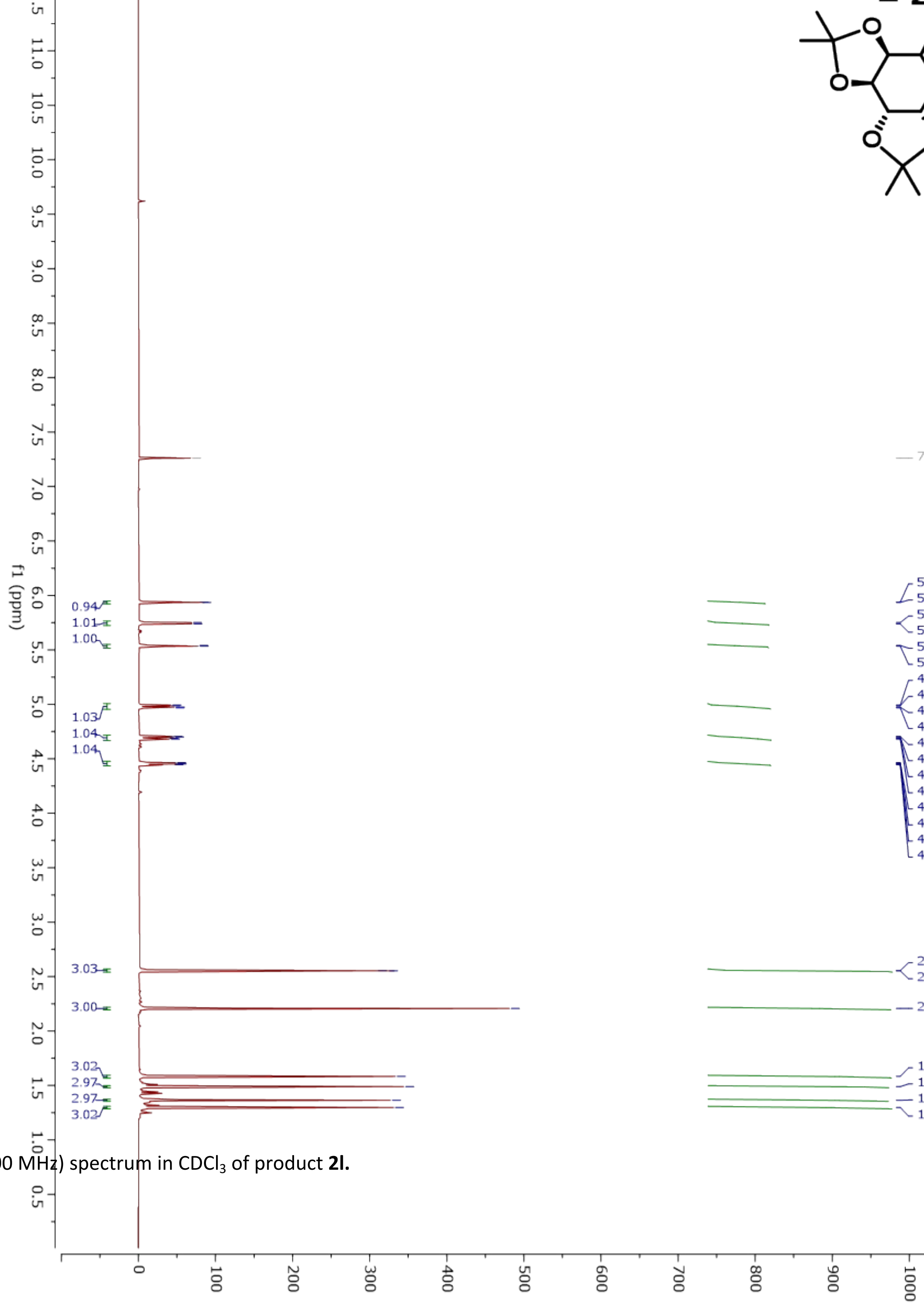

Figure S22: ^{1}H-NMR (400 MHz) spectrum in $CDCl_3$ of product **2l.**

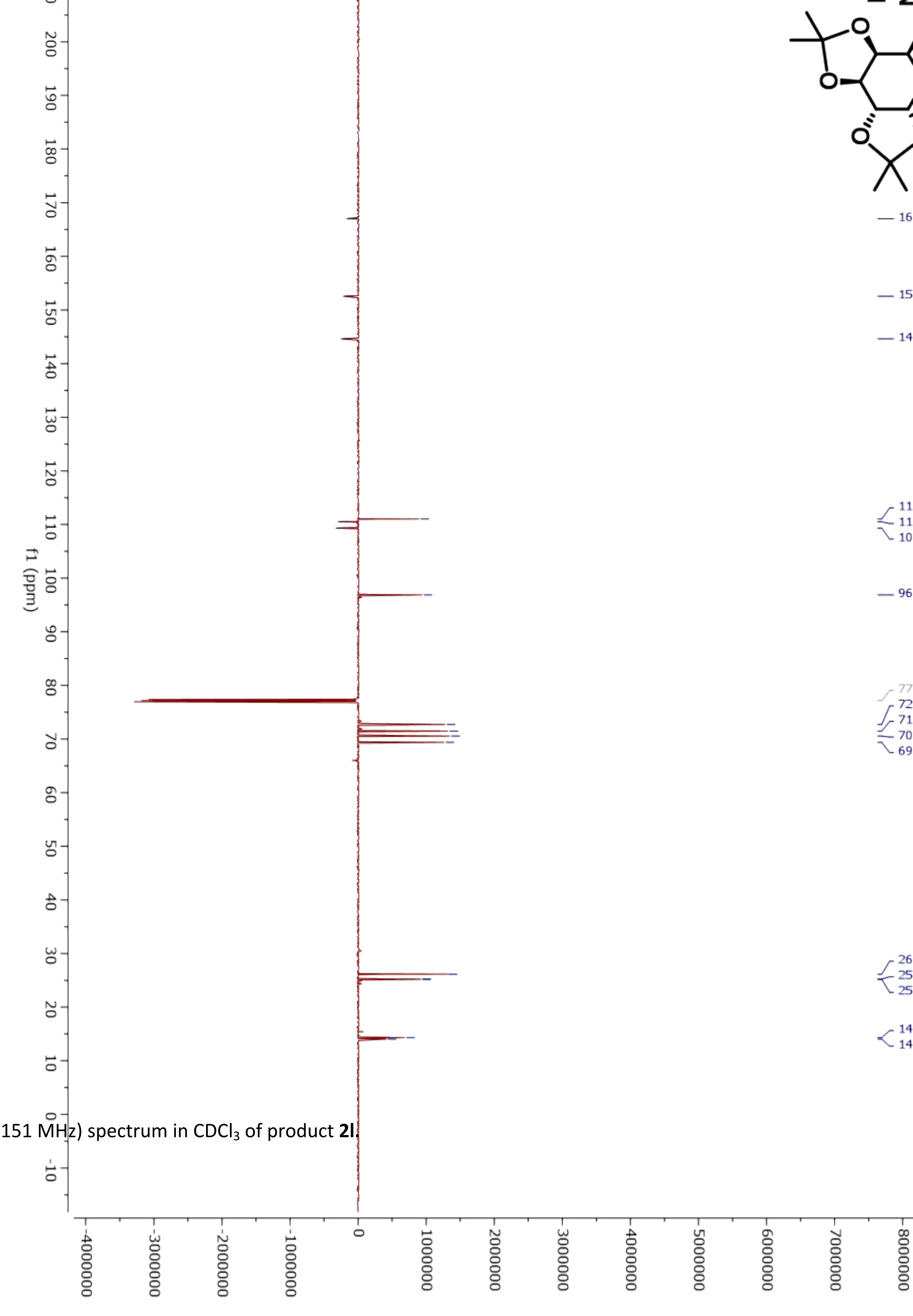

Figure S23: ^{13}C-NMR (151 MHz) spectrum in $CDCl_3$ of product **2l**.

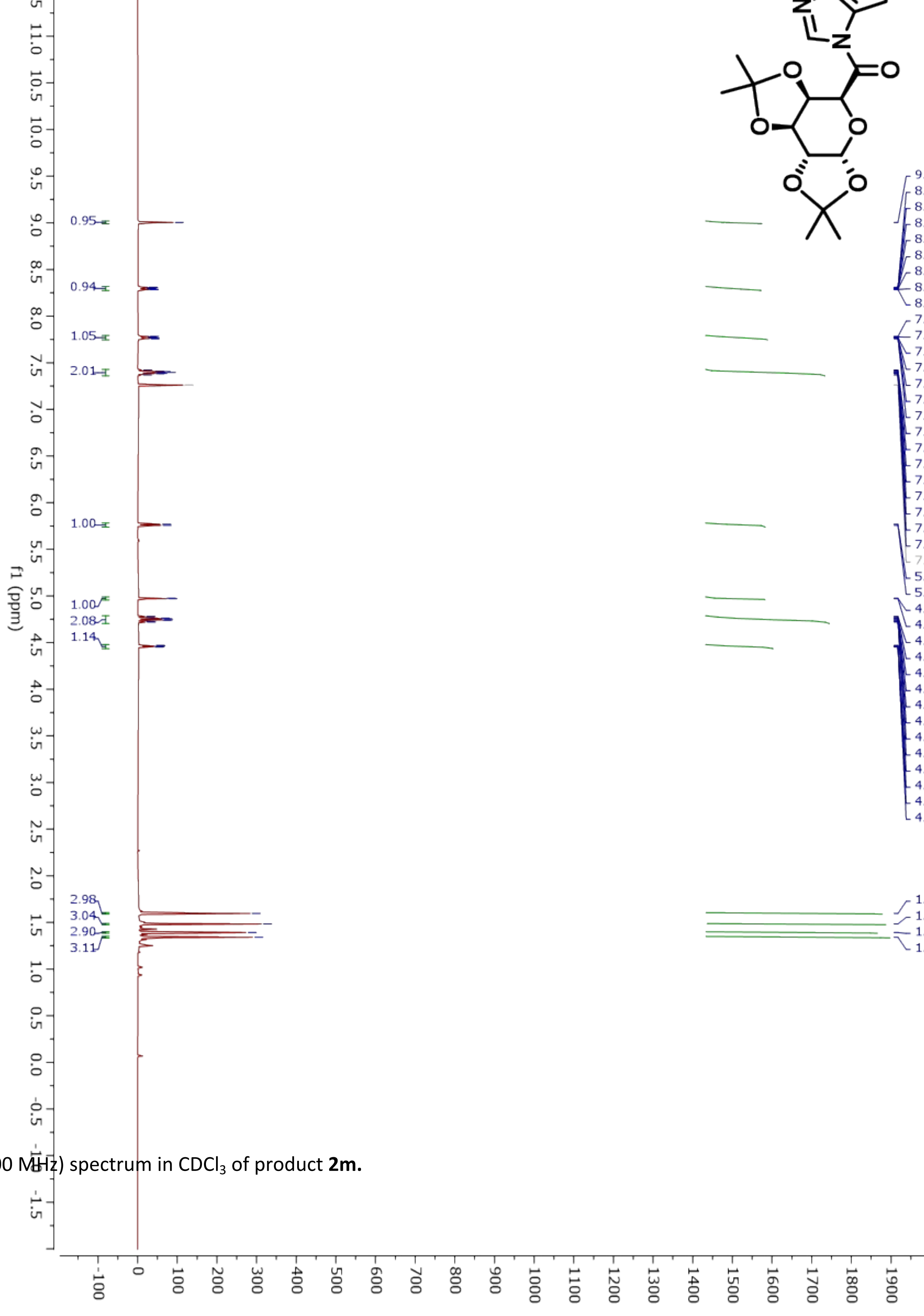

Figure S24: ^{1}H-NMR (400 MHz) spectrum in $CDCl_3$ of product **2m.**

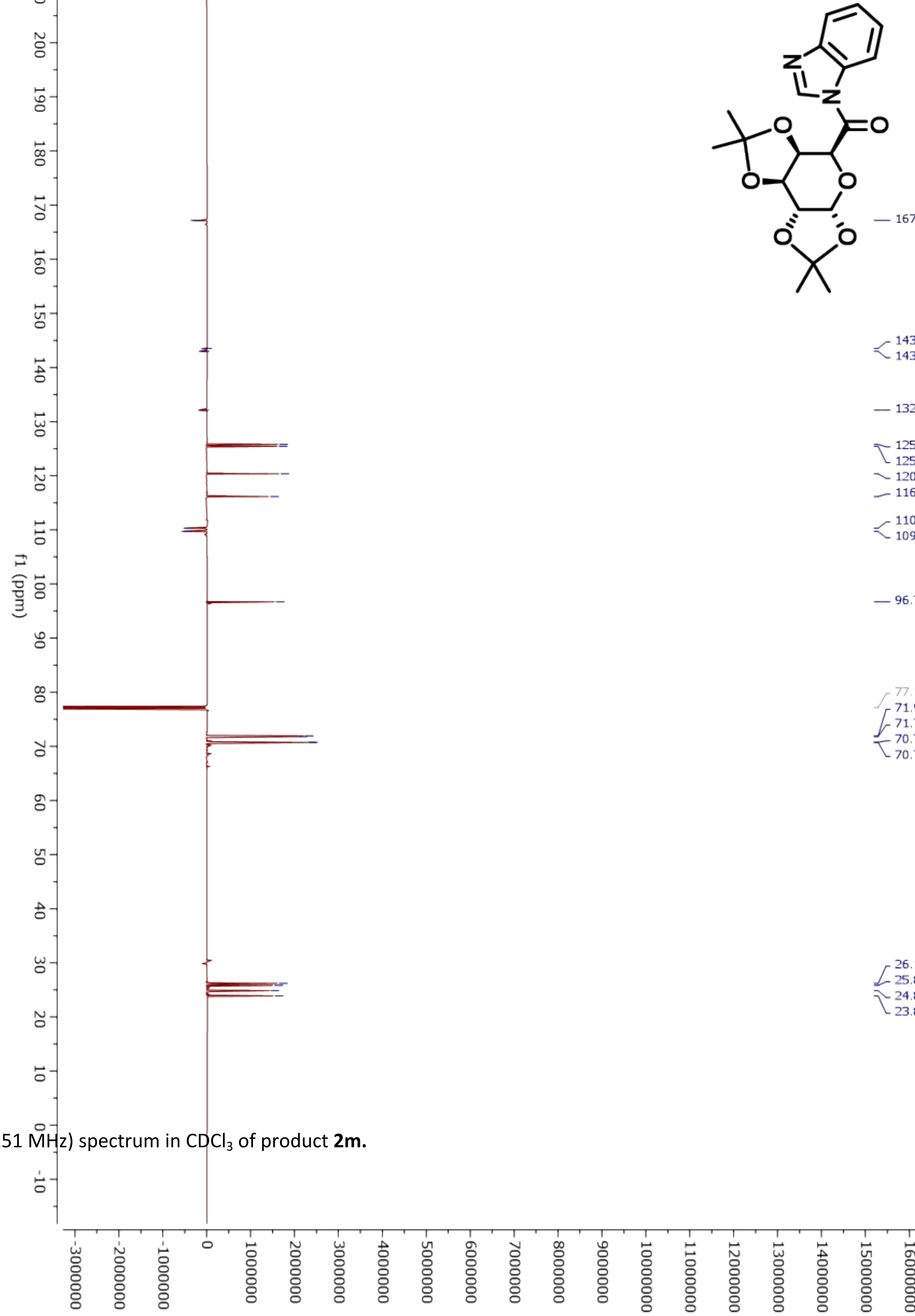

Figure S25: ^{13}C-NMR (151 MHz) spectrum in $CDCl_3$ of product **2m.**

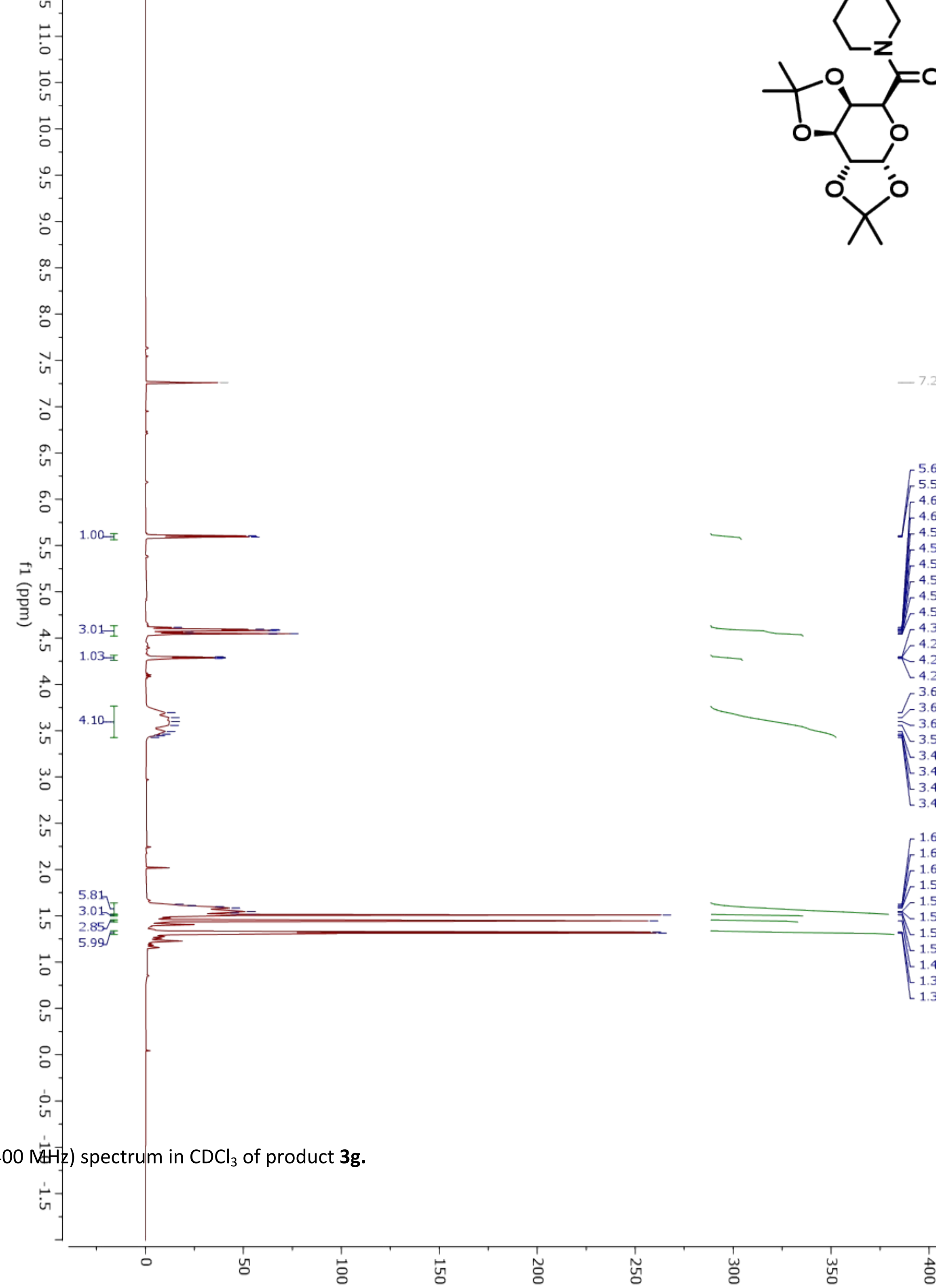

Figure S26: ^{1}H-NMR (400 MHz) spectrum in $CDCl_3$ of product **3g.**

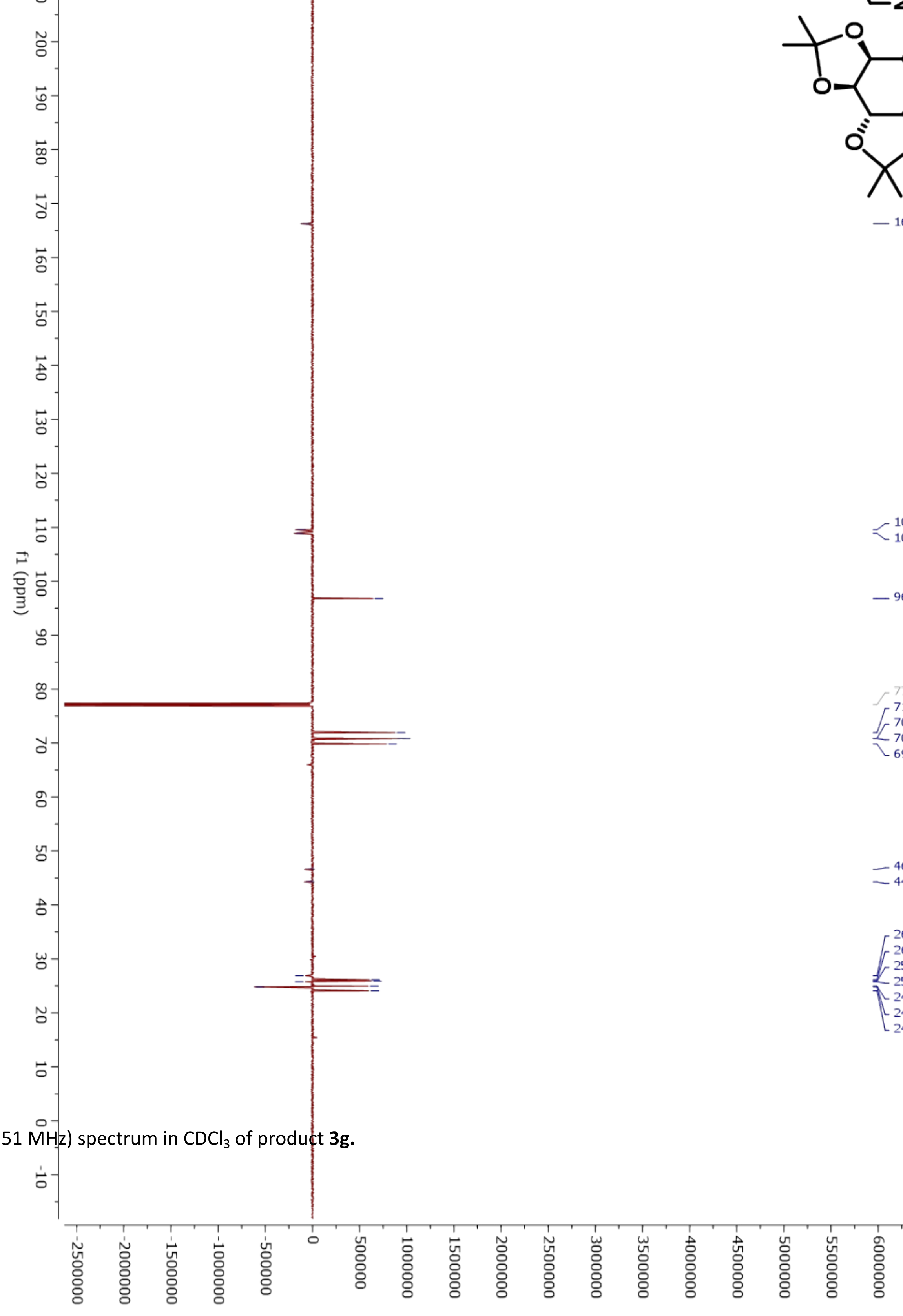

Figure S27: ^{13}C-NMR (151 MHz) spectrum in $CDCl_3$ of product **3g.**

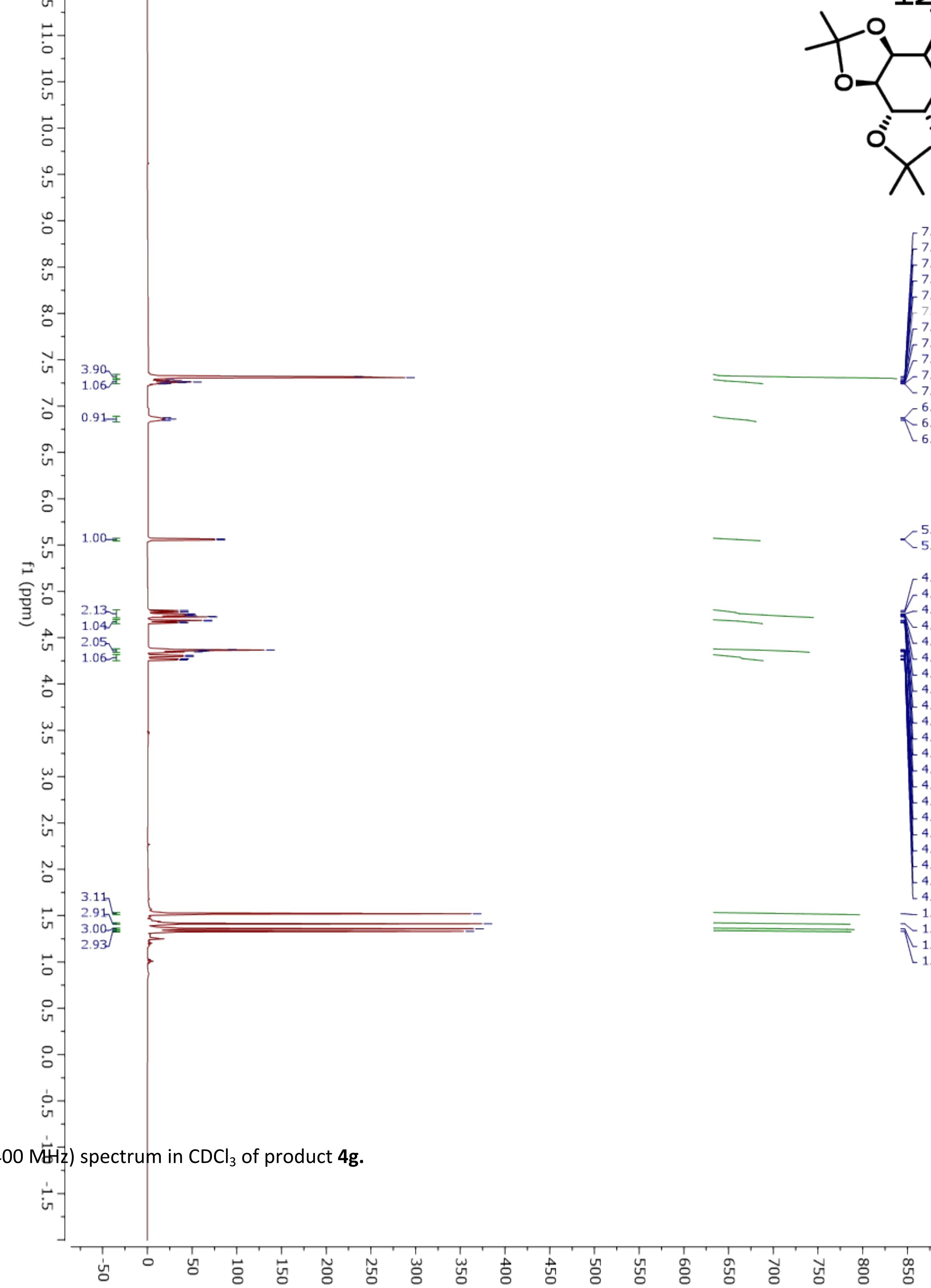

Figure S28: ^{1}H-NMR (400 MHz) spectrum in $CDCl_3$ of product **4g.**

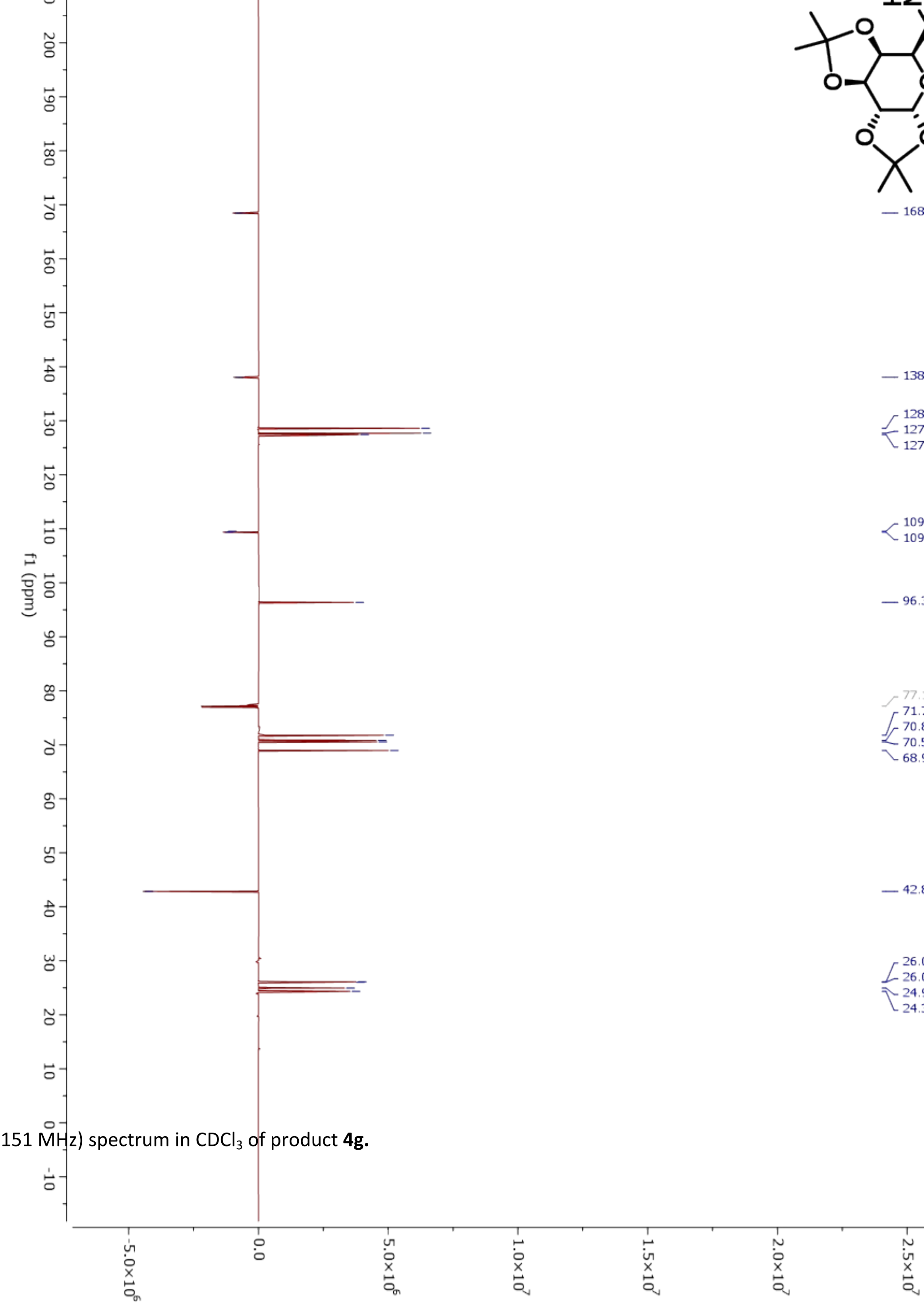

Figure S29: ^{13}C-NMR (151 MHz) spectrum in $CDCl_3$ of product **4g.**

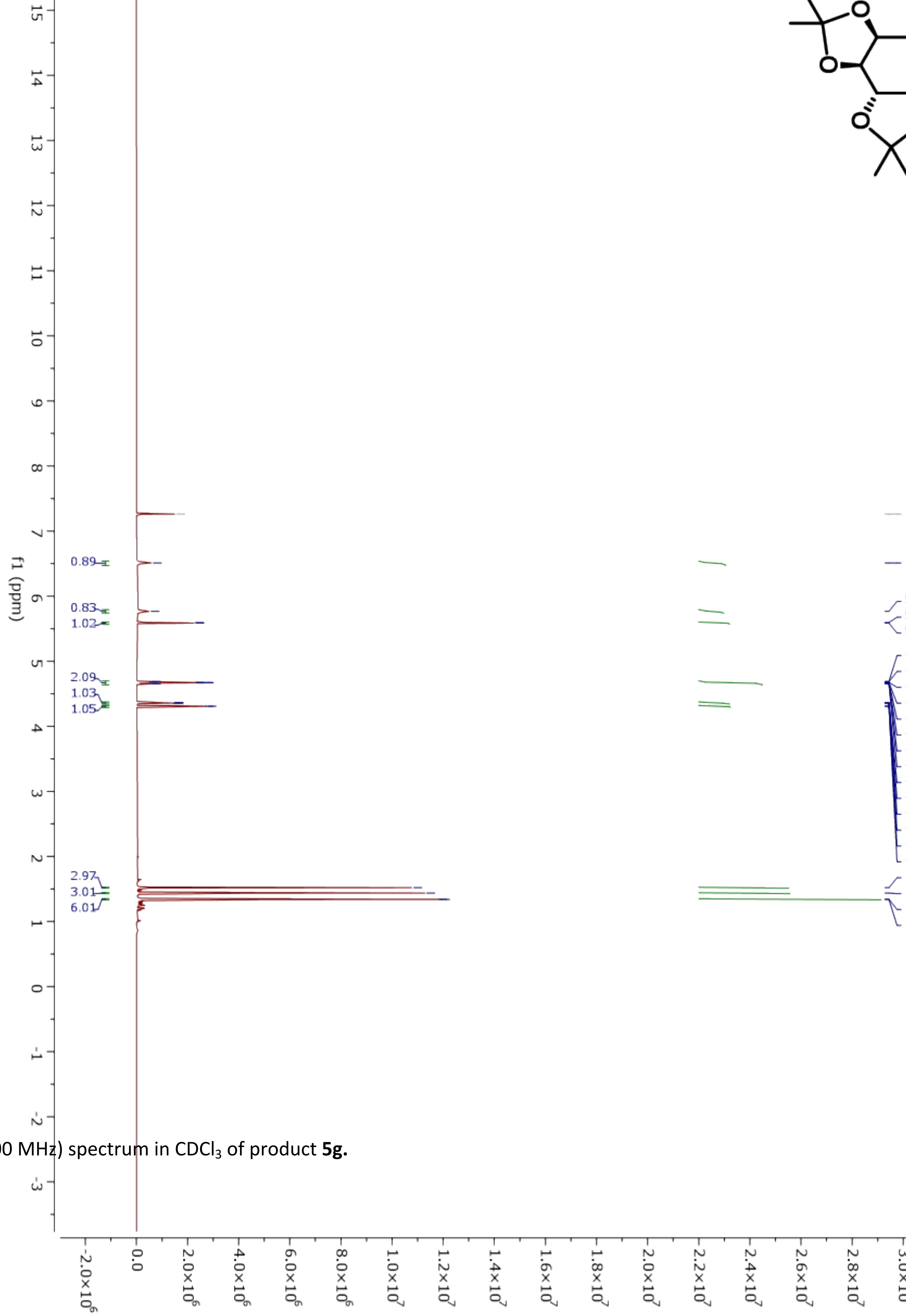

Figure S30: ^{1}H-NMR (600 MHz) spectrum in $CDCl_3$ of product **5g.**

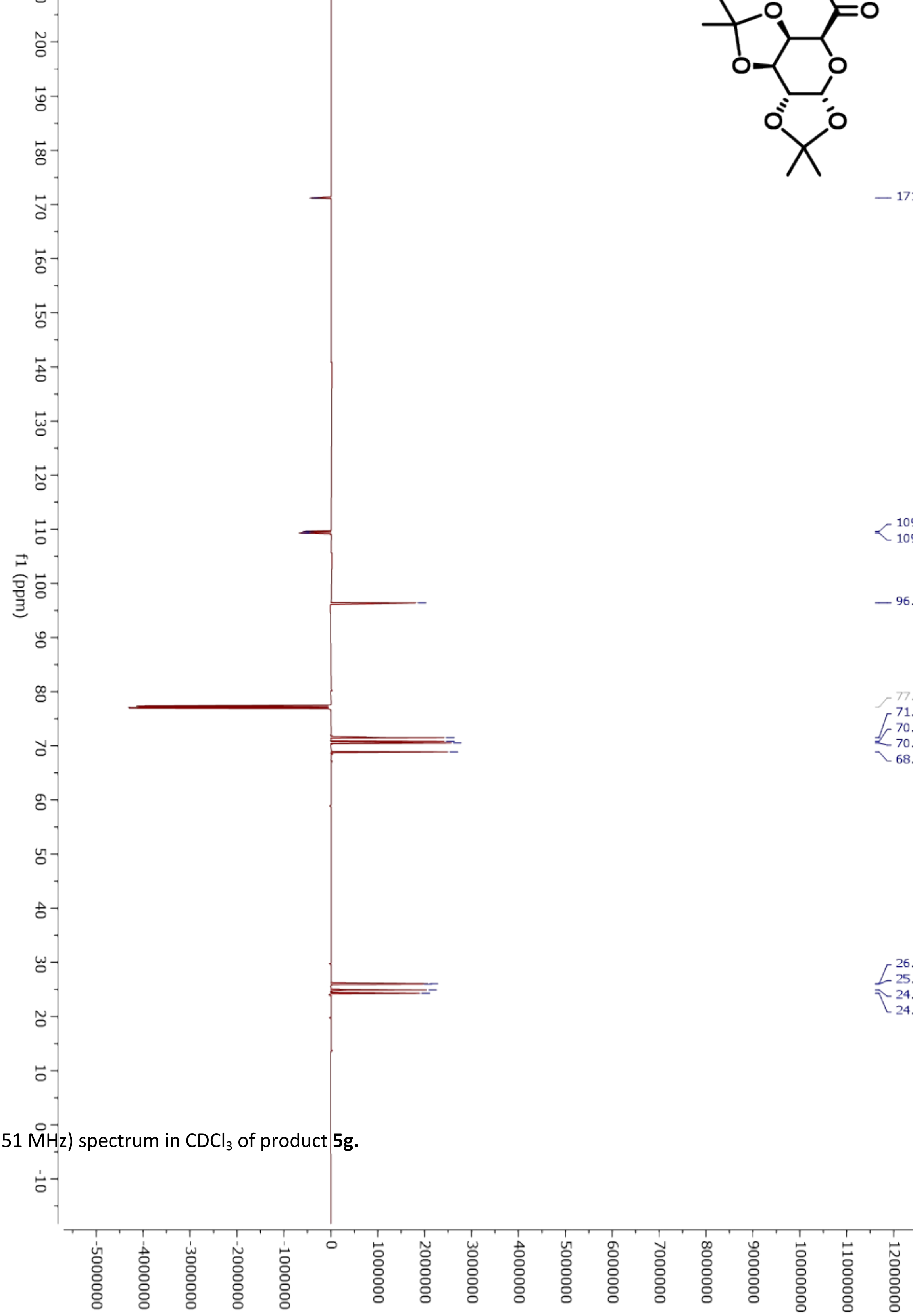

Figure S31: ^{13}C-NMR (151 MHz) spectrum in $CDCl_3$ of product **5g.**

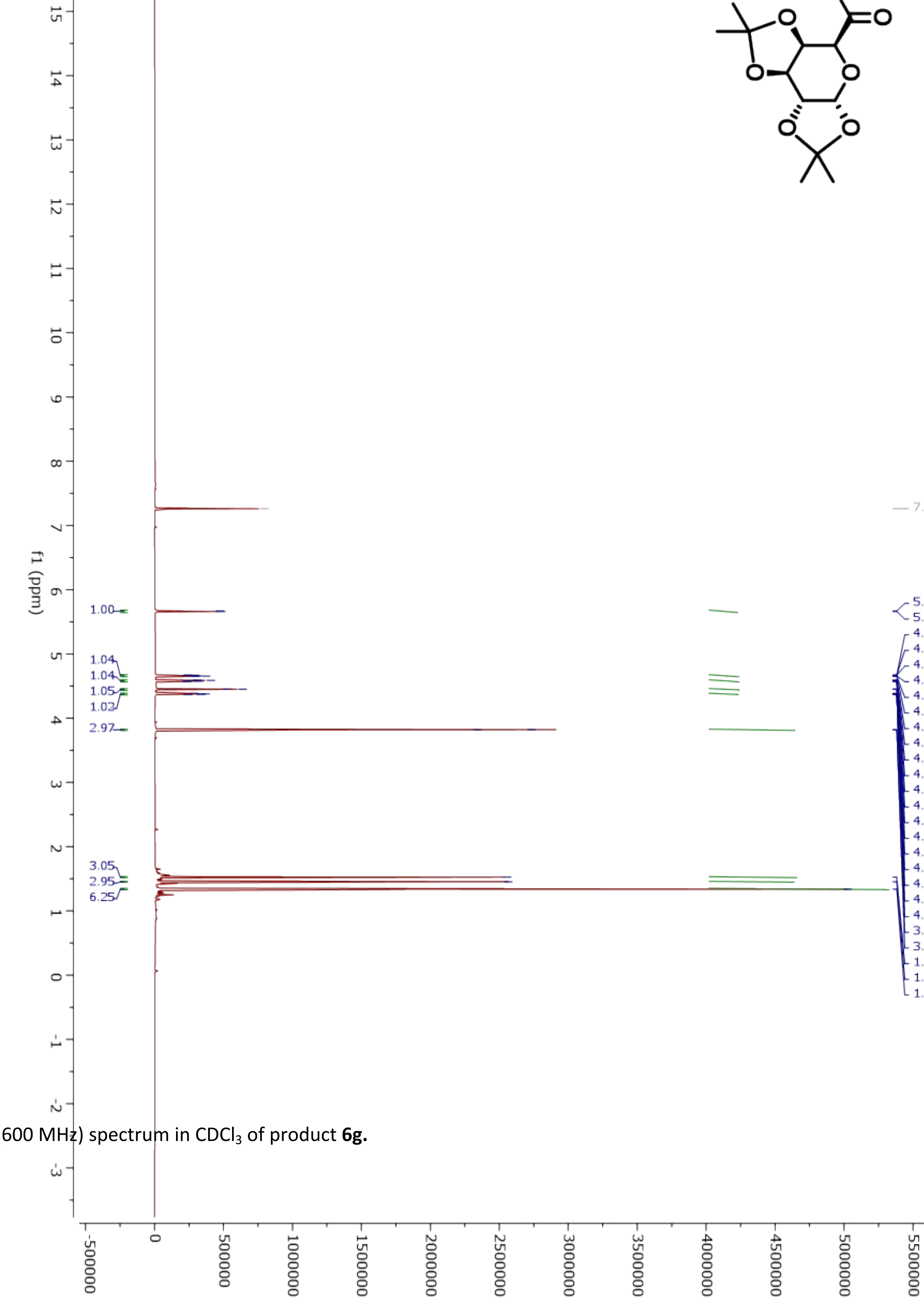

Figure S32: ^{1}H-NMR (600 MHz) spectrum in $CDCl_3$ of product **6g.**

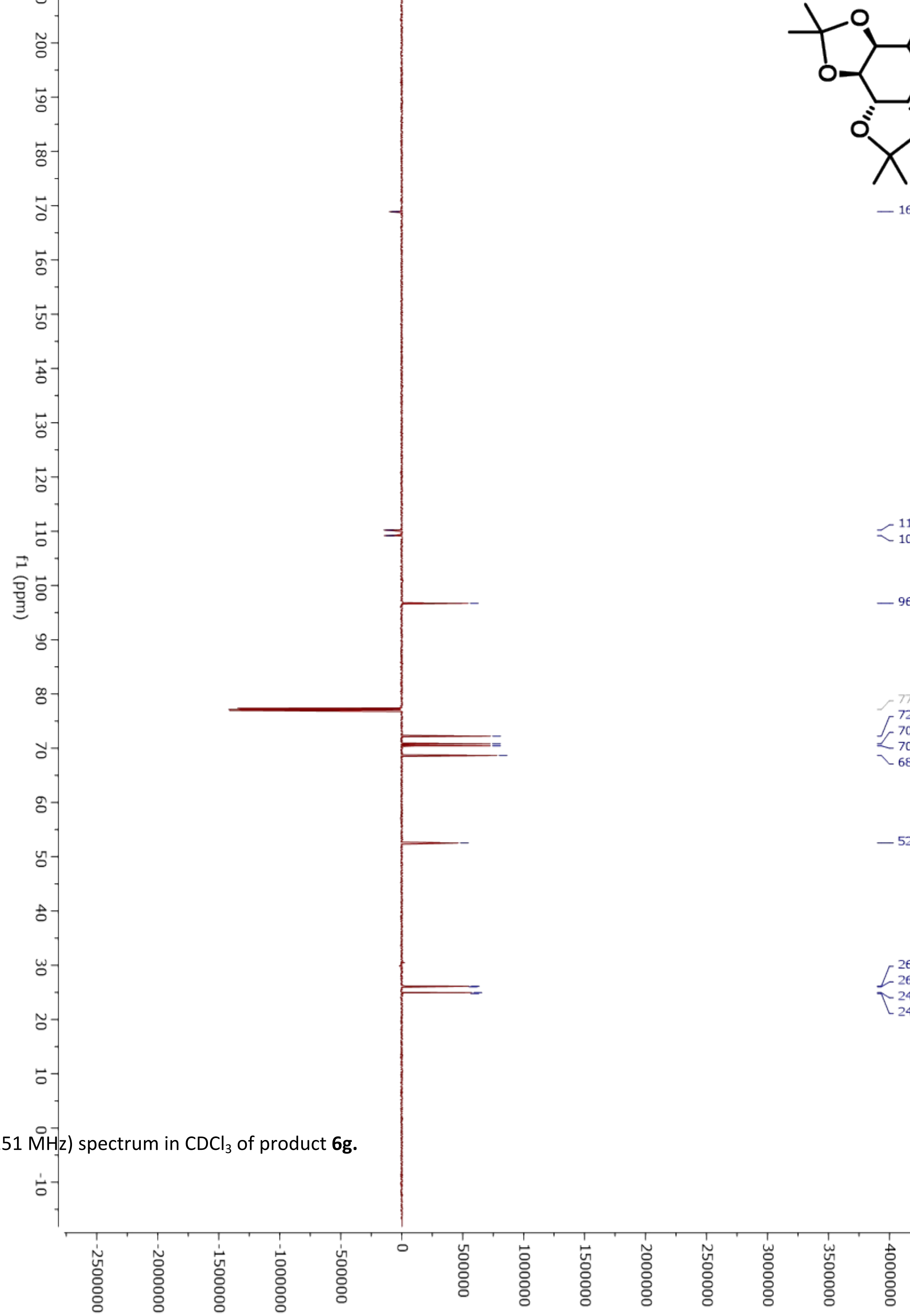

Figure S33: ^{13}C-NMR (151 MHz) spectrum in $CDCl_3$ of product **6g.**

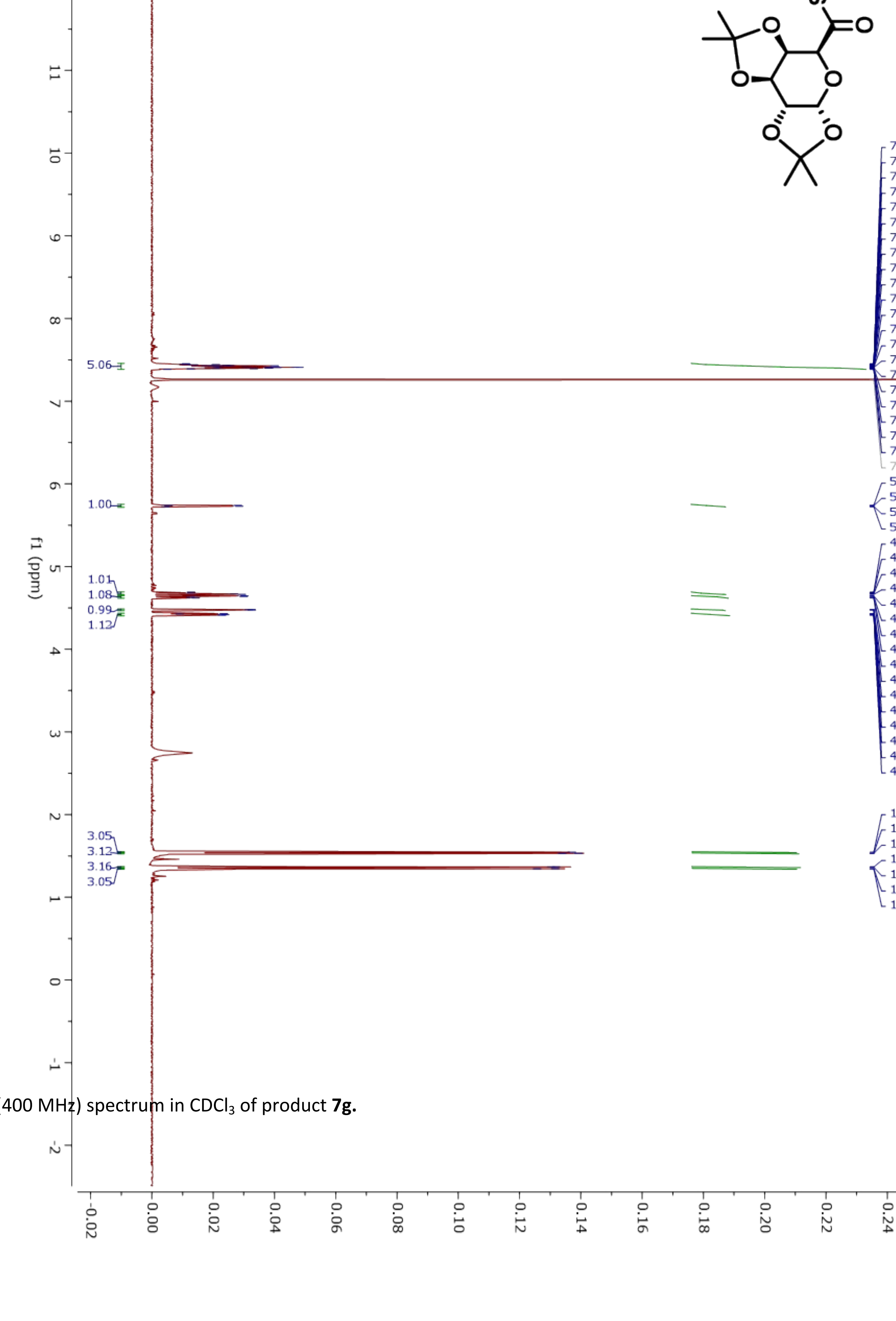

Figure S34: ^{1}H-NMR (400 MHz) spectrum in $CDCl_3$ of product **7g.**

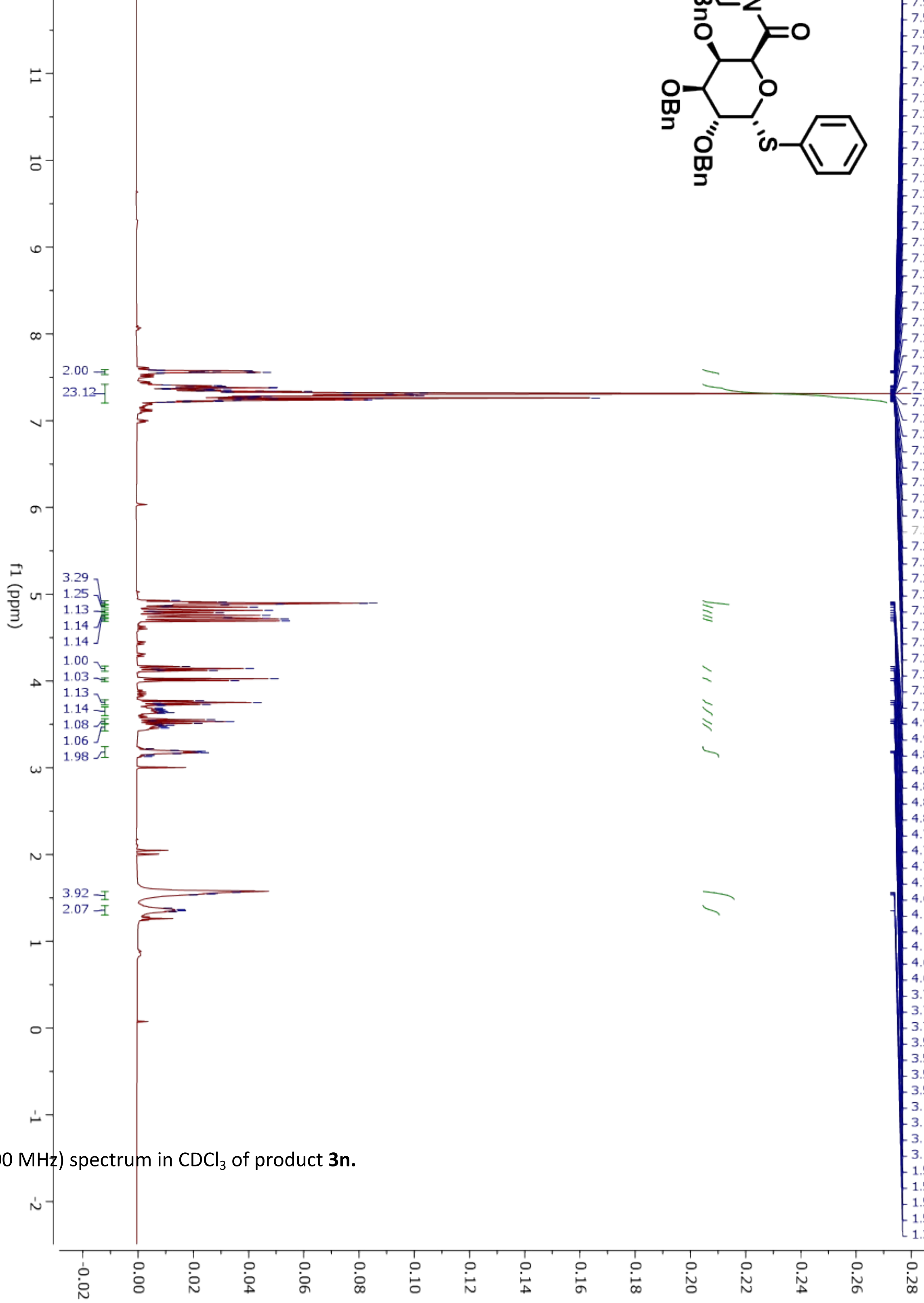

Figure S35: ^{1}H-NMR (400 MHz) spectrum in $CDCl_3$ of product **3n.**

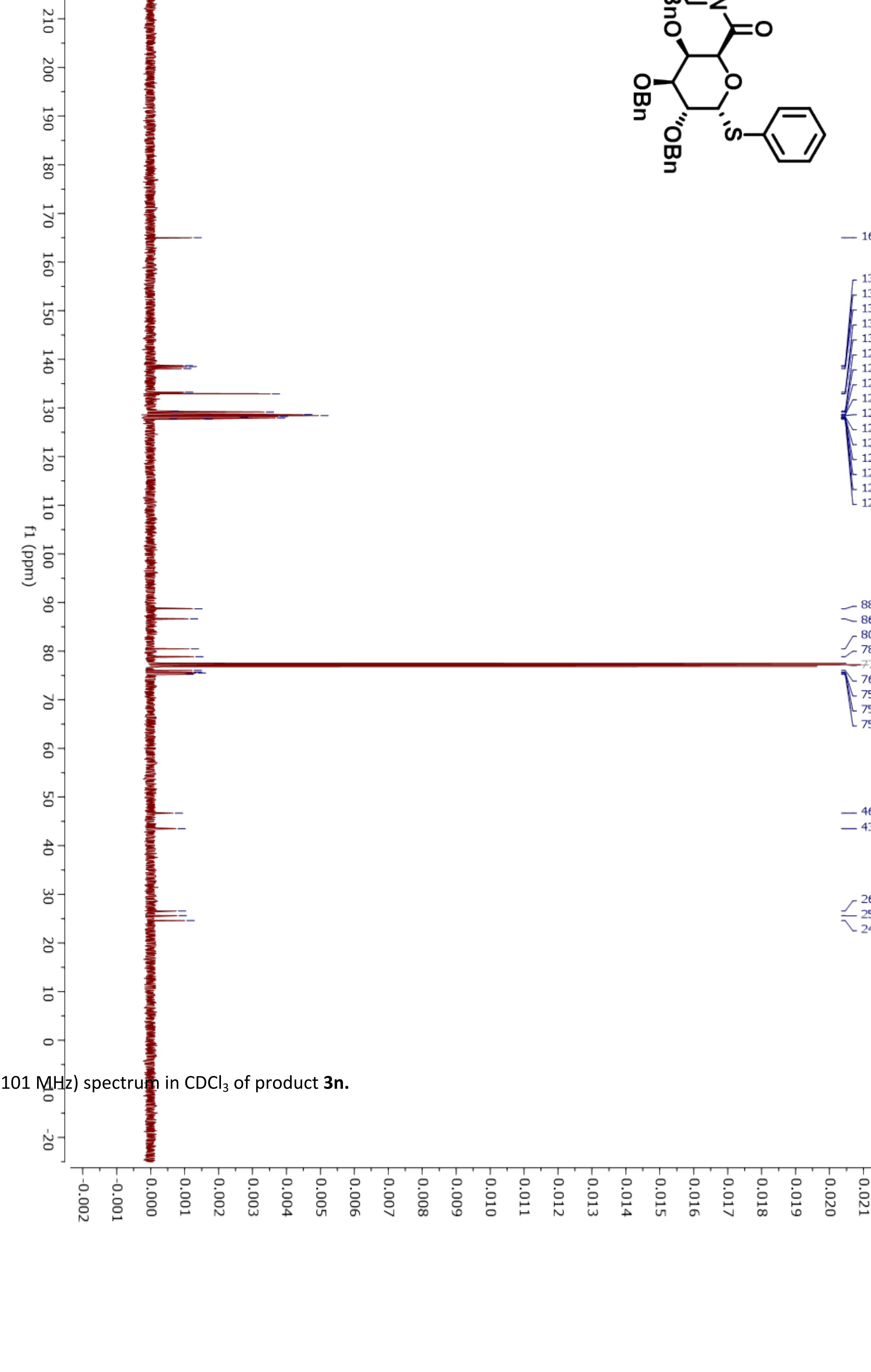

Figure S36: ^{13}C-NMR (101 MHz) spectrum in $CDCl_3$ of product **3n.**

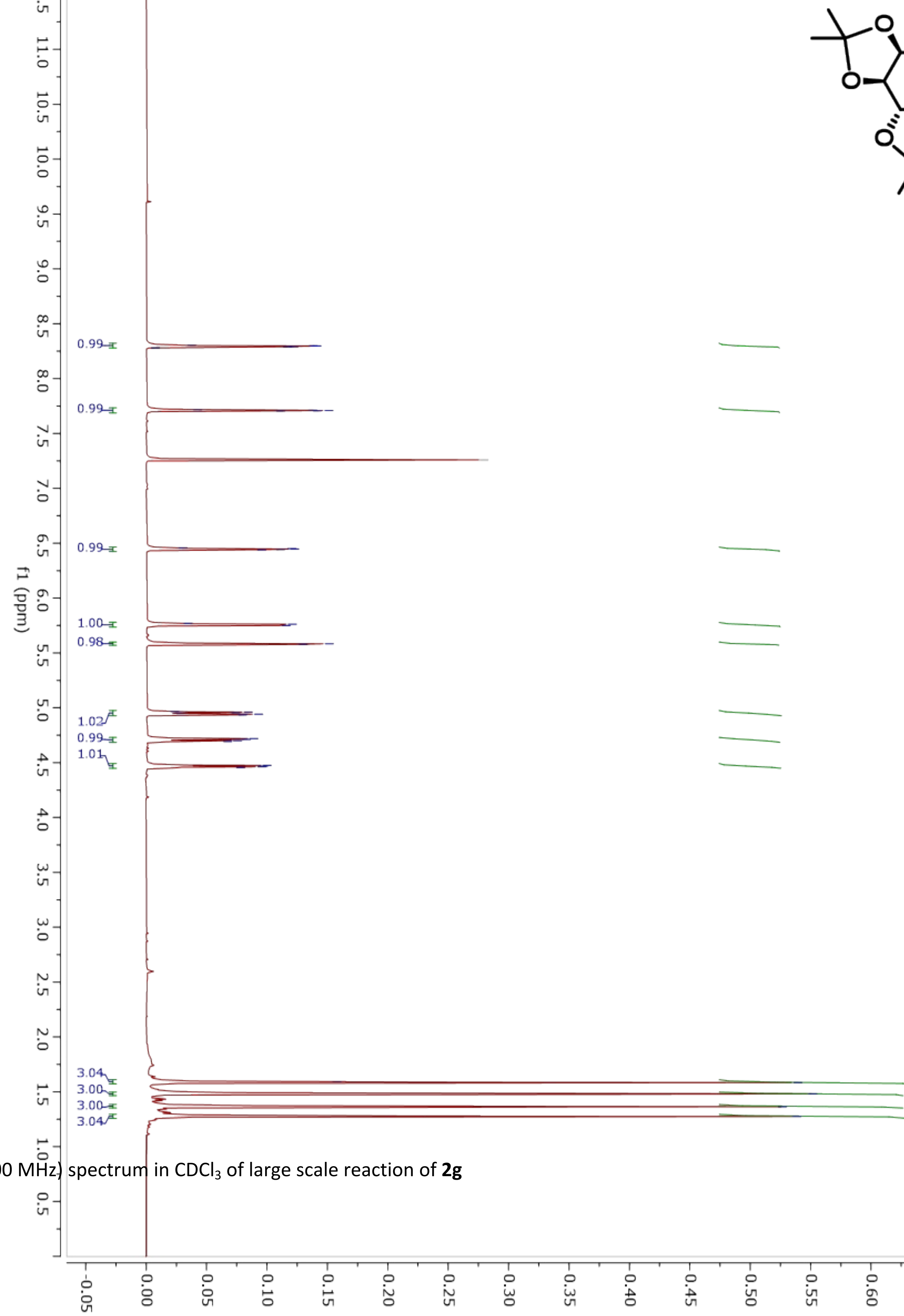

Figure S37: ^{1}H-NMR (400 MHz) spectrum in $CDCl_3$ of large scale reaction of **2g**

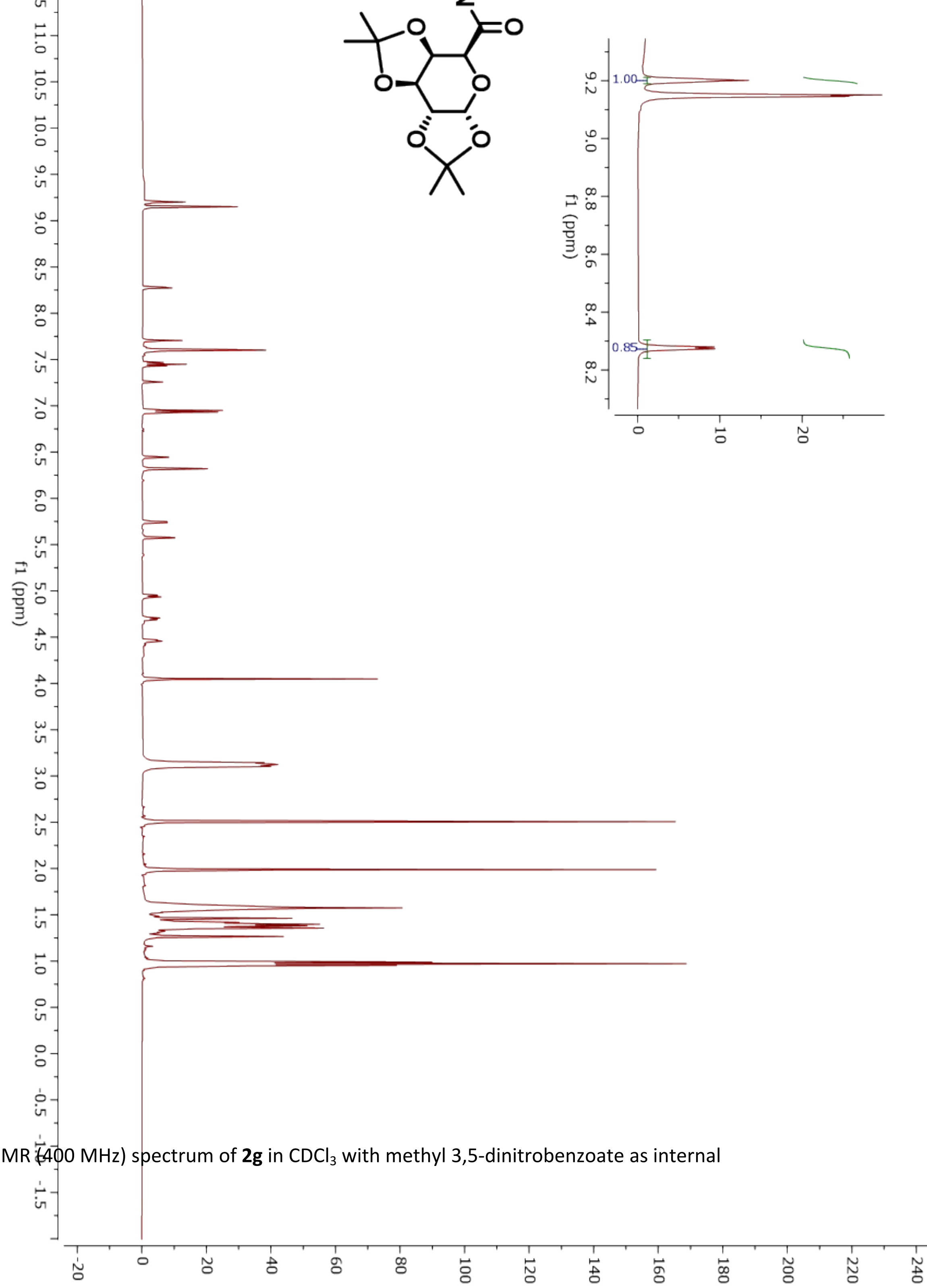

Figure S38: Crude ^{1}H-NMR (400 MHz) spectrum of **2g** in $CDCl_3$ with methyl 3,5-dinitrobenzoate as internal standard

11.2 NMR spectra recycled electrolyte

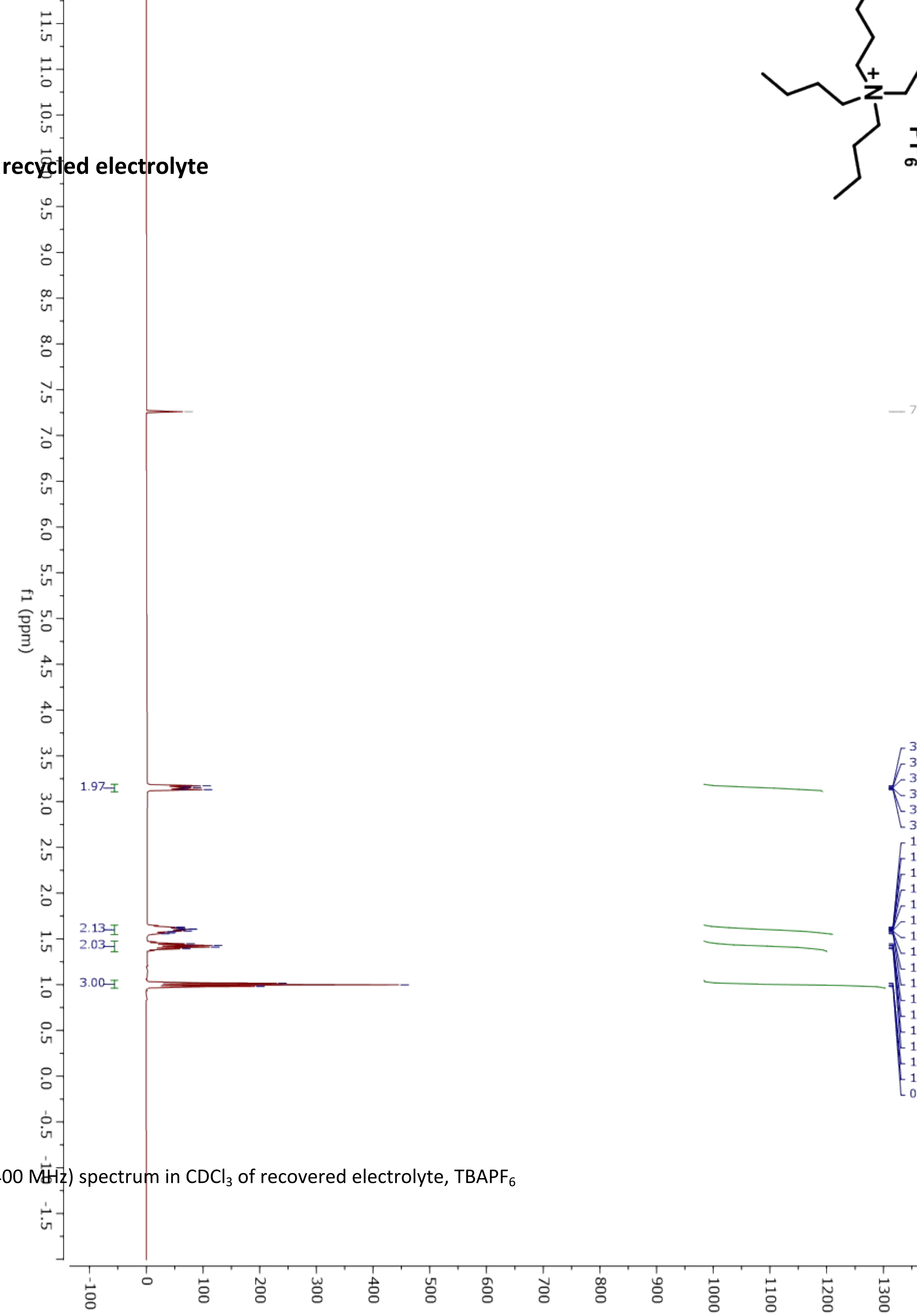

Figure S39: ^{1}H-NMR (400 MHz) spectrum in $CDCl_3$ of recovered electrolyte, $TBAPF_6$

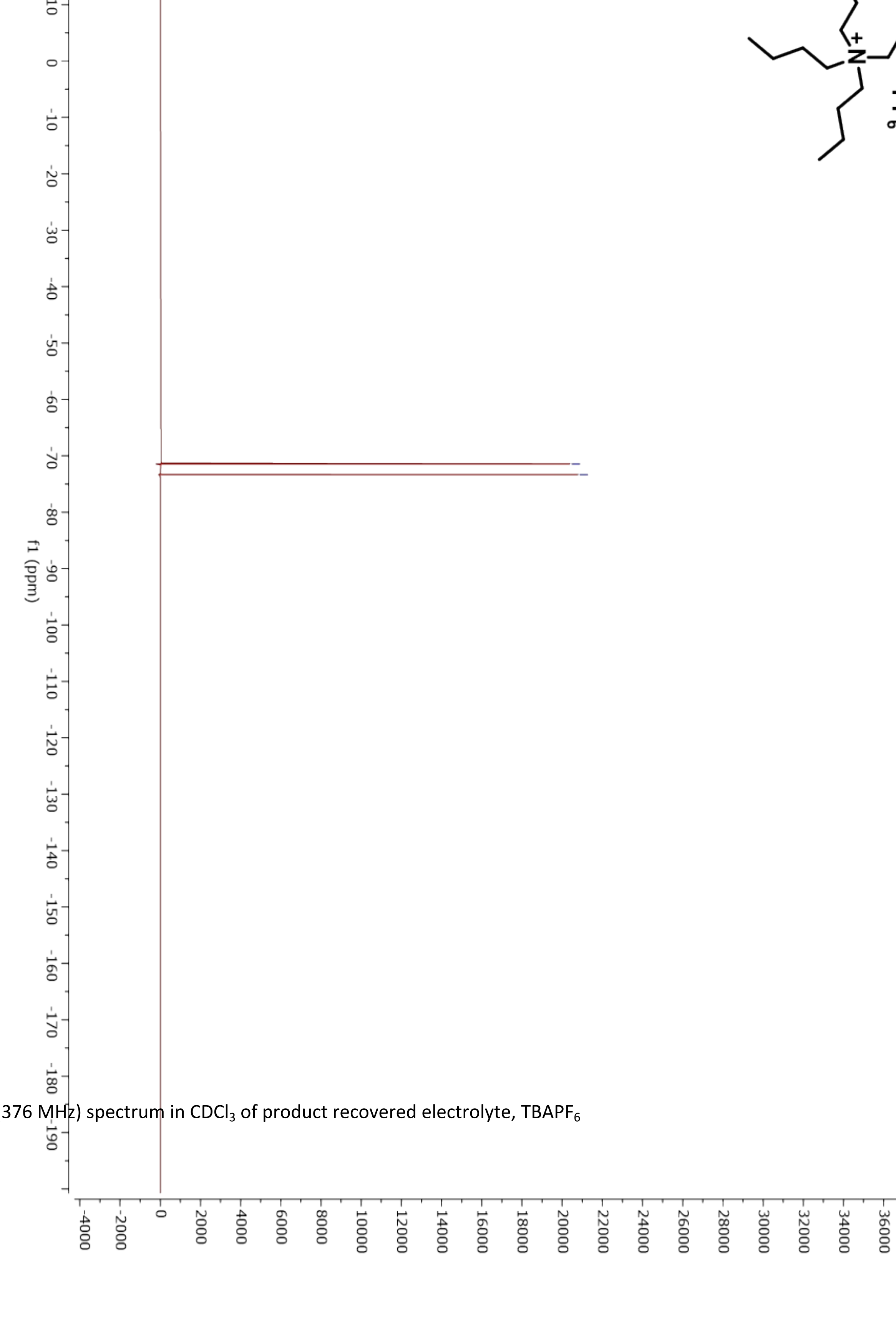

Figure S40: ^{19}F-NMR (376 MHz) spectrum in $CDCl_3$ of product recovered electrolyte, $TBAPF_6$

11.3 HRMS and TLC-MS spectra

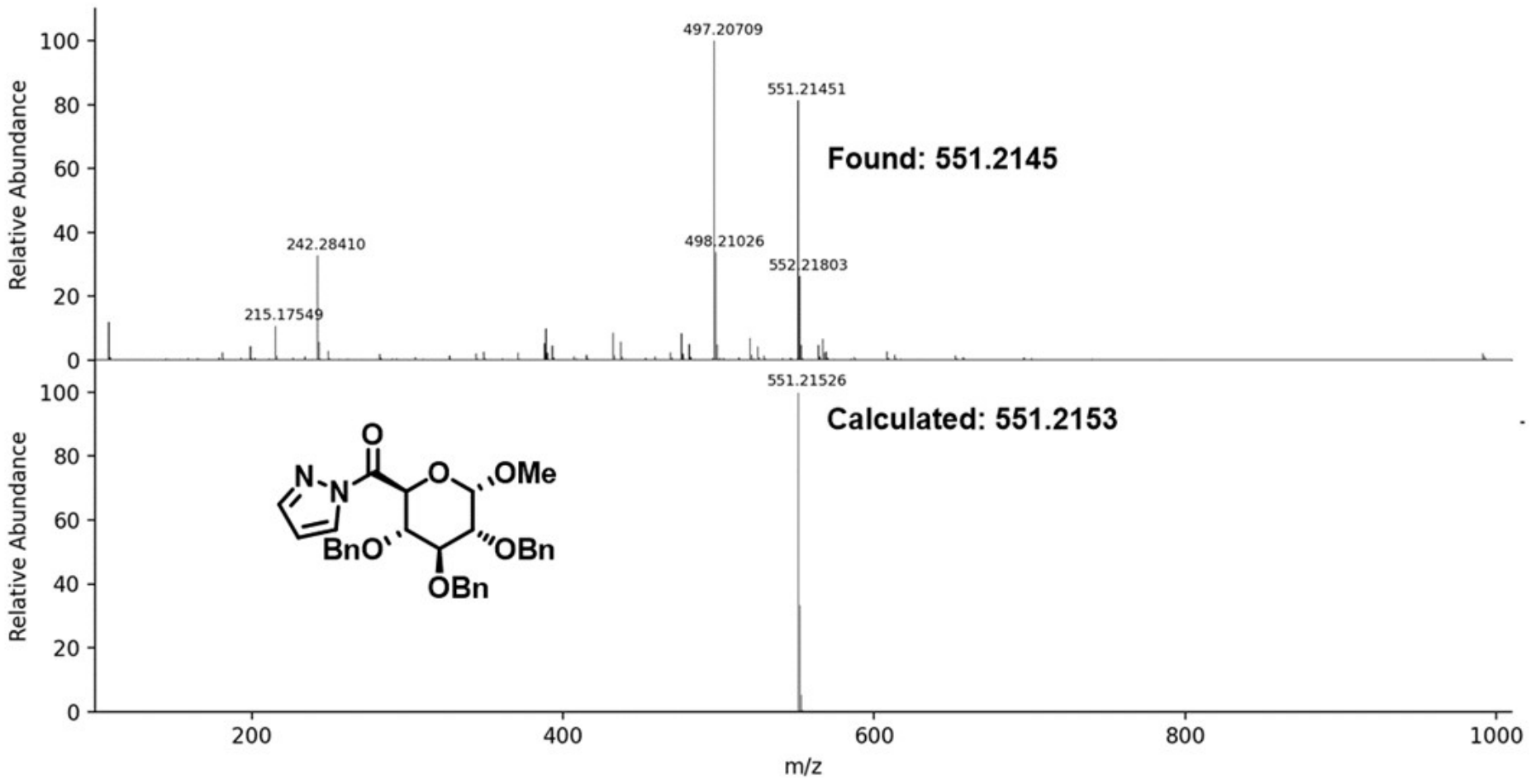

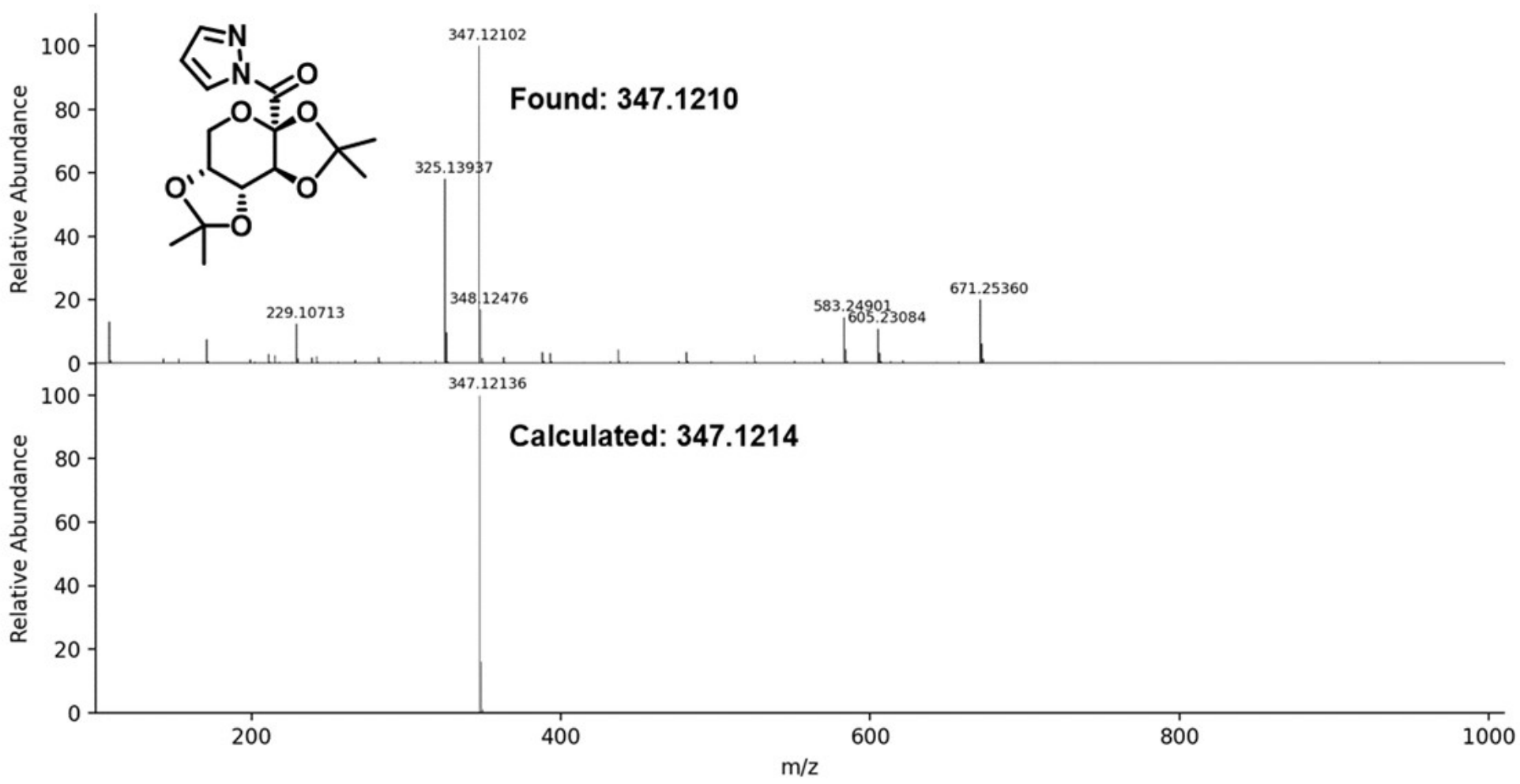

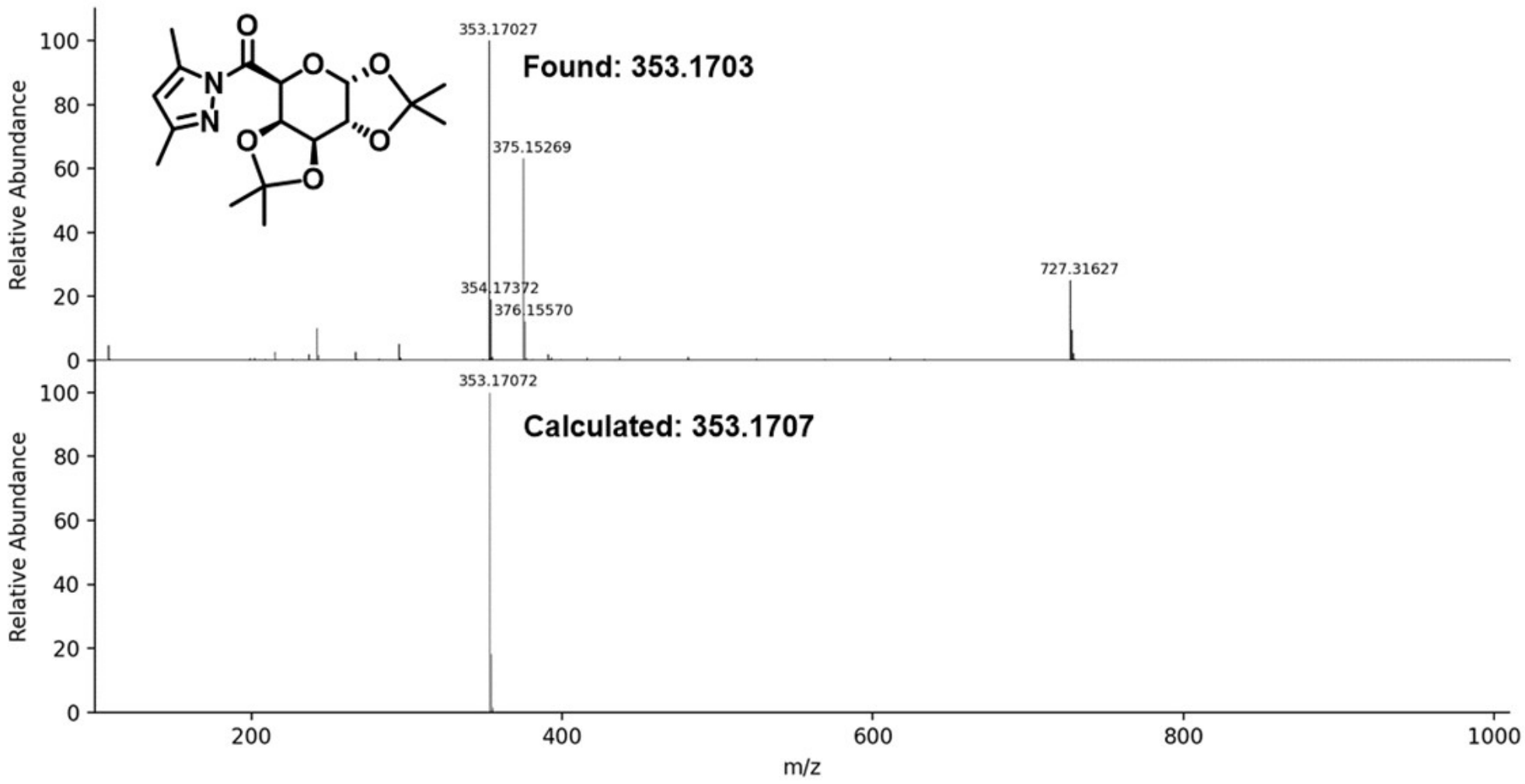

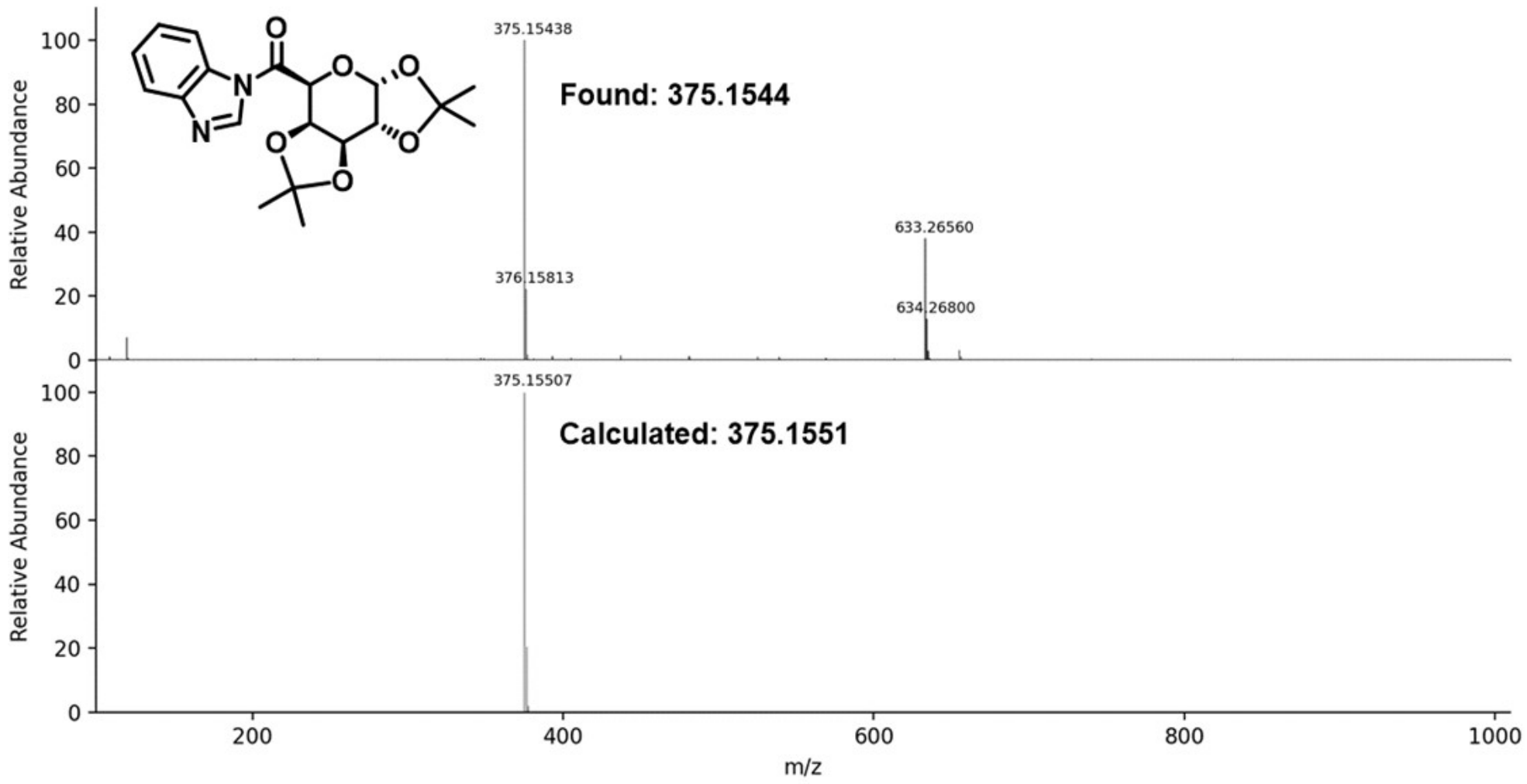

Found: 375.1544
Calculated: 375.1551
375.15438
376.15813
633.26560
634.26800
375.15507
Relative Abundance
m/z

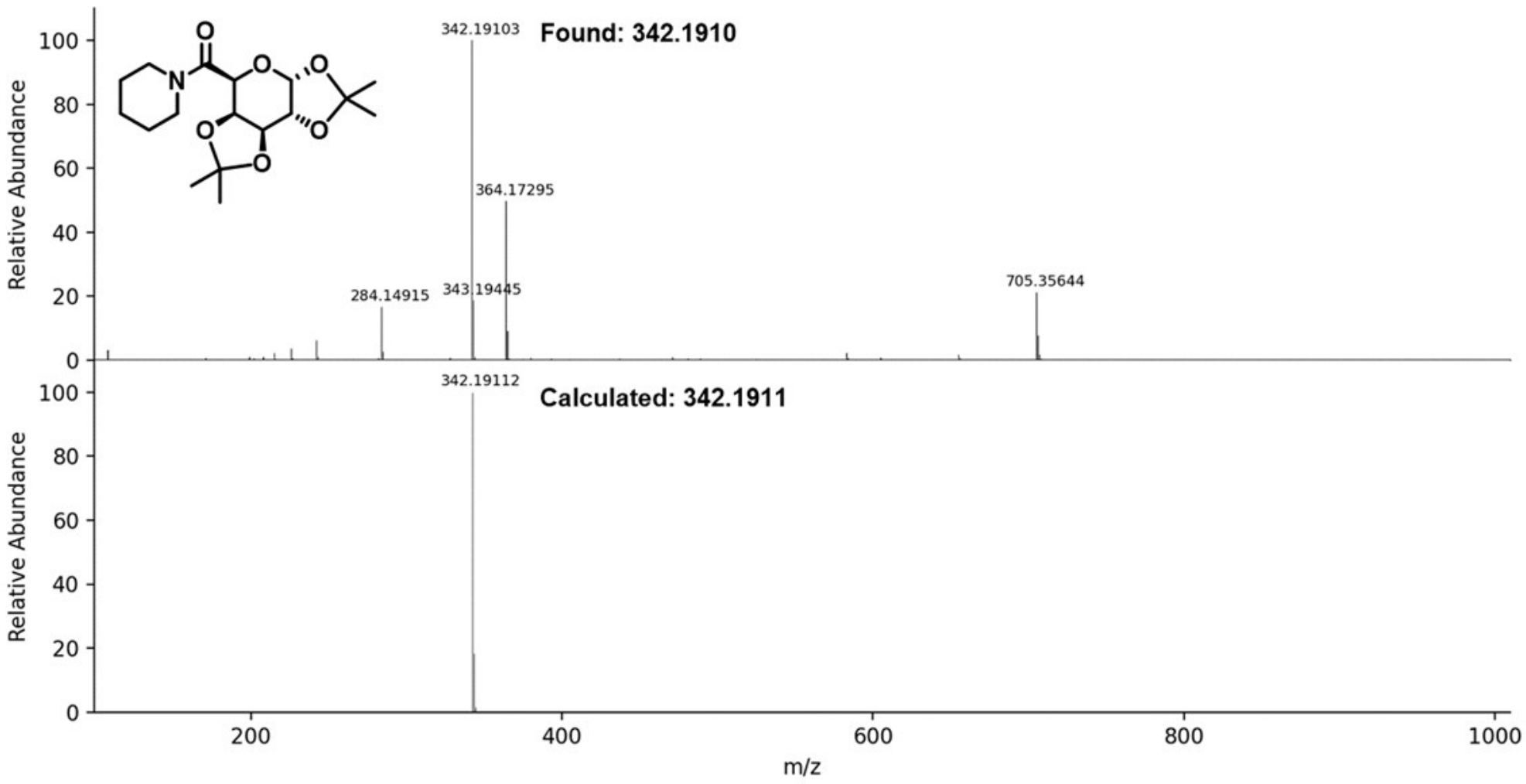

Found: 342.1910
Calculated: 342.1911
342.19103
364.17295
284.14915
343.19445
705.35644
342.19112
Relative Abundance
m/z

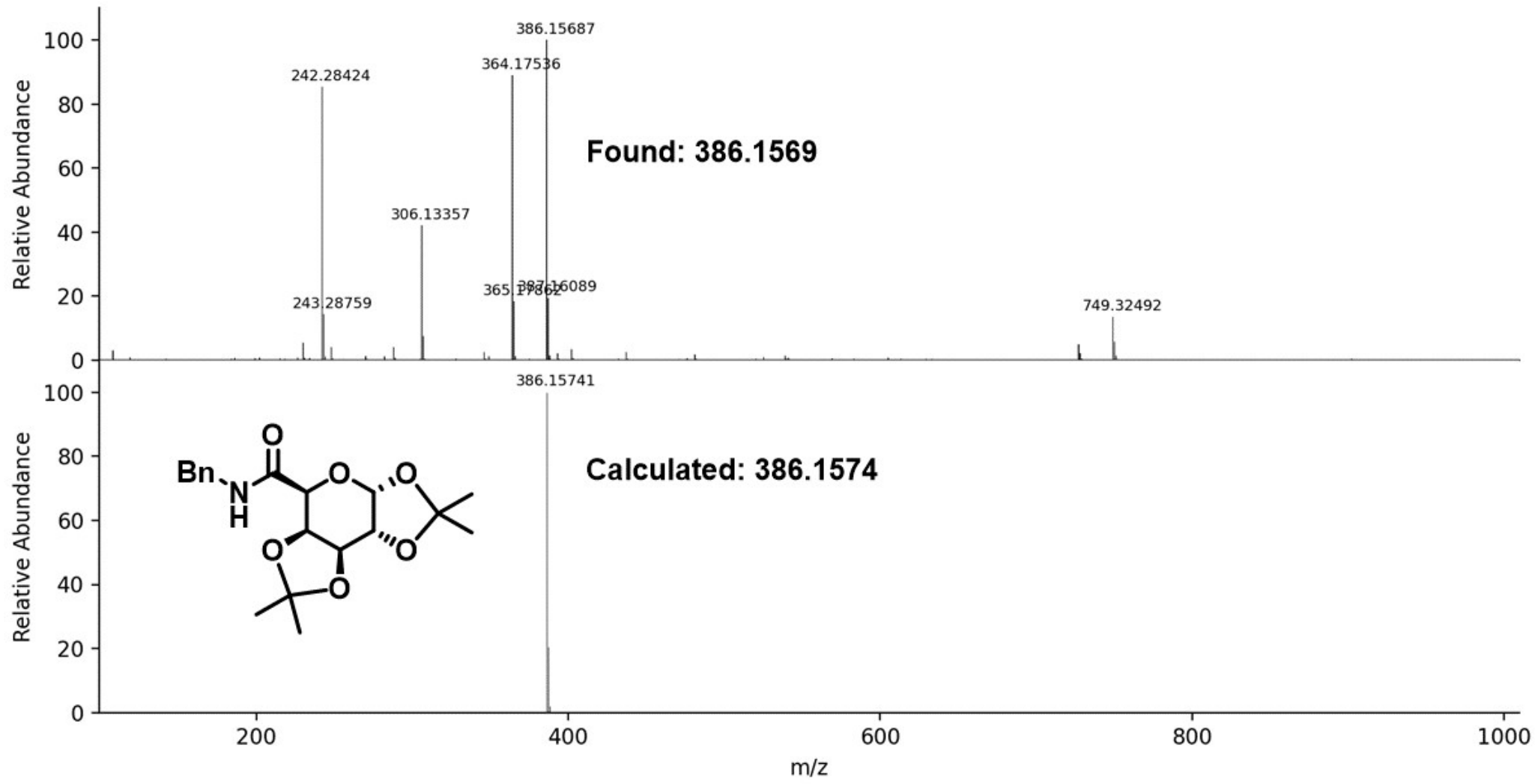

Found: 386.1569
Calculated: 386.1574
386.15687
364.17536
242.28424
306.13357
243.28759
365.17862
387.16089
749.32492
386.15741
Bn
Relative Abundance
m/z

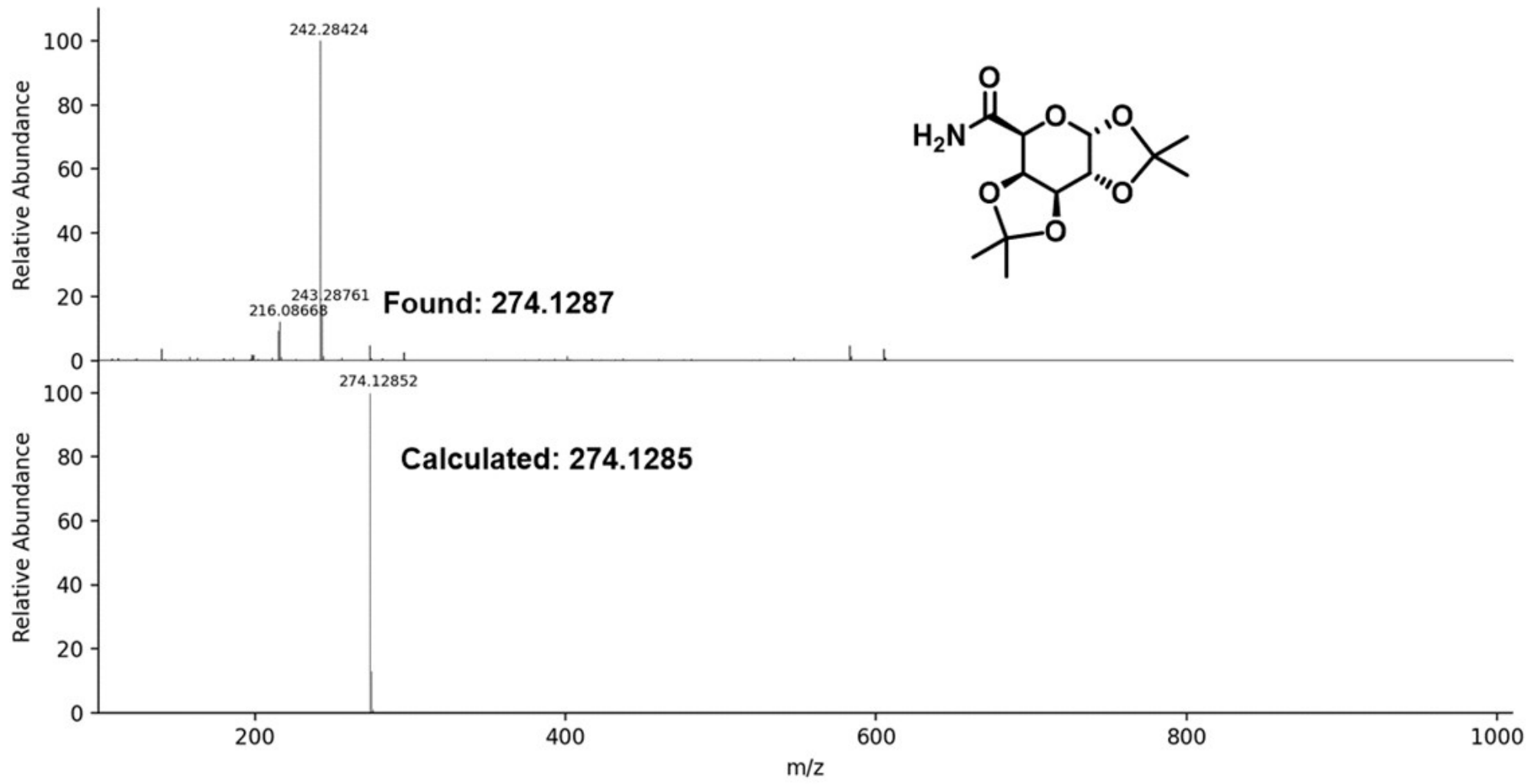
242.28424
243.28761
216.08668
Found: 274.1287
274.12852
Calculated: 274.1285
Relative Abundance
m/z
H_2N

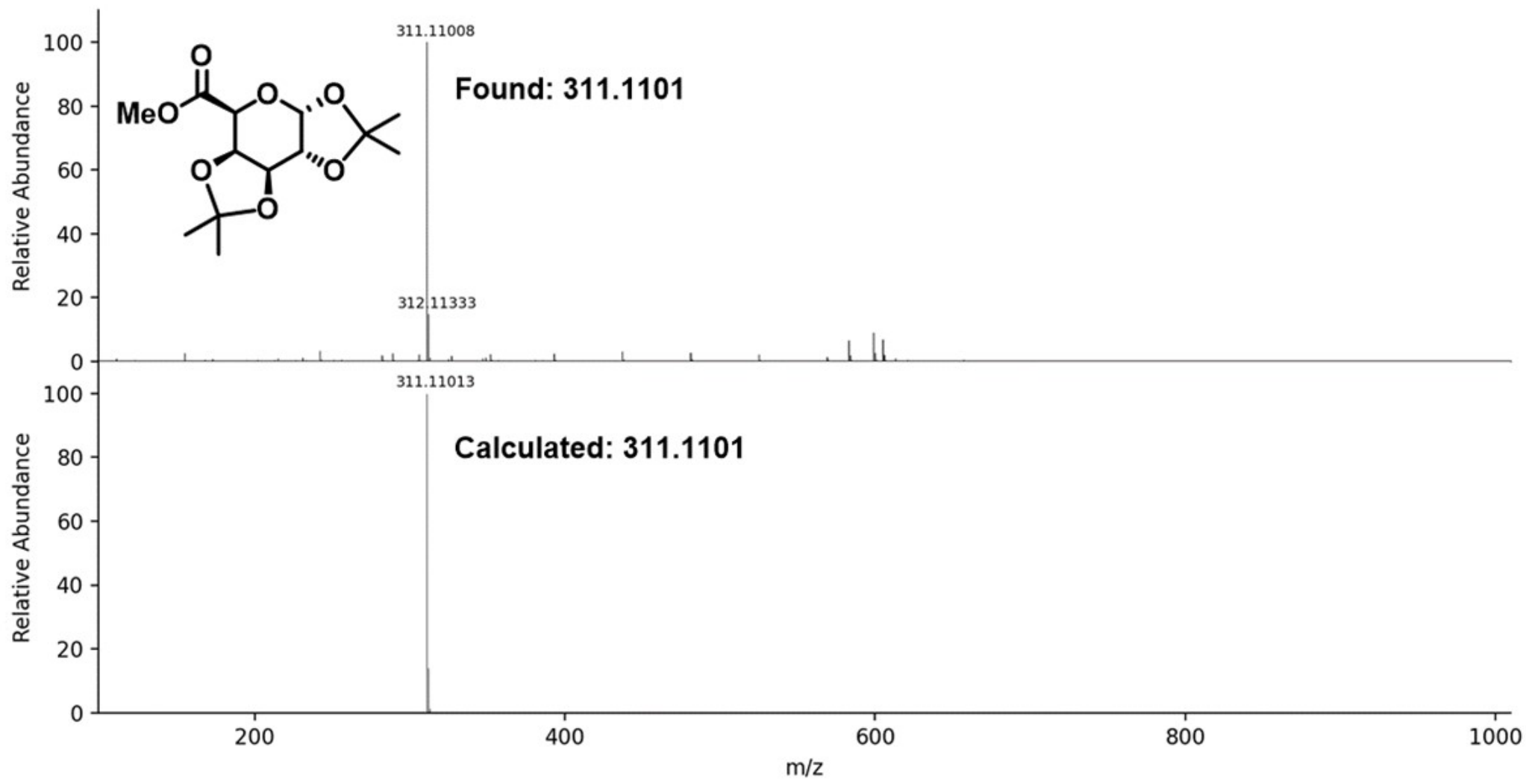
311.11008
Found: 311.1101
312.11333
311.11013
Calculated: 311.1101
Relative Abundance
m/z
MeO

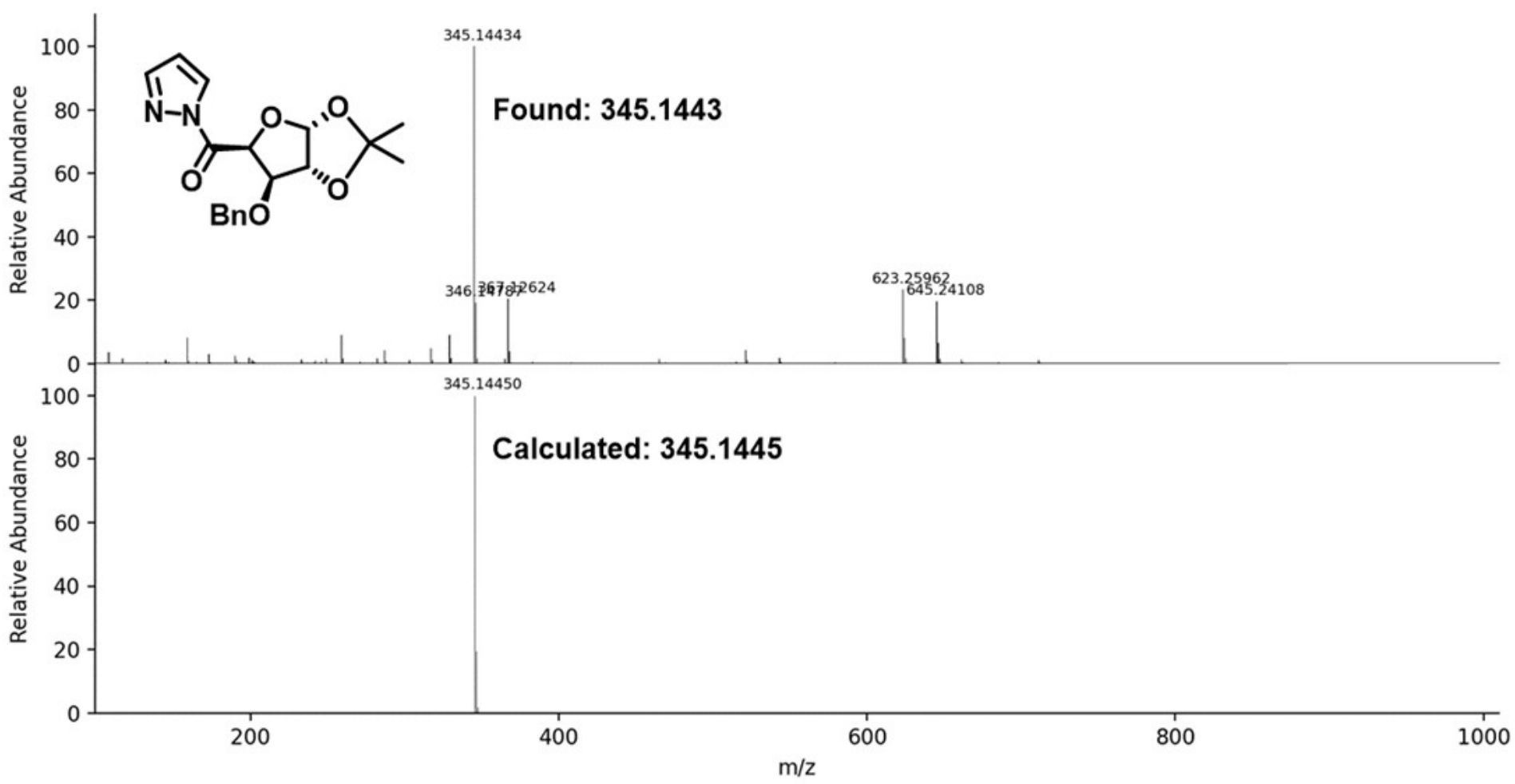
345.14434
Found: 345.1443
367.12624
623.25962
645.24108
345.14450
Calculated: 345.1445
Relative Abundance
m/z
BnO

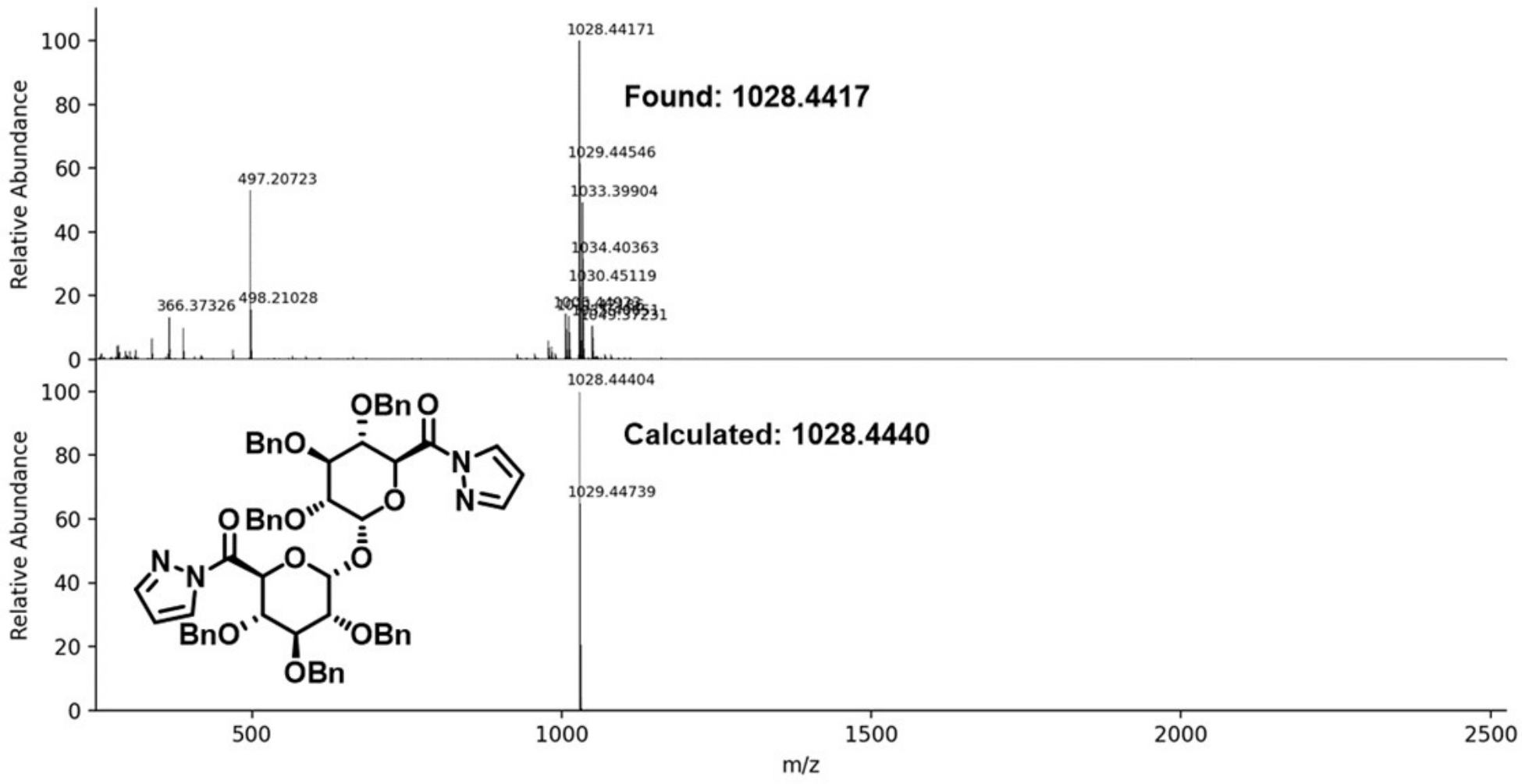

Found: 1028.4417
Calculated: 1028.4440
1028.44171
1029.44546
1033.39904
1034.40363
1030.45119
497.20723
366.37326
498.21028
1028.44404
1029.44739
Relative Abundance
m/z
BnO
OBn
O
N
N

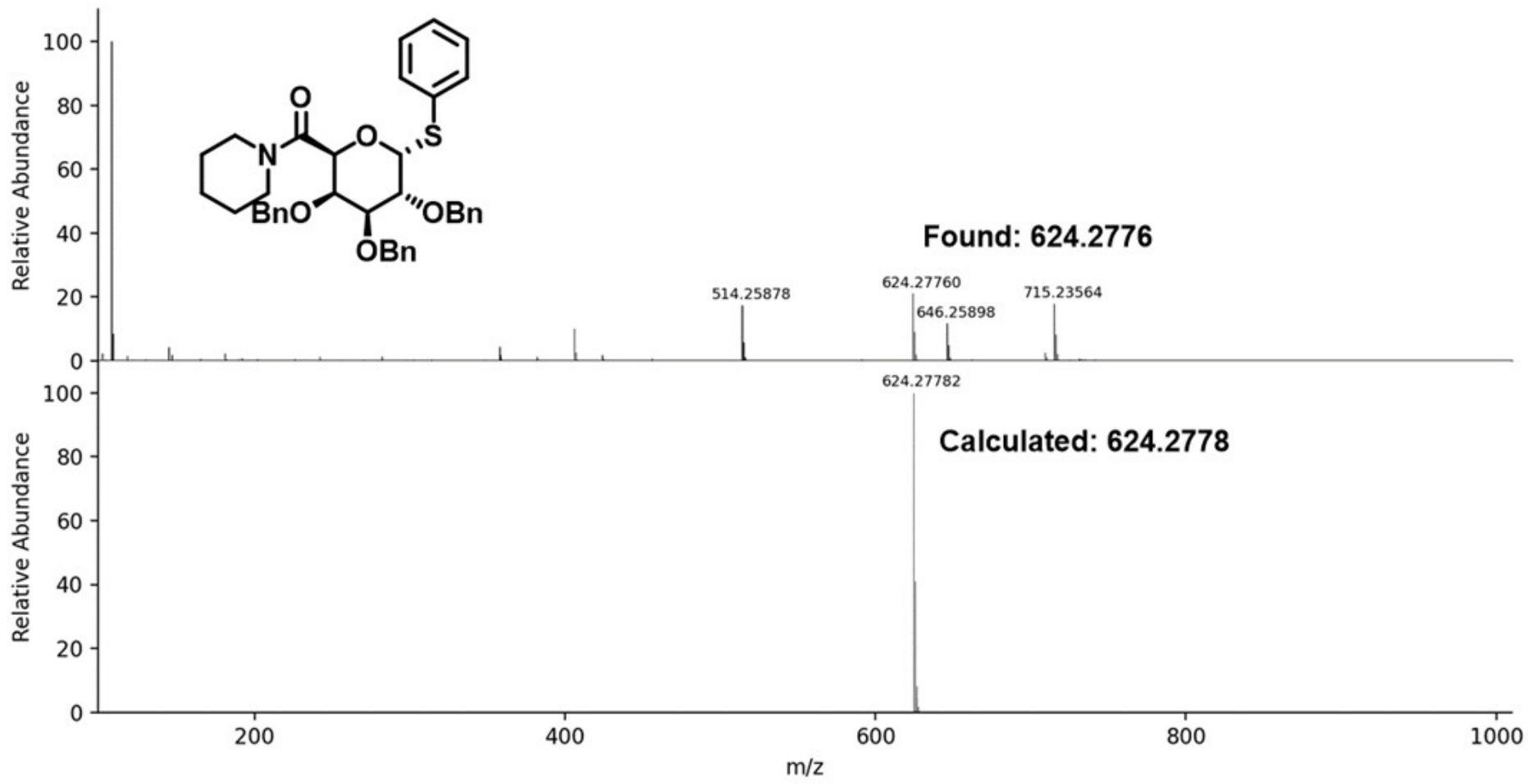

Found: 624.2776
Calculated: 624.2778
514.25878
624.27760
646.25898
715.23564
624.27782
Relative Abundance
m/z
S
O
N
BnO
OBn

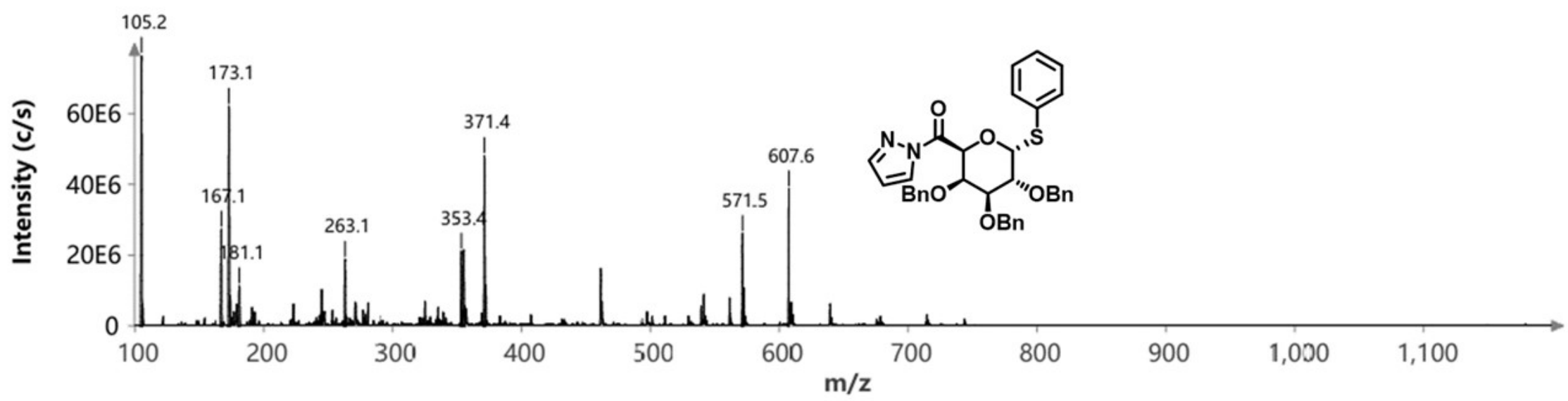

105.2
173.1
167.1
181.1
263.1
353.4
371.4
571.5
607.6
Intensity (c/s)
m/z
BnO
OBn
S

11.4 HPLC chromatogram

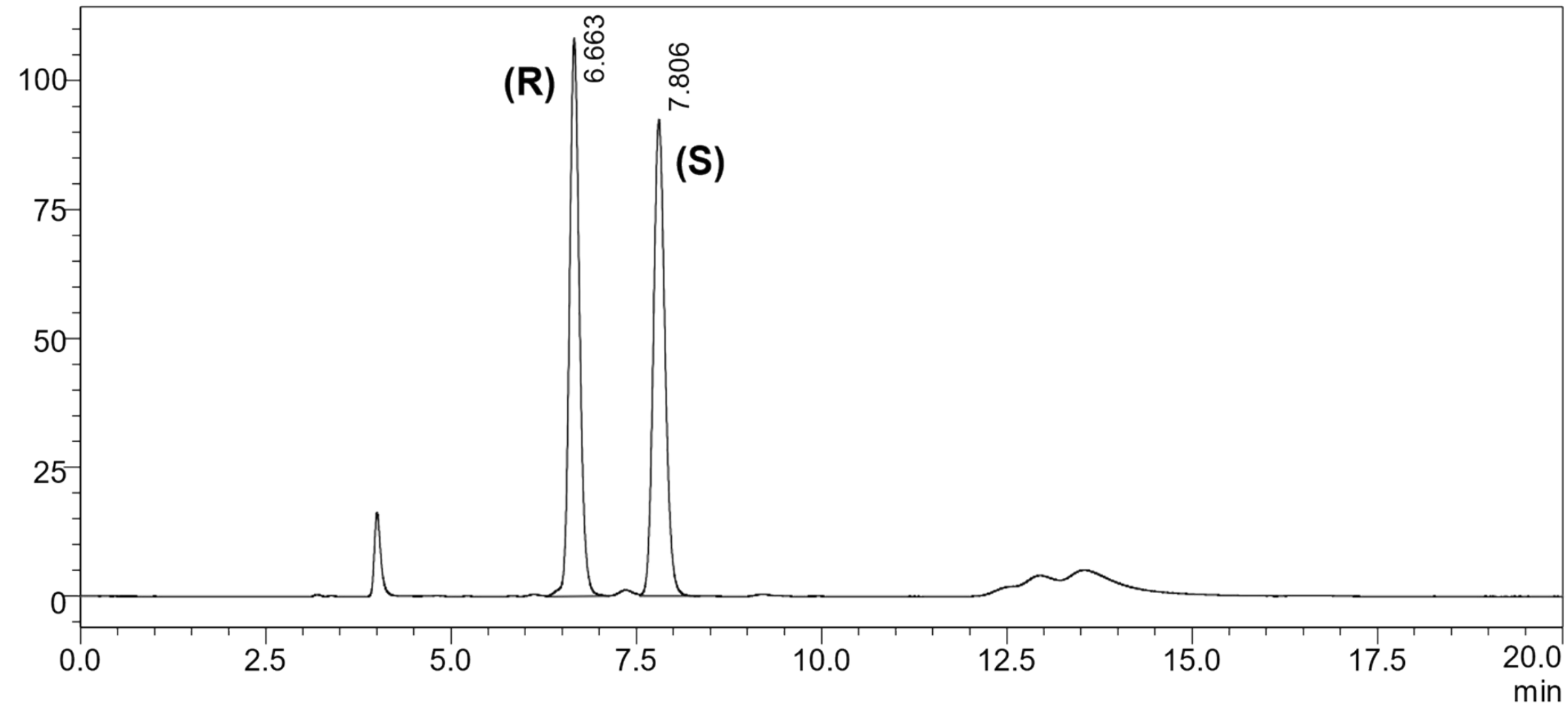

Peak#	Ret. Time	Area	Area%	Height	Height%	Area/Height
1	6.663	1017601	50.294	108054	53.909	9.417
2	7.806	1005703	49.706	92385	46.091	10.886
Total		2023305	100.000	200440	100.000	

Figure S41: HPLC chromatogram of *rac*-solketal ***2b***

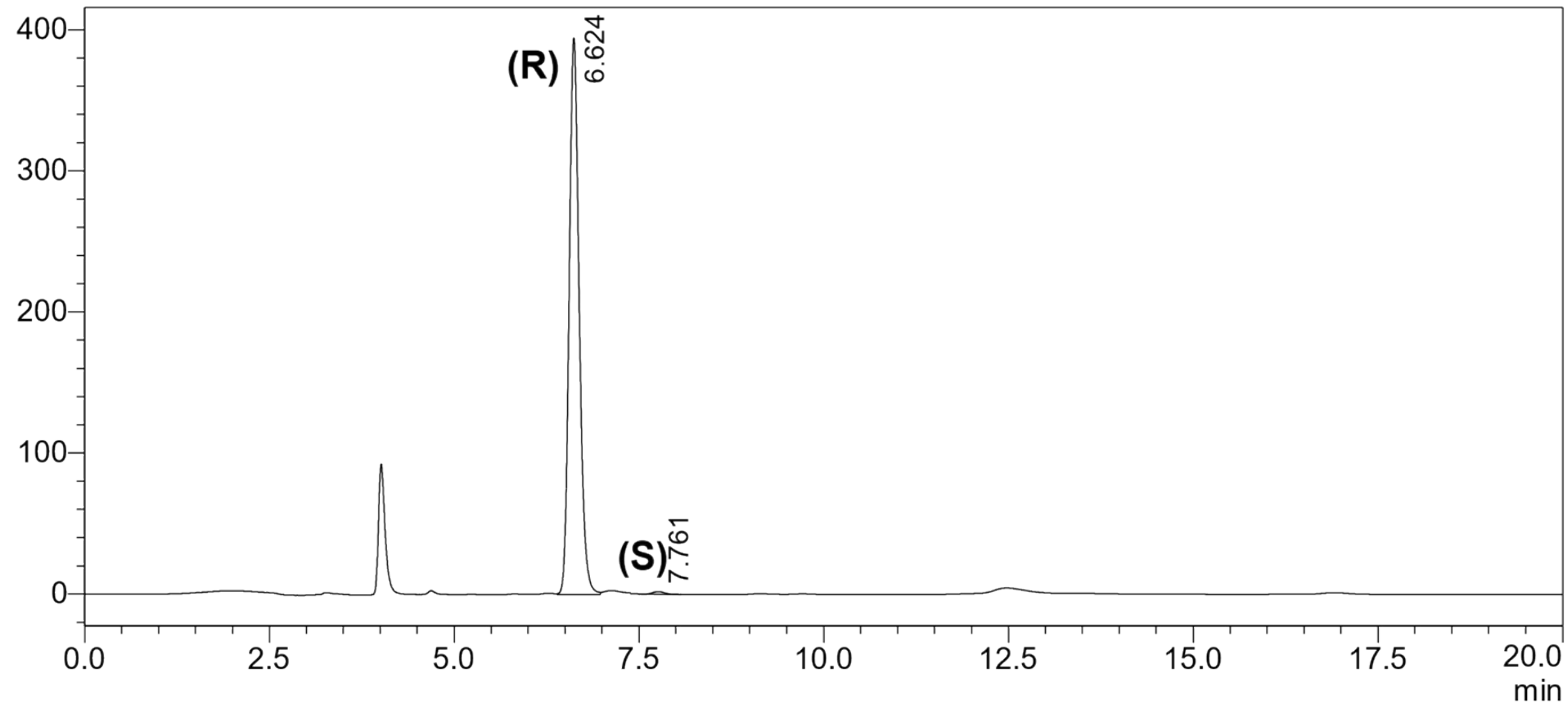

Peak#	Ret. Time	Area	Area%	Height	Height%	Area/Height
1	6.624	3681813	99.474	393131	99.525	9.365
2	7.761	19472	0.526	1877	0.475	10.371
Total		3701286	100.000	395008	100.000	

Figure S42: HPLC chromatogram of *(R)*-solketal ***2b***

References

1 J. M. Ovian, C. B. Kelly, V. A. Pistritto and N. E. Leadbeater, Accessing N-Acyl Azoles via Oxoammonium Salt-Mediated Oxidative Amidation, *Org. Lett.*, 2017, **19**, 1286–1289.

2 C. Kashima, H. Harada, I. Kita, I. Fukuchi and A. Hosomi, The Preparation of N-Acylpyrazoles and Their Behavior Toward Alcohols, *Synthesis (Stuttg).*, 1994, 61–65.

3 M. S. Shoshan, T. Vonderach, B. Hattendorf and H. Wennemers, Peptide-Coated Platinum Nanoparticles with Selective Toxicity against Liver Cancer Cells, *Angew. Chem. Int. Ed.*, 2019, **58**, 4901–4905.

4 J. Š. Raschmanová, M. Martinková, M. B. Pilátová, N. Nosálová, J. Kuchár and G. Bodnár, Synthesis and in vitro anticancer activity of penaresidin-related stereoisomeric analogues, *Carbohydr. Res.*, 2021, **508**, 108419.

5 A. Adibekian, P. Bindschädler, M. S. M. Timmer, C. Noti, N. Schützenmeister and P. H. Seeberger, De novo synthesis of uronic acid building blocks for assembly of heparin oligosaccharides, *Chem. Eur. J.*, 2007, **13**, 4510–4522.

6 S. R. Gilbertson and C. W. T. Chang, Synthesis of New Disugar Phosphine Ligands and Their Use in Asymmetric Hydrogenation, *J. Org. Chem.*, 2002, **60**, 6226–6228.

7 Z. T. Zhang, Y. Ma, N. L. Fan and X. G. Hu, Synthesis of (non-classical) C-acyl-glycosides via Liebeskind–Srogl coupling: Scope, limitation, improved synthesis and antioxidant activity of scleropentaside A, *Tetrahedron*, 2021, **92**, 132255.

8 M. Krumb, M. Jäger, A. Voss, L. Immig, K. Peters, D. Kowalczyk, A. Bufe, T. Opatz, O. Holst, C. Vogel and M. Peters, Total Synthesis of a Partial Structure from Arabinogalactan and Its Application for Allergy Prevention, *Chem. Eur. J.*, 2021, **27**, 928–933.

9 J. Dinkelaar, A. R. De Jong, R. Van Meer, M. Somers, G. Lodder, H. S. Overkleeft, J. D. C. Codée and G. A. Van Der Marel, Stereodirecting Effect of the Pyranosyl C-5 Substituent in Glycosylation Reactions, *J. Org. Chem.*, 2009, **74**, 4982–4991.
